# Supplementary material for: Lignans from the Twigs of Litsea cubeba and Their Bioactivities
Source: Molecules. 2019 Jan 16;24(2):306. doi: 10.3390/molecules24020306 (PMC6359749; doi:10.3390/molecules24020306)
Supplement: Supplementary file 1 [file molecules-24-00306-s001.pdf]

Lignans from the Twigs of *Litsea cubeba* and Their Bioactivities

Xiuting Li [1,†], Huan Xia [2,†], Lingyan Wang [2], Guiyang Xia [2], Yuhong Qu [2], Xiaoya Shang [3,*] and Sheng Lin [2,*]

[1] Beijing Advanced Innovation Center for Food Nutrition and Human Health, Beijing Technology and Business University, Beijing, 100048, People's Republic of China

[2] State Key Laboratory of Bioactive Substance and Function of Natural Medicines, Institute of Materia Medica, Chinese Academy of Medical Sciences and Peking Union Medical College, Beijing 100050, People's Republic of China

[3] Beijing Key Laboratory of Bioactive Substances and Functional Foods, Beijing Union University, Beijing 100023, People's Republic of China

Supplementary Information

* Corresponding authors. Tel: +86-10-62004533 (X.S.); +86-10-60212110. E-mail: shangxiaoya@buu.edu.cn. (X.Y. Shang) and lsznn@imm.ac.cn (S. Lin)

The List of Contents

no	Contents	Page
1	*In silico* prediction of ECD spectrum	5
2	**Table S1**. Cartesian Coordinates, Relative Energies, and Equilibrium Populations of Low-energy Conformers (>0.1%) of 8*R*,7'*S*,8'*R*-**7** in MeOH.	5-8
3	**Table S2**. Extracted heats and weighting factors of the optimized conformers of 8*R*,7'*S*,8'*R*-**7** at B3LYP/6-311+G(d,p) level in MeOH with PCM model	9
4	**Figure S1.** The experimental ECD spectrum of **7** (black), and the calculated ECD spectra of (8*R*,7′*S*,8′*R*)-**7** (red) and (8*S*,7′*R*,8′*S*)-**7** (blue)	10
5	**Figure S2**. The UV Spectrum of Compound **1** in MeOH	11
6	**Figure S3.** The ESI-Mass Spectrum of Compound **1** in MeOH	12
7	**Figure S4.** The HR-Mass Spectrum of Compound **1** in MeOH	13
8	**Figure S5.** The IR Spectrum of Compound **1**	14
9	**Figure S6.** The ^{1}H NMR Spectrum of Compound **1** in Acetone-d_6 (500MHz)	15
10	**Figure S7.** The ^{13}C NMR Spectrum of Compound **1** in Acetone-d_6 (125MHz)	16
11	**Figure S8.** The HSQC Spectrum of Compound **1** in Acetone-d_6 (500MHz)	17
12	**Figure S9.** The HMBC Spectrum of Compound **1** in Acetone-d_6 (500MHz)	18
13	**Figure S10.** The NOESY Spectrum of Compound **1** in Acetone-d_6 (500MHz)	19
14	**Figure S11.** The UV Spectra of Compound **2** in MeOH	20
15	**Figure S12.** The ESI-Mass Spectrum of Compound **2** in MeOH	21
16	**Figure S13.** The HR-Mass Spectrum of Caaompound **2** in MeOH	22
17	**Figure S14.** The IR spectrum of compound **2**	23
18	**Figure S15.** The ^{1}H NMR spectrum of compound **2** in Acetone-d_{66Y} (500MHz)	24
19	**Figure S16.** The ^{13}C NMR spectrum of compound **2** in Acetone-d_6 (125MHz)	25
20	**Figure S17.** The ^{1}H-^{1}H COSY Spectrum of Compound **2** in Acetone-d_6 (500MHz)	26
21	**Figure S18.** The HSQC Spectrum of Compound **2** in Acetone-d_6 (500MHz)	27
22	**Figure S19.** The HMBC Spectrum of Compound **2** in Acetone-d_6 (500MHz)	28
23	**Figure S20.** The 1D NOE Difference Spectrum of Compound **2** in Acetone-d_6 (500MHz)	29
24	**Figure S21.** The UV Spectra of Compound **3** in MeOH	30
25	**Figure S22.** The ESI-Mass spectrum of compound **3** in MeOH	31
26	**Figure S23.** The HR-Mass spectrum of compound **3** in MeOH	32
27	**Figure S24.** The IR spectrum of compound **3**	33
28	**Figure S25.** The ^{1}H NMR spectrum of compound **3** in Acetone-d_6 (600MHz)	34
29	**Figure S26.** The ^{13}C NMR spectrum of compound **3** in Acetone-d_6 (150MHz)	35
30	**Figure S27.** The DEPT Spectrum of Compound **3** in Acetone-d_6 (150MHz)	36
31	**Figure S28.** The ^{1}H-^{1}H COSY Spectrum of Compound **3** in Acetone-d_6 (600MHz)	37
32	**Figure S29.** The HSQC Spectrum of Compound **3** in Acetone-d_6 (600MHz)	38
33	**Figure S30.** The HMBC Spectrum of Compound **3** in Acetone-d_6 (600MHz)	39
34	**Figure S31.** The 1D NOE Difference Spectrum of Compound **3** in Acetone-d_6 (500MHz)	40
35	**Figure S32.** The UV Spectra of Compound **4** in MeOH	41
36	**Figure S33.** The ESI-Mass Spectrum of Compound **4** in MeOH	42
37	**Figure S34.** The HR-Mass Spectrum of Compound **4** in MeOH	43
38	**Figure S35.** The IR Spectrum of Compound **4**	44

39	**Figure S36.** The ^{1}H NMR Spectrum of Compound **4** in Acetone-d_6 (500MHz)	45
40	**Figure S37.** The ^{13}C NMR Spectrum of Compound **4** in Acetone-d_6 (125MHz)	46
41	**Figure S38.** The ^{1}H-^{1}H COSY Spectrum of Compound **4** in Acetone-d_6 (500MHz)	47
42	**Figure S39.** The HSQC Spectrum of Compound **4** in Acetone-d_6 (500MHz)	48
43	**Figure S40.** The HMBC Spectrum of Compound **4** in Acetone-d_6 (500MHz)	49
44	**Figure S41.** The UV Spectra of Compound **5** in MeOH	50
45	**Figure S42.** The HR-Mass Spectrum of Compound **5** in MeOH	51
46	**Figure S43.** The IR Spectrum of Compound **5**	52
47	**Figure S44.** The ^{1}H NMR spectrum of compound **5** in Acetone-d_6 (600MHz)	53
48	**Figure S45.** The ^{13}C NMR spectrum of compound **5** in Acetone-d_6 (150MHz)	54
49	**Figure S46.** The ^{1}H-^{1}H COSY Spectrum of Compound **5** in Acetone-d_6 (600MHz)	55
50	**Figure S47.** The HSQC Spectrum of Compound **5** in Acetone-d_6 (600MHz)	56
51	**Figure S48.** The HMBC Spectrum of Compound **5** in Acetone-d_6 (600MHz)	57
52	**Figure S49.** The HR-Mass Spectrum of Compound **6** in MeOH	58
53	**Figure S50.** The IR Spectrum of Compound **6**	59
54	**Figure S51.** The ^{1}H NMR Spectrum of Compound **6** in Acetone-d_6 (600MHz)	60
55	**Figure S52.** The ^{13}C NMR Spectrum of Compound **6** in Acetone-d_6 (600MHz)	61
56	**Figure S53.** The ^{1}H-^{1}H COSY Spectrum of Compound **6** in Acetone-d_6 (600MHz)	62
57	**Figure S54.** The HSQC Spectrum of Compound **6** in Acetone-d_6 (600MHz)	63
58	**Figure S55.** The HMBC Spectrum of Compound **6** in Acetone-d_6 (600MHz)	64
59	**Figure S56.** The UV and CD Spectra of Compound **7** in MeOH	65
60	**Figure S57.** The ESI-Mass spectrum of compound **7** in MeOH	66
61	**Figure S58.** The HR-Mass spectrum of compound **7** in MeOH	67
62	**Figure S59.** The IR spectrum of compound **7**	68
63	**Figure S60.** The ^{1}H NMR spectrum of compound **7** in Acetone-d_6 (500MHz)	69
64	**Figure S61.** The ^{13}C NMR spectrum of compound **7** in Acetone-d_6 (125MHz)	70
65	**Figure S62.** The ^{1}H-^{1}H COSY Spectrum of Compound **7** in Acetone-d_6 (500MHz)	71
66	**Figure S63.** The HSQC Spectrum of Compound **7** in Acetone-d_6 (500MHz)	72
67	**Figure S64.** The HMBC Spectrum of Compound **7** in Acetone-d_6 (500MHz)	73
68	**Figure S65.** The NOESY Spectrum of Compound **7** in Acetone-d_6 (500MHz)	74
69	**Figure S66.** The IR Spectrum of Compound **8**	75
70	**Figure S67.** The ^{1}H NMR Spectrum of Compound **8** in Acetone-d_6 (600MHz)	76
71	**Figure S68.** The ^{13}C NMR Spectrum of Compound **8** in Acetone-d_6 (150MHz)	77
72	**Figure S69.** The ^{1}H-^{1}H COSY Spectrum of Compound **8** in Acetone-d_6 (600MHz)	78
73	**Figure S70.** The HSQC Spectrum of Compound **8** in Acetone-d_6 (600MHz)	79
74	**Figure S71.** The HMBC Spectrum of Compound **8** in Acetone-d_6 (600MHz)	80
75	**Figure S72.** The NOESY Spectrum of Compound **8** in Acetone-d_6 (600MHz)	81
76	**Figure S73.** The HR-Mass Spectrum of Compound **9** in MeOH	82
77	**Figure S74.** The IR Spectrum of Compound **9**	83
78	**Figure S75.** The ^{1}H NMR Spectrum of Compound **9** in Acetone-d_6 (500MHz)	84
79	**Figure S76.** The ^{13}C NMR spectrum of compound **9** in Acetone-d_6 (500MHz)	85
80	**Figure S77.** The ^{1}H-^{1}H COSY Spectrum of Compound **9** in Acetone-d_6 (500MHz)	86
81	**Figure S78.** The HSQC Spectrum of Compound **9** in Acetone-d_6 (500MHz)	87

82	**Figure S79.** The HMBC Spectrum of Compound **9** in Acetone-d_6 (500MHz)	88
83	**Figure S80.** The NOESY Spectrum of Compound **9** in Acetone-d_6 (500MHz)	89

In silico prediction of ECD spectrum.

All calculations were performed using Gaussian 16.[1] Conformation search using molecular mechanics calculations was performed in DS (Discovery Studio) 2018 with 20 kcal mol^{-1} upper energy limit at best level. The stable (Equilibrium Populations of Low-energy >0.1%) conformers performed with the DS 2018 software package were further optimized by using the TDDFT method at the B3LYP/6-31G(d, p) level, and the frequency was calculated at the same level of theory. For all optimized structures, vibrational spectra were calculated to ensure that no imaginary frequencies for energy minimum were obtained. The stable conformers were subjected to ECD calculation by the TDDFT method at the B3LYP/6-311G+(d,p) level with the CPCM model in MeOH. ECD spectra of different conformers were simulated using SpecDis 1.71[2] with a half-bandwidth of 0.3 eV, and the final calculated ECD spectra were obtained according to the Boltzmann-calculated contribution of each con-former. The calculated ECD spectra were compared with the experimental data.

Table S1. Cartesian Coordinates, Relative Energies, and Equilibrium Populations of Low-energy Conformers (>0.1%) of 8*R*,7'*S*,8'*R*-7 in MeOH.

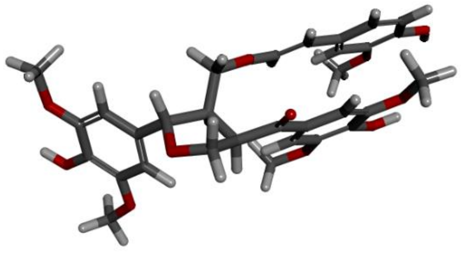<br>Conformation 1<br>ΔE = 0.00 kcal/mol<br>P(%) = 83.07%				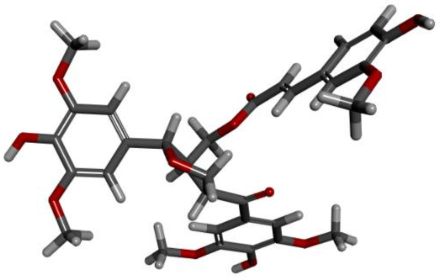<br>Conformation 2<br>ΔE = 0.99 kcal/mol<br>P(%) = 15.67%			
C	-2.967	0.803	2.543	C	-1.684	-0.931	0.860
O	-2.668	1.805	3.487	O	-2.517	-0.347	-0.115
C	-1.982	2.855	2.857	C	-1.777	0.569	-0.880
C	-0.978	2.103	1.986	C	-0.974	1.313	0.187
C	-1.843	0.919	1.483	C	-0.641	0.170	1.179
C	-3.077	-0.548	3.195	C	-2.485	-1.407	2.041
C	-1.985	-1.104	3.887	C	-2.500	-2.772	2.381
C	-2.069	-2.367	4.495	C	-3.243	-3.236	3.488
C	-3.287	-3.093	4.407	C	-3.978	-2.313	4.259
C	-4.394	-2.548	3.715	C	-3.975	-0.937	3.934
C	-4.275	-1.276	3.115	C	-3.226	-0.499	2.820
C	-0.284	2.950	0.931	C	0.203	2.117	-0.344
C	0.933	2.431	0.195	C	0.972	3.053	0.562

C	-2.494	1.115	0.099	C	0.780	-0.400	1.063
O	-1.542	0.966	-0.958	O	1.042	-0.778	-0.290
C	1.486	1.171	0.493	C	2.109	3.724	0.076
C	2.615	0.687	-0.204	C	2.848	4.600	0.901
C	3.199	1.485	-1.205	C	2.435	4.802	2.232
C	2.661	2.751	-1.522	C	1.297	4.139	2.741
C	1.530	3.213	-0.812	C	0.574	3.270	1.895
O	4.256	1.019	-1.851	O	3.136	5.624	2.995
O	-3.394	-4.284	4.971	O	-4.670	-2.762	5.293
O	-0.711	4.058	0.696	O	0.516	2.003	-1.509
C	-1.096	-0.275	-1.218	C	2.255	-1.284	-0.559
C	-0.011	-0.442	-2.201	C	2.569	-1.654	-1.952
O	-1.526	-1.273	-0.684	O	3.113	-1.456	0.277
C	0.385	0.548	-3.030	C	1.733	-1.410	-2.985
C	1.543	0.465	-3.993	C	2.000	-1.771	-4.426
C	2.366	-0.678	-4.089	C	1.038	-1.430	-5.398
C	3.465	-0.712	-4.979	C	1.233	-1.743	-6.763
C	3.728	0.417	-5.786	C	2.417	-2.410	-7.149
C	2.904	1.551	-5.698	C	3.378	-2.751	-6.183
C	1.819	1.574	-4.811	C	3.175	-2.435	-4.831
O	4.744	0.435	-6.632	O	2.647	-2.726	-8.412
O	3.158	-0.505	0.033	O	3.929	5.253	0.482
C	2.615	-1.417	0.993	C	4.438	5.121	-0.849
O	-0.969	-2.788	5.118	O	-3.286	-4.517	3.847
C	-0.881	-4.036	5.810	C	-2.572	-5.537	3.140
O	-5.512	-3.268	3.658	O	-4.682	-0.110	4.699
C	-6.687	-2.812	2.980	C	-4.729	1.301	4.463
O	3.244	3.452	-2.492	O	0.953	4.365	4.007
C	2.752	4.724	-2.922	C	-0.178	3.738	4.620
O	4.272	-1.764	-5.092	O	0.350	-1.437	-7.710
C	4.106	-2.948	-4.306	C	-0.875	-0.752	-7.425
H	-3.919	1.096	2.115	H	-1.204	-1.766	0.365
H	-2.686	3.427	2.267	H	-1.142	0.017	-1.560
H	-1.523	3.501	3.591	H	-2.437	1.206	-1.450
H	-0.222	1.718	2.658	H	-1.661	1.998	0.667
H	-1.230	0.029	1.458	H	-0.753	0.545	2.187
H	-1.060	-0.548	3.951	H	-1.929	-3.462	1.777
H	-5.109	-0.842	2.583	H	-3.210	0.546	2.547
H	-2.924	2.115	0.024	H	0.893	-1.274	1.707
H	-3.312	0.411	-0.066	H	1.519	0.339	1.376
H	1.041	0.563	1.264	H	2.414	3.560	-0.947
H	1.108	4.180	-1.040	H	-0.299	2.766	2.278
H	4.701	1.505	-2.559	H	2.913	5.811	3.917
H	-4.198	-4.822	4.943	H	-5.194	-2.187	5.868
H	0.465	-1.410	-2.223	H	3.521	-2.137	-2.113

H	-0.168	1.477	-2.994	H	0.797	-0.917	-2.760
H	2.161	-1.539	-3.474	H	0.140	-0.921	-5.081
H	3.105	2.413	-6.317	H	4.283	-3.261	-6.480
H	1.198	2.458	-4.761	H	3.939	-2.716	-4.123
H	5.351	-0.310	-6.742	H	2.029	-2.521	-9.128
H	2.640	-0.976	1.991	H	4.720	4.085	-1.044
H	3.221	-2.323	0.997	H	5.324	5.748	-0.948
H	1.591	-1.683	0.723	H	3.692	5.454	-1.573
H	-1.604	-4.070	6.627	H	-2.920	-5.594	2.108
H	0.120	-4.127	6.234	H	-2.759	-6.495	3.625
H	-1.037	-4.865	5.118	H	-1.500	-5.335	3.168
H	-7.050	-1.889	3.433	H	-3.729	1.731	4.539
H	-7.461	-3.574	3.072	H	-5.364	1.761	5.221
H	-6.475	-2.657	1.920	H	-5.156	1.506	3.480
H	1.732	4.626	-3.298	H	-1.095	4.018	4.098
H	3.387	5.089	-3.730	H	-0.248	4.078	5.653
H	2.786	5.440	-2.100	H	-0.057	2.654	4.617
H	4.199	-2.712	-3.244	H	-1.488	-1.342	-6.742
H	4.889	-3.658	-4.575	H	-1.424	-0.615	-8.357
H	3.136	-3.404	-4.510	H	-0.666	0.230	-6.996
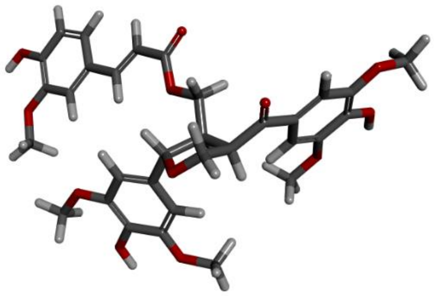<br>**Conformation 3**<br>**ΔE = 3.05 kcal/mol**<br>**P(%) = 0.49%**				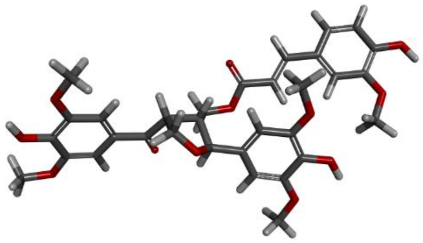<br>**Conformation 4**<br>**ΔE = 3.68 kcal/mol**<br>**P(%) = 0.17%**			
C	-2.174	0.581	0.532	C	-0.546	0.648	1.309
O	-3.223	1.161	1.277	O	0.174	0.620	2.523
C	-2.982	2.533	1.452	C	1.327	1.434	2.444
C	-1.491	2.561	1.782	C	1.149	2.304	1.189
C	-0.958	1.511	0.779	C	0.453	1.247	0.303
C	-1.957	-0.848	0.946	C	-1.108	-0.706	0.961
C	-2.147	-1.892	0.026	C	-0.266	-1.757	0.553
C	-1.951	-3.235	0.391	C	-0.797	-3.014	0.186
C	-1.544	-3.535	1.720	C	-2.187	-3.222	0.268
C	-1.343	-2.494	2.657	C	-3.049	-2.188	0.694
C	-1.557	-1.158	2.258	C	-2.496	-0.933	1.035
C	-0.852	3.940	1.739	C	0.406	3.602	1.491
C	0.551	4.159	2.260	C	1.075	4.708	2.285

C	-0.465	2.111	-0.545	C	-0.180	1.712	-1.019
O	-0.151	1.058	-1.462	O	-0.576	0.580	-1.806
C	1.133	5.438	2.187	C	2.431	4.631	2.652
C	2.431	5.687	2.664	C	3.052	5.665	3.388
C	3.171	4.617	3.232	C	2.294	6.796	3.769
C	2.606	3.323	3.311	C	0.923	6.889	3.413
C	1.298	3.109	2.824	C	0.339	5.844	2.673
O	4.394	4.822	3.693	O	2.872	7.767	4.459
O	-1.350	-4.789	2.091	O	-2.670	-4.408	-0.062
O	-1.487	4.869	1.293	O	-0.727	3.750	1.090
C	0.317	1.427	-2.665	C	0.390	-0.124	-2.423
C	0.577	0.389	-3.681	C	0.024	-1.443	-2.976
O	0.544	2.576	-2.972	O	1.533	0.259	-2.541
C	0.236	-0.909	-3.524	C	0.752	-2.094	-3.911
C	0.471	-1.999	-4.544	C	0.438	-3.474	-4.440
C	0.041	-3.308	-4.252	C	-0.467	-4.336	-3.783
C	0.239	-4.368	-5.168	C	-0.757	-5.621	-4.303
C	0.878	-4.099	-6.399	C	-0.124	-6.039	-5.494
C	1.304	-2.794	-6.696	C	0.788	-5.189	-6.141
C	1.105	-1.751	-5.780	C	1.069	-3.917	-5.618
O	1.091	-5.053	-7.290	O	-0.369	-7.226	-6.022
O	2.866	6.940	2.539	O	4.335	5.623	3.744
C	4.162	7.375	2.960	C	5.181	4.510	3.432
O	-2.170	-4.136	-0.566	O	-0.042	-4.021	-0.244
C	-2.067	-5.547	-0.362	C	1.379	-3.912	-0.374
O	-0.956	-2.819	3.889	O	-4.354	-2.447	0.753
C	-0.696	-1.836	4.898	C	-5.308	-1.482	1.207
O	3.340	2.351	3.849	O	0.117	7.903	3.728
C	2.874	1.002	3.955	C	0.537	9.049	4.476
O	-0.151	-5.617	-4.926	O	-1.604	-6.464	-3.718
C	-0.809	-5.993	-3.712	C	-2.262	-6.159	-2.485
H	-2.485	0.635	-0.503	H	-1.353	1.353	1.459
H	-3.206	3.047	0.527	H	2.178	0.777	2.327
H	-3.607	2.927	2.241	H	1.457	1.986	3.363
H	-1.383	2.172	2.786	H	2.105	2.546	0.748
H	-0.126	0.980	1.222	H	1.214	0.521	0.051
H	-2.452	-1.660	-0.985	H	0.799	-1.589	0.519
H	-1.415	-0.347	2.957	H	-3.135	-0.125	1.358
H	0.429	2.714	-0.380	H	0.502	2.339	-1.596
H	-1.232	2.749	-0.988	H	-1.074	2.304	-0.829
H	0.574	6.256	1.756	H	3.007	3.765	2.365
H	0.864	2.124	2.887	H	-0.703	5.923	2.400
H	4.941	4.131	4.091	H	3.801	7.755	4.728
H	-1.071	-5.049	2.980	H	-3.612	-4.626	-0.034
H	1.060	0.725	-4.586	H	-0.886	-1.879	-2.590

H	-0.244	-1.188	-2.595	H	1.621	-1.589	-4.311
H	-0.448	-3.493	-3.306	H	-0.944	-4.013	-2.872
H	1.790	-2.589	-7.639	H	1.277	-5.513	-7.049
H	1.451	-0.765	-6.050	H	1.775	-3.280	-6.132
H	0.828	-5.973	-7.148	H	-0.985	-7.860	-5.630
H	4.277	7.240	4.036	H	6.176	4.705	3.831
H	4.259	8.436	2.734	H	4.792	3.599	3.892
H	4.938	6.836	2.414	H	5.258	4.386	2.350
H	-1.042	-5.819	-0.105	H	1.634	-3.125	-1.086
H	-2.334	-6.052	-1.290	H	1.832	-3.707	0.597
H	-2.763	-5.870	0.414	H	1.768	-4.859	-0.749
H	0.111	-1.173	4.579	H	-6.301	-1.928	1.172
H	-0.387	-2.346	5.810	H	-5.091	-1.191	2.236
H	-1.599	-1.261	5.107	H	-5.296	-0.606	0.556
H	1.978	0.957	4.577	H	0.874	8.750	5.470
H	3.652	0.399	4.423	H	-0.315	9.719	4.590
H	2.667	0.594	2.964	H	1.324	9.583	3.942
H	-0.163	-5.795	-2.855	H	-2.887	-5.272	-2.599
H	-1.025	-7.061	-3.745	H	-1.523	-6.007	-1.696
H	-1.750	-5.450	-3.610	H	-2.897	-7.001	-2.210

Table S2. Extracted heats and weighting factors of the optimized conformers of 8*R*,7'*S*,8'*R*-7 at B3LYP/6-311+G(d,p) level in MeOH with PCM model

		B3LYP/6-311+G(d,p)	
	Conformer	Extracted heats	Boltzmann-calculated contribution(%)
8*R*,7'*S*,8'*R*-7	1	-2142.6500828	9.08
	2	-2142.6522415	89.52
	3	-2142.6458916	0.11
	4	-2142.6482445	1.29

Figure S1. The experimental ECD spectrum of 7 (black), and the calculated ECD spectra of (8*R*,7′*S*,8′*R*)-7 (red) and (8*S*,7′*R*,8′*S*)-7 (blue)

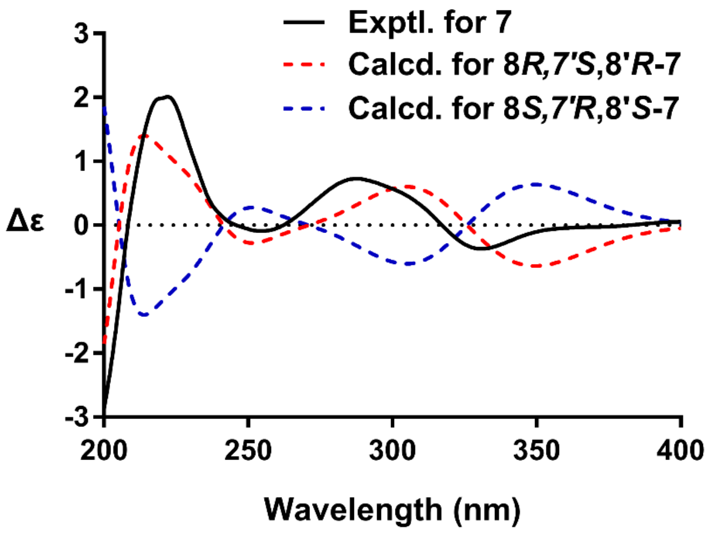

1. Gaussian 16, Revision A.03, M. J. Frisch, G. W. Trucks, H. B. Schlegel, G. E. Scuseria, M. A. Robb, J. R. Cheeseman, G. Scalmani, V. Barone, G. A. Petersson, H. Nakatsuji, X. Li, M. Caricato, A. V. Marenich, J. Bloino, B. G. Janesko, R. Gomperts, B. Mennucci, H. P. Hratchian, J. V. Ortiz, A. F. Izmaylov, J. L. Sonnenberg, D. Williams-Young, F. Ding, F. Lipparini, F. Egidi, J. Goings, B. Peng, A. Petrone, T. Henderson, D. Ranasinghe, V. G. Zakrzewski, J. Gao, N. Rega, G. Zheng, W. Liang, M. Hada, M. Ehara, K. Toyota, R. Fukuda, J. Hasegawa, M. Ishida, T. Nakajima, Y. Honda, O. Kitao, H. Nakai, T. Vreven, K. Throssell, J. A. Montgomery, Jr., J. E. Peralta, F. Ogliaro, M. J. Bearpark, J. J. Heyd, E. N. Brothers, K. N. Kudin, V. N. Staroverov, T. A. Keith, R. Kobayashi, J. Normand, K. Raghavachari, A. P. Rendell, J. C. Burant, S. S. Iyengar, J. Tomasi, M. Cossi, J. M. Millam, M. Klene, C. Adamo, R. Cammi, J. W. Ochterski, R. L. Martin, K. Morokuma, O. Farkas, J. B. Foresman, and D. J. Fox, Gaussian, Inc., Wallingford CT, **2016**.

2. T. Bruhn, A. Schaumlöffel, Y. Hemberger, G. Pescitelli, SpecDis version 1.71, Berlin, Germany, **2017**, http:/specdis-software.jimdo.com.

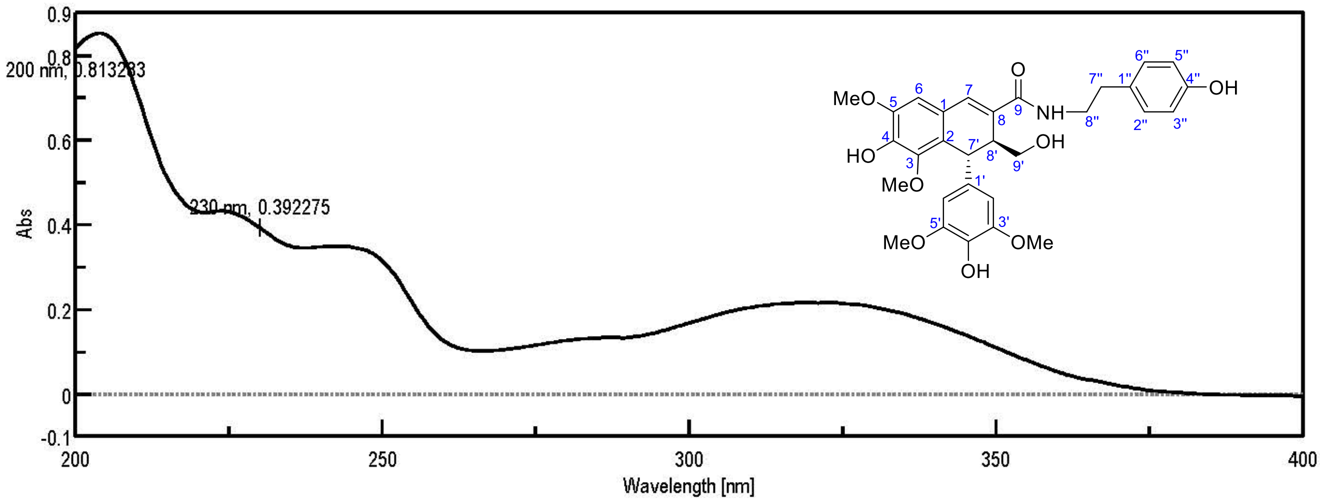

Figure S2. The UV Spectrum of Compound 1 in MeOH

Single Mass Spectrum Deconvolution Report

**Analysis Name:**	linsh132.d	**Instrument:**	LC-MSD-Trap-SL	**Print Date:**	8/28/2012 2:21:43 PM
**Method:**	standby.m	**Operator:**	Operator	**Acq. Date:**	8/28/2012 1:59:24 PM
**Sample Name:**	sjj-63				
**Analysis Info:**					

Acquisition Parameter:

Mass Range Mode	Std/Normal	Trap Drive	45.5	Scan Begin	100 m/z
Ion Polarity	Positive	Octopole RF Amplitude	152.8 Vpp	Scan End	700 m/z
Ion Source Type	ESI	Capillary Exit	-102.3 Volt	Averages	5 Spectra
Dry Temp (Set)	325 °C	Skimmer	-40.0 Volt	Max. Accu Time	200000 µs
Nebulizer (Set)	15.00 psi	Oct 1 DC	-12.00 Volt	ICC Target	100000
Dry Gas (Set)	5.00 l/min	Oct 2 DC	-1.70 Volt	Charge Control	on

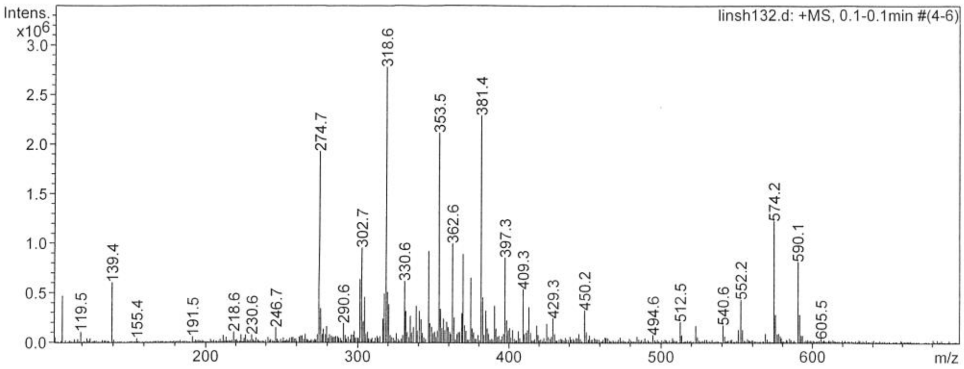

Component	Molecular Mass	Molecule	Absolute Abundance	Relative Abundance

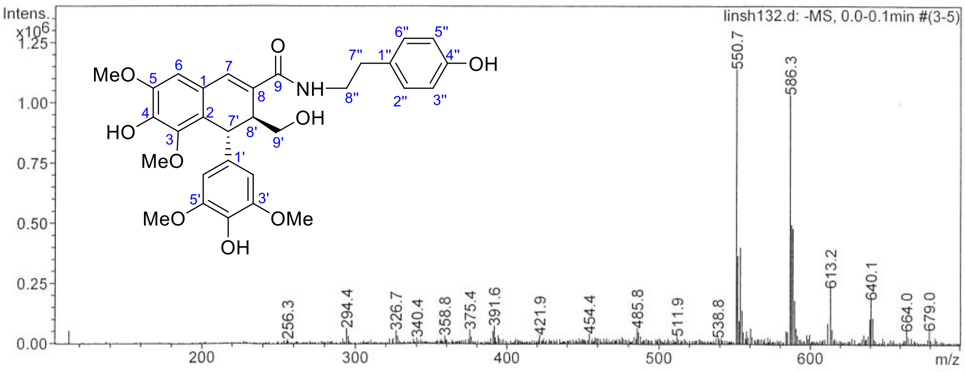

Component	Molecular Mass	Molecule	Absolute Abundance	Relative Abundance

MSD Trap Report v 4 (A4-Opt2) Page 1 of 1 Agilent Technologies

Figure S3. The ESI-Mass Spectrum of Compound 1 in MeOH

Qualitative Analysis Report

Fragmentor Voltage	Collision Energy	Ionization Mode
135	0	Esi

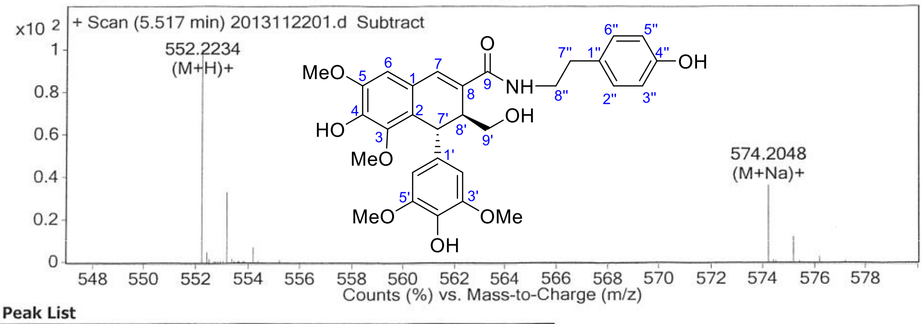

Peak List

m/z	z	Abund	Formula	Ion
552.2234	1	606689	C30 H34 N O9	(M+H)+
553.2263	1	201355	C30 H34 N O9	(M+H)+
554.2283	1	43216	C30 H34 N O9	(M+H)+
574.2048	1	221487	C30 H33 N Na O9	(M+Na)+
575.2083	1	72360	C30 H33 N Na O9	(M+Na)+
590.1784		84645		
1125.4193	1	89991		
1126.423	1	55801		

Formula Calculator Element Limits

Element	Min	Max
C	3	100
H	0	500
O	0	90
N	0	5
S	0	5
Cl	0	2
Br	0	0
Si	0	0
F	0	0
P	0	0

Formula Calculator Results

Formula	Best	Mass	Tgt Mass	Diff (ppm)	Ion Species	Score
C30 H33 N O9	TRUE	551.2161	551.2155	-1.12	C30 H34 N O9	99.96
C31 H29 N5 O5		551.2162	551.2169	1.29	C31 H30 N5 O5	99.82
C27 H37 N O9 S		551.2162	551.2189	4.99	C27 H38 N O9 S	98.77
C35 H29 N5 S		551.2162	551.2144	-3.25	C35 H30 N5 S	98.62
C34 H33 N O4 S		551.2162	551.213	-5.66	C34 H34 N O4 S	98.55
C22 H37 N3 O11 S		551.2162	551.2149	-2.32	C22 H38 N3 O11 S	98.16
C31 H37 N O4 S2		551.2162	551.2164	0.45	C31 H38 N O4 S2	97.8
C32 H33 N5 S2		551.2162	551.2177	2.86	C32 H34 N5 S2	97.74
C30 H33 N O9	TRUE	551.2155	551.2155	-0.02	C30 H33 N Na O9	99.98
C31 H29 N5 O5		551.2156	551.2169	2.39	C31 H29 N5 Na O5	99.71
C34 H33 N O4 S		551.2155	551.213	-4.57	C34 H33 N Na O4 S	98.67
C35 H29 N5 S		551.2156	551.2144	-2.16	C35 H29 N5 Na S	98.6
C22 H37 N3 O11 S		551.2156	551.2149	-1.23	C22 H37 N3 Na O11 S	98.32
C31 H37 N O4 S2		551.2156	551.2164	1.54	C31 H37 N Na O4 S2	97.7
C32 H33 N5 S2		551.2156	551.2177	3.95	C32 H33 N5 Na S2	97.54
C26 H37 N3 O6 S2		551.2156	551.2124	-5.77	C26 H37 N3 Na O6 S2	97.11
C18 H37 N3 O16		551.2156	551.2174	3.32	C18 H37 N3 Na O16	97.06

--- End Of Report ---

Figure S4. The HR-Mass Spectrum of Compound 1 in MeOH

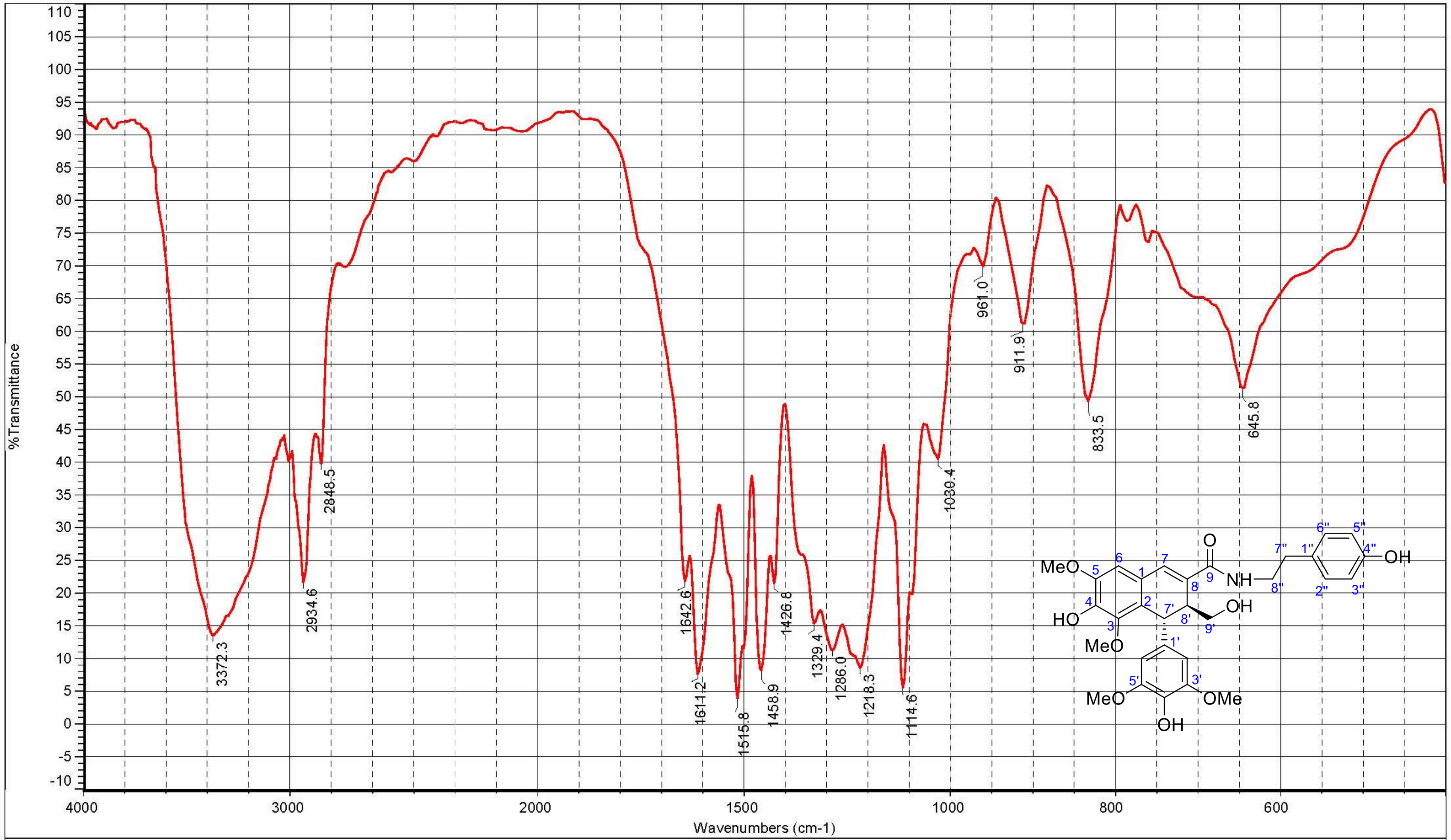

日期: 星期四 1月 16 13:07:27 2014 (GMT+08:00) Sample Name: sjj - 63 (显微镜透射法 FT- IR Microscope Transmission)

扫描次数: 100 傅立叶变换显微镜红外(FT-IR Microscope)：Centaurμs

分辨率: 8.000 美国热电公司(Thermo)傅立叶变换红外光谱仪:Nicolet 5700

Figure S5. The IR Spectrum of Compound 1

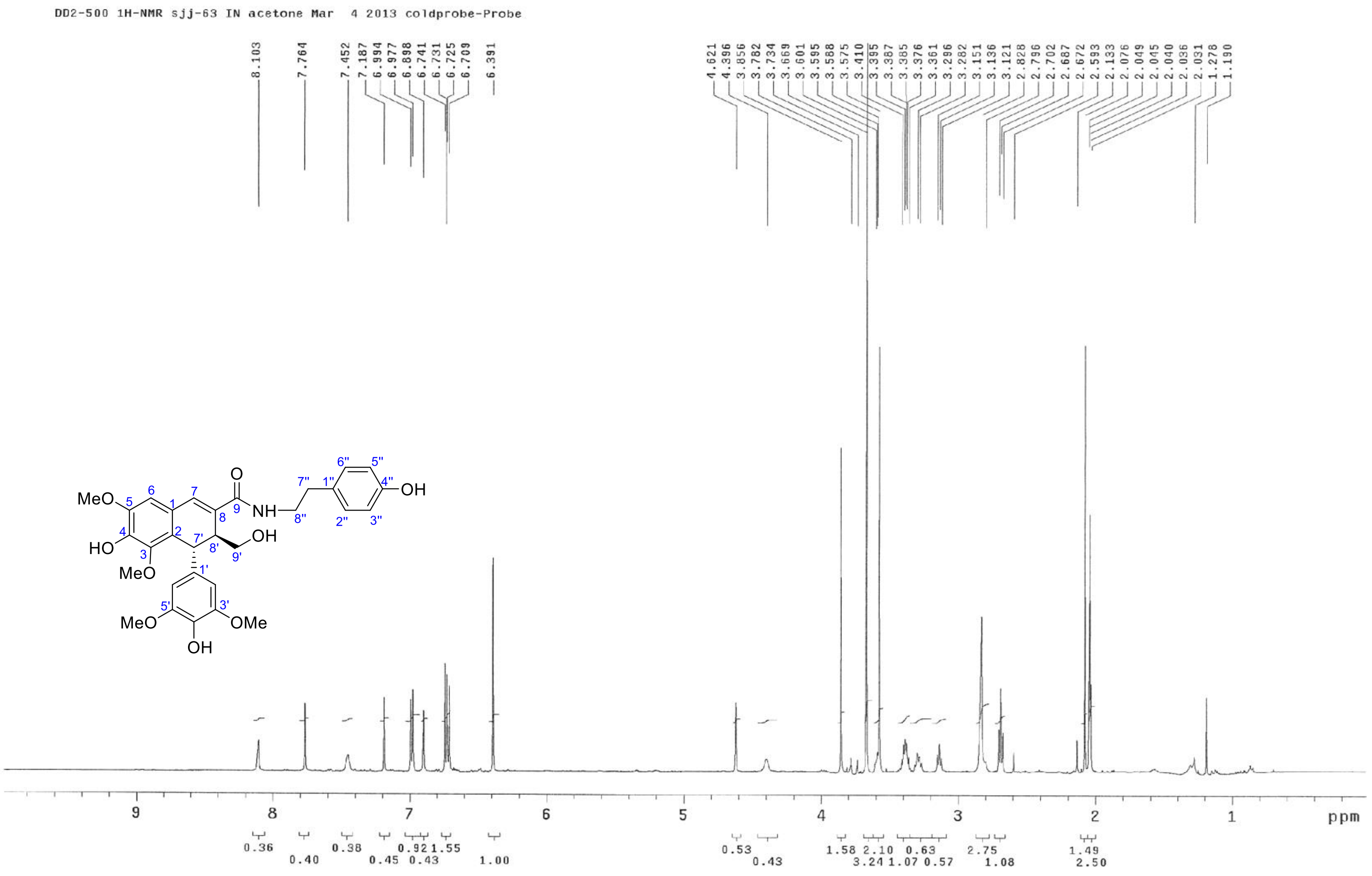

Figure S6. The ^{1}H NMR Spectrum of Compound 1 in Acetone-d_6 (500 MHz)

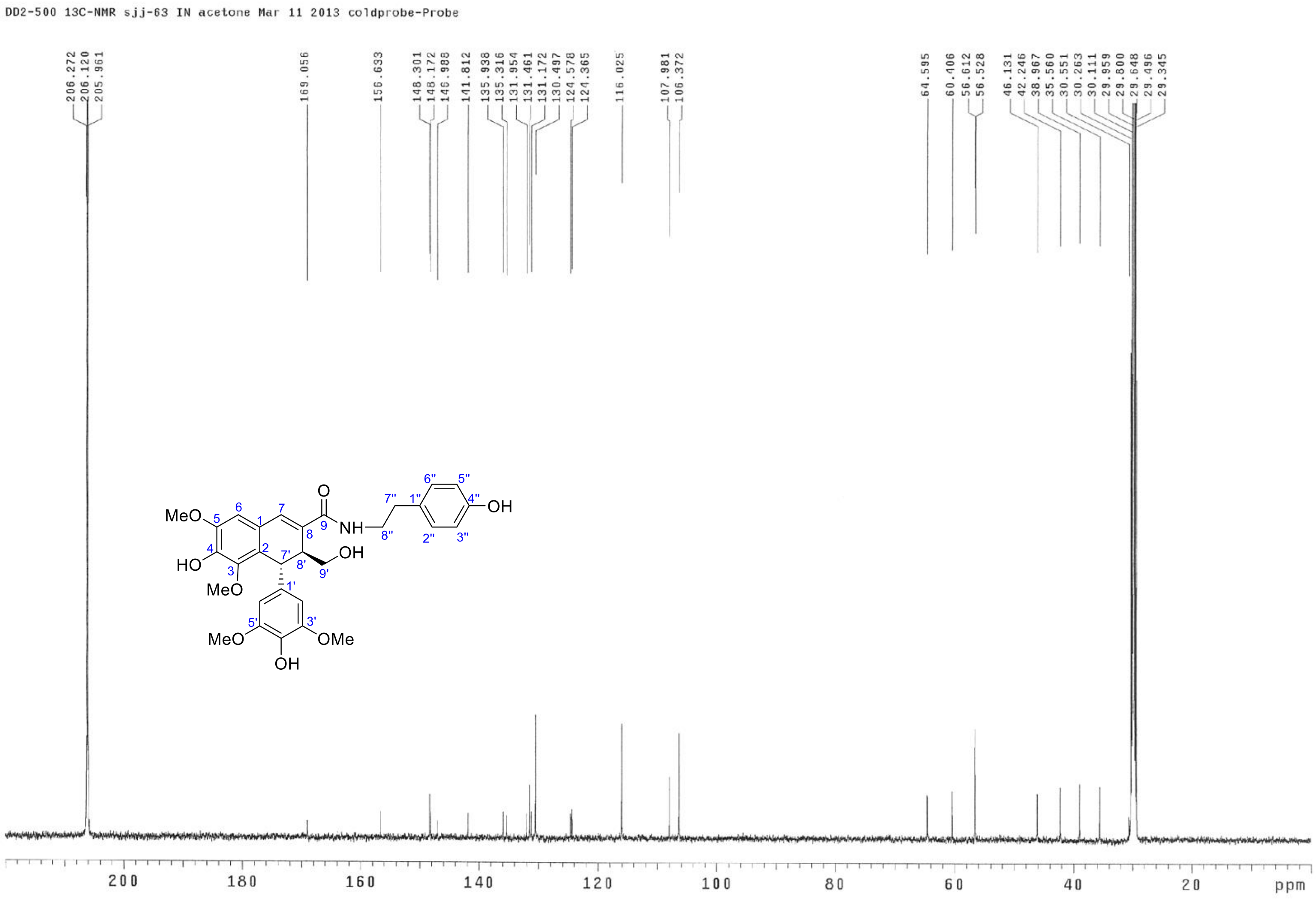

Figure S7. The ^{13}C NMR Spectrum of Compound 1 in Acetone-d_6 (125 MHz)

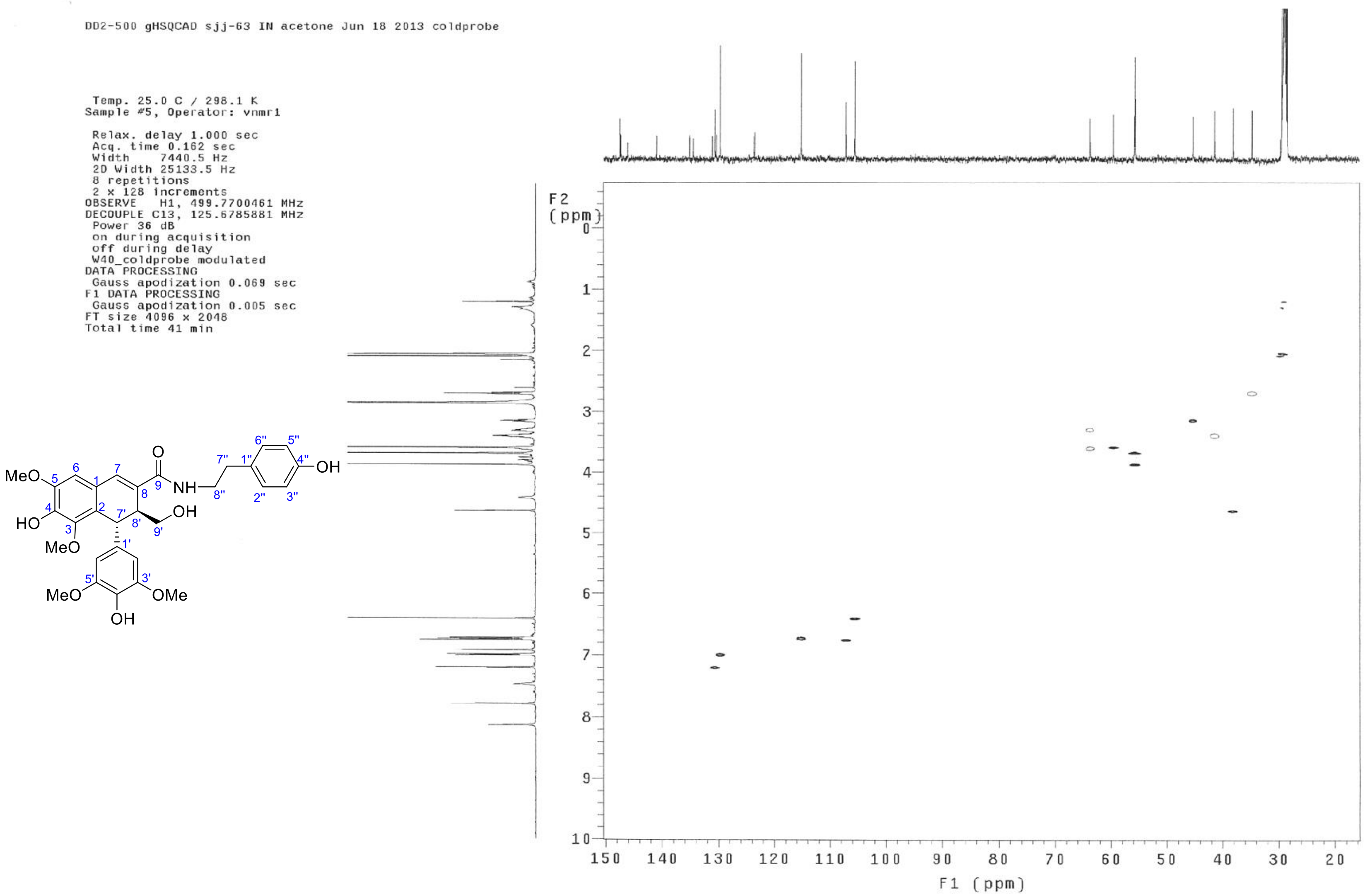

Figure S8. The HSQC Spectrum of Compound 1 in Acetone-d_6 (500 MHz)

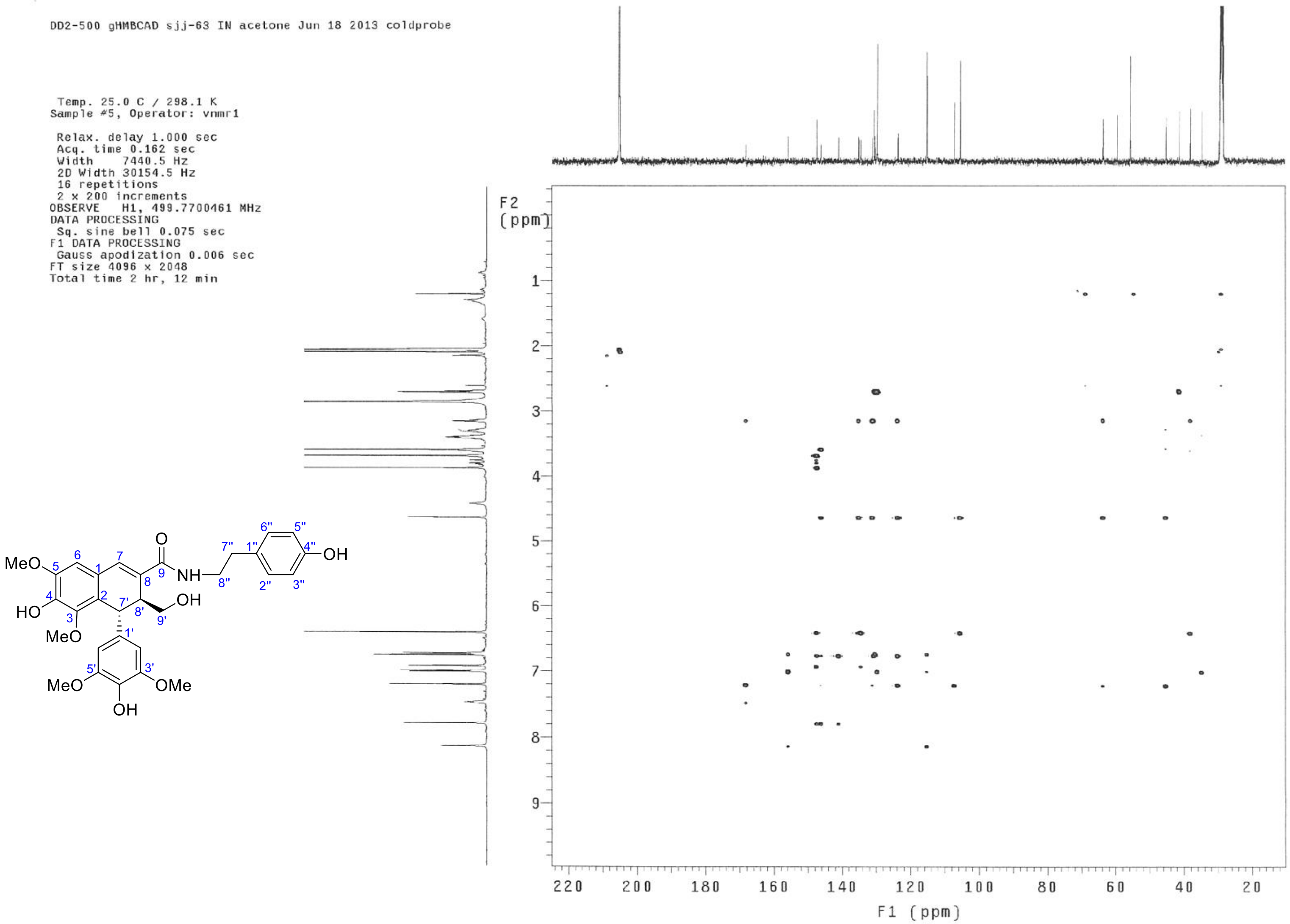

Figure S9. The HMBC Spectrum of Compound 1 in Acetone-d_6 (500 MHz)

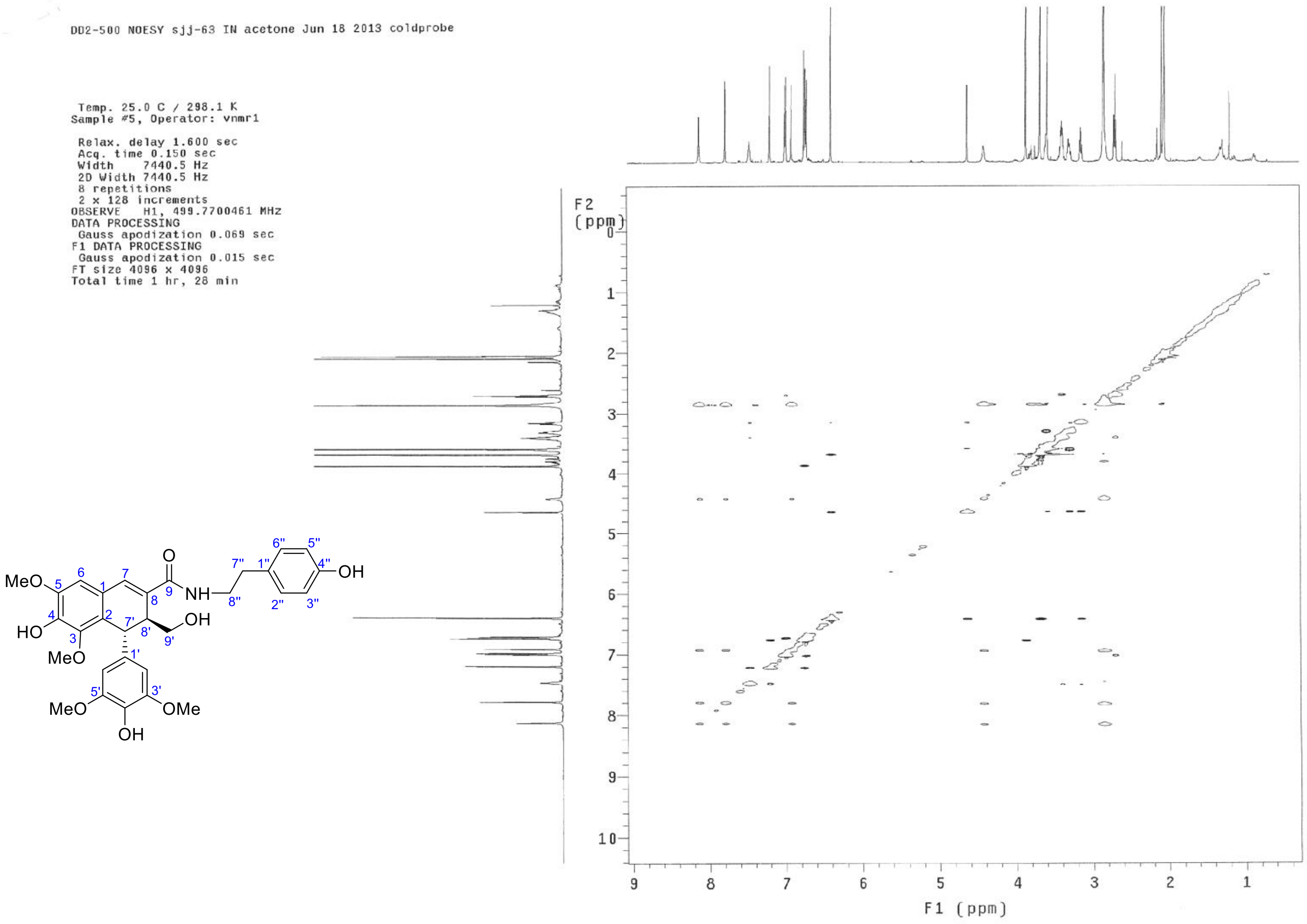

Figure S10. The NOESY Spectrum of Compound 1 in Acetone-d_6 (500 MHz)

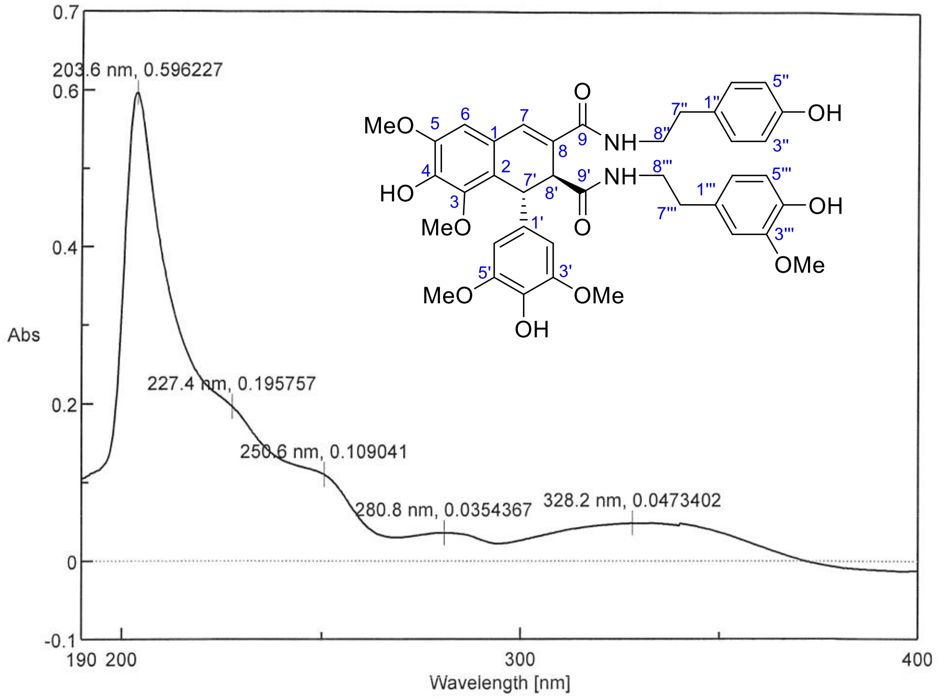

sjj-71a

[Comment]
Sample Name sjj-71a
Comment 0.02
User
Division UV
Company 324

[Measurement Information]
Instrument Name V-650
Model Name V-650
Serial No. A034461150

Accessory PSC-718
Accessory S/N A001761114
Position 1
Cell Length 10 mm
Temperature 19.97 C
Control Sonsor Holder
Monitor Sensor Holder
Start Mode Start immediately

Photometric Mode Abs
Measurement range 400 - 190 nm
Data pitch 0.2 nm
Band width(UV/Vis) 2.0 nm
Response Medium
Scanning speed 200 nm/min
Source Change 340 nm
Light Source D2/WI
Filter Exchange Step
Correction Baseline

[Data Information]
Creation Date 2014-11-18 19:56

Data array type Linear data array
Horizontal Wavelength [nm]
Vertical Abs
Start 400 nm
End 190 nm
Data pitch 0.2 nm
Data points 1051

Figure S11. The UV Spectra of Compound 2 in MeOH

Single Mass Spectrum Deconvolution Report

Analysis Name: linsh148.d
Method: def_lcms.m
Sample Name: sjj-71a
Analysis Info:

Instrument: LC-MSD-Trap-SL
Operator: Operator

Print Date: 10/27/2012 11:27:39 AM
Acq. Date: 10/27/2012 11:20:57 AM

Acquisition Parameter:

Mass Range Mode	Std/Normal	Trap Drive	29.0	Scan Begin	100 m/z
Ion Polarity	Positive	Octopole RF Amplitude	152.8 Vpp	Scan End	850 m/z
Ion Source Type	ESI	Capillary Exit	113.5 Volt	Averages	7 Spectra
Dry Temp (Set)	330 °C	Skimmer	40.0 Volt	Max. Accu Time	200000 µs
Nebulizer (Set)	15.00 psi	Oct 1 DC	12.00 Volt	ICC Target	30000
Dry Gas (Set)	5.00 l/min	Oct 2 DC	1.70 Volt	Charge Control	on

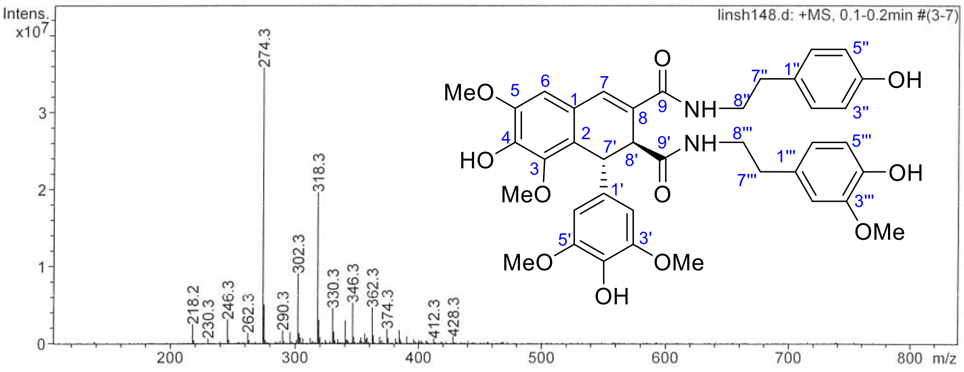

Component	Molecular Mass	Molecule	Absolute Abundance	Relative Abundance

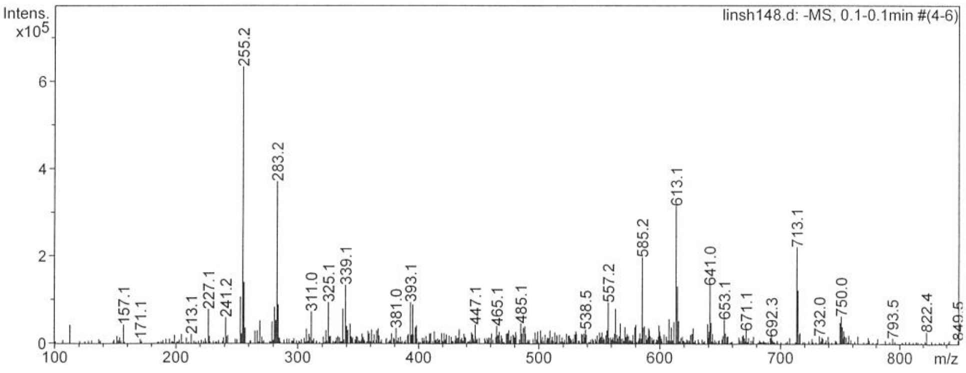

Component	Molecular Mass	Molecule	Absolute Abundance	Relative Abundance

MSD Trap Report v 4 (A4-Opt2) Page 1 of 1 Agilent Technologies

Figure S12. The ESI-Mass Spectrum of Compound 2 in MeOH

Qualitative Analysis Report

Fragmentor Voltage	Collision Energy	Ionization Mode
135	0	Esi

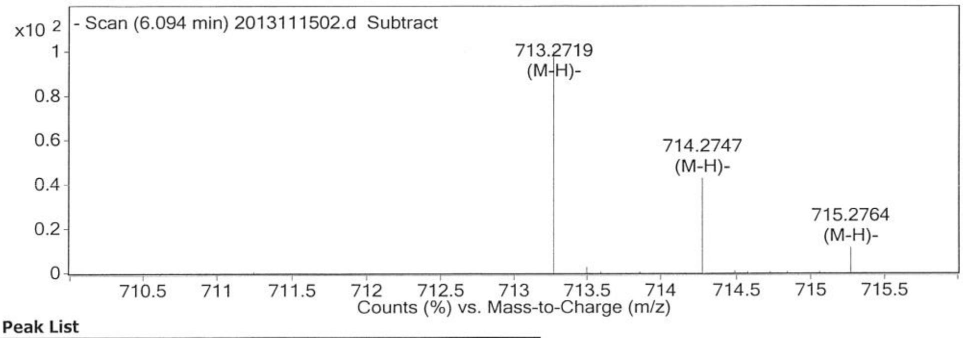

Peak List

m/z	z	Abund	Formula	Ion
112.9855		18090		
713.2719	1	264230	C39 H41 N2 O11	(M-H)-
714.2747	1	113435	C39 H41 N2 O11	(M-H)-
715.2764	1	29461	C39 H41 N2 O11	(M-H)-
781.2574	1	43558		
782.2614	1	19379		
803.24		15463		

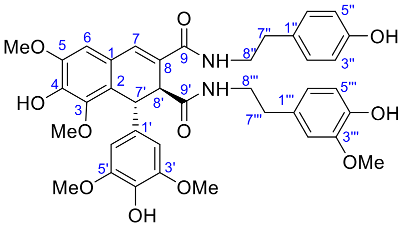

Formula Calculator Element Limits

Element	Min	Max
C	3	100
H	0	500
O	0	90
N	0	5
S	0	5
Cl	0	2
Br	0	0
Si	0	0
F	0	0
P	0	0

Formula Calculator Results

Formula	Best	Mass	Tgt Mass	Diff (ppm)	Ion Species	Score
C39 H42 N2 O11	TRUE	714.2791	714.2789	-0.39	C39 H41 N2 O11	99.97
C44 H42 O9		714.2791	714.2829	5.25	C44 H41 O9	99.16
C36 H46 N2 O11 S		714.2792	714.2822	4.31	C36 H45 N2 O11 S	99.03
C43 H42 N2 O6 S		714.2791	714.2764	-3.91	C43 H41 N2 O6 S	98.93
C31 H46 N4 O13 S		714.2792	714.2782	-1.34	C31 H45 N4 O13 S	98.78
C48 H42 O4 S		714.2791	714.2804	1.73	C48 H41 O4 S	98.47
C51 H38 O4		714.2791	714.277	-2.97	C51 H37 O4	98.2
C27 H46 N4 O18		714.2792	714.2807	2.18	C27 H45 N4 O18	98.09
C40 H46 N2 O6 S2		714.2792	714.2797	0.8	C40 H45 N2 O6 S2	98.02
C49 H38 N4 S		714.2792	714.2817	3.59	C49 H37 N4 S	97.95

--- End Of Report ---

Figure S13. The HR-Mass Spectrum of Compound 2 in MeOH

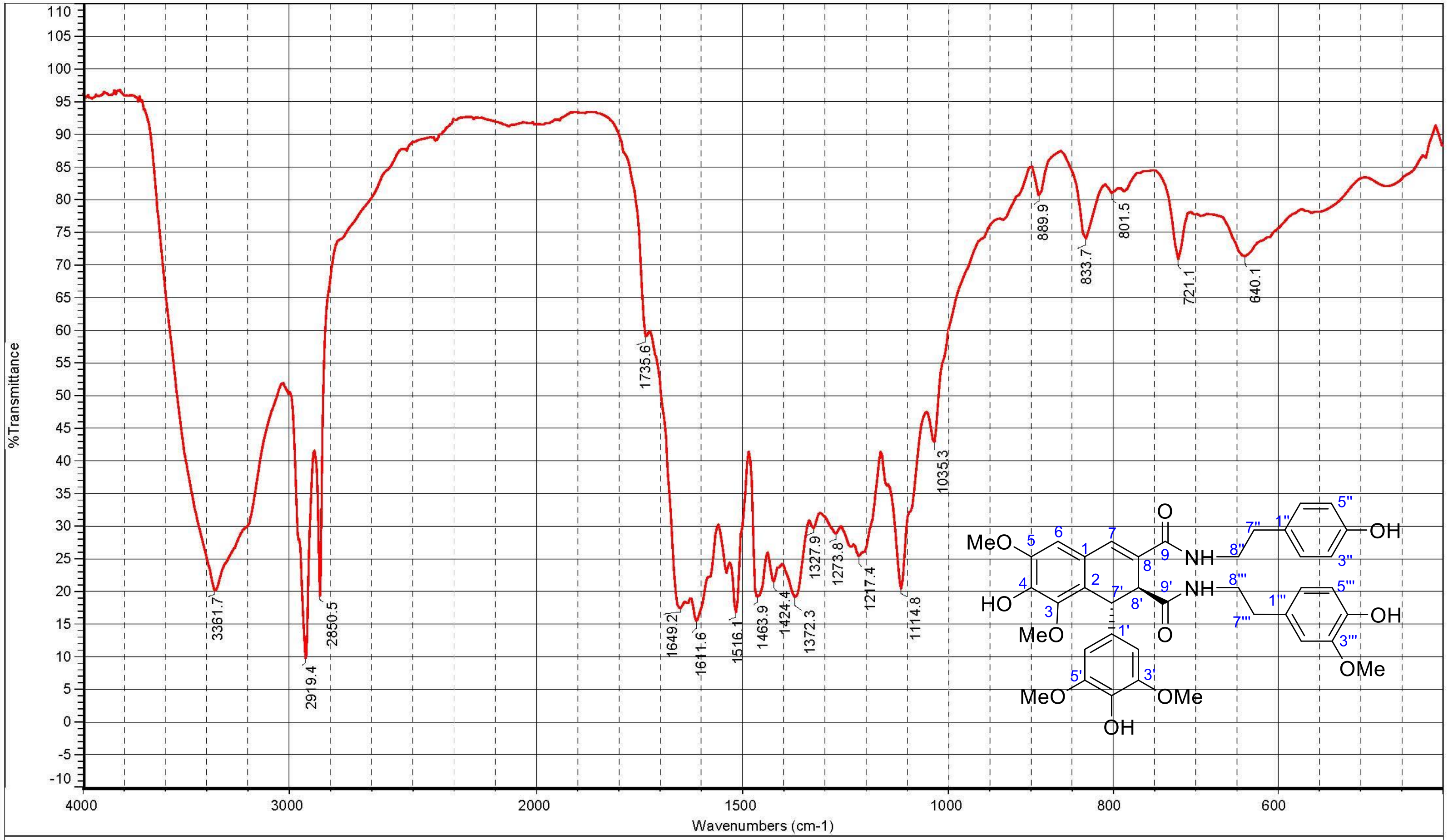

日期: 星期一 7月 14 13:24:30 2014 (GMT+08:00) Sample Name ： sjj - 71a (显微镜透射法FT- IR Microscope Transmission)

扫描次数: 100 傅里叶变换显微镜红外(FT-IR Microscope)：Centaurμs

分辨率: 8.000 美国热电公司(Thermo)傅里叶变换红外光谱仪:Nicolet 5700

Figure S14. The IR Spectrum of Compound 2

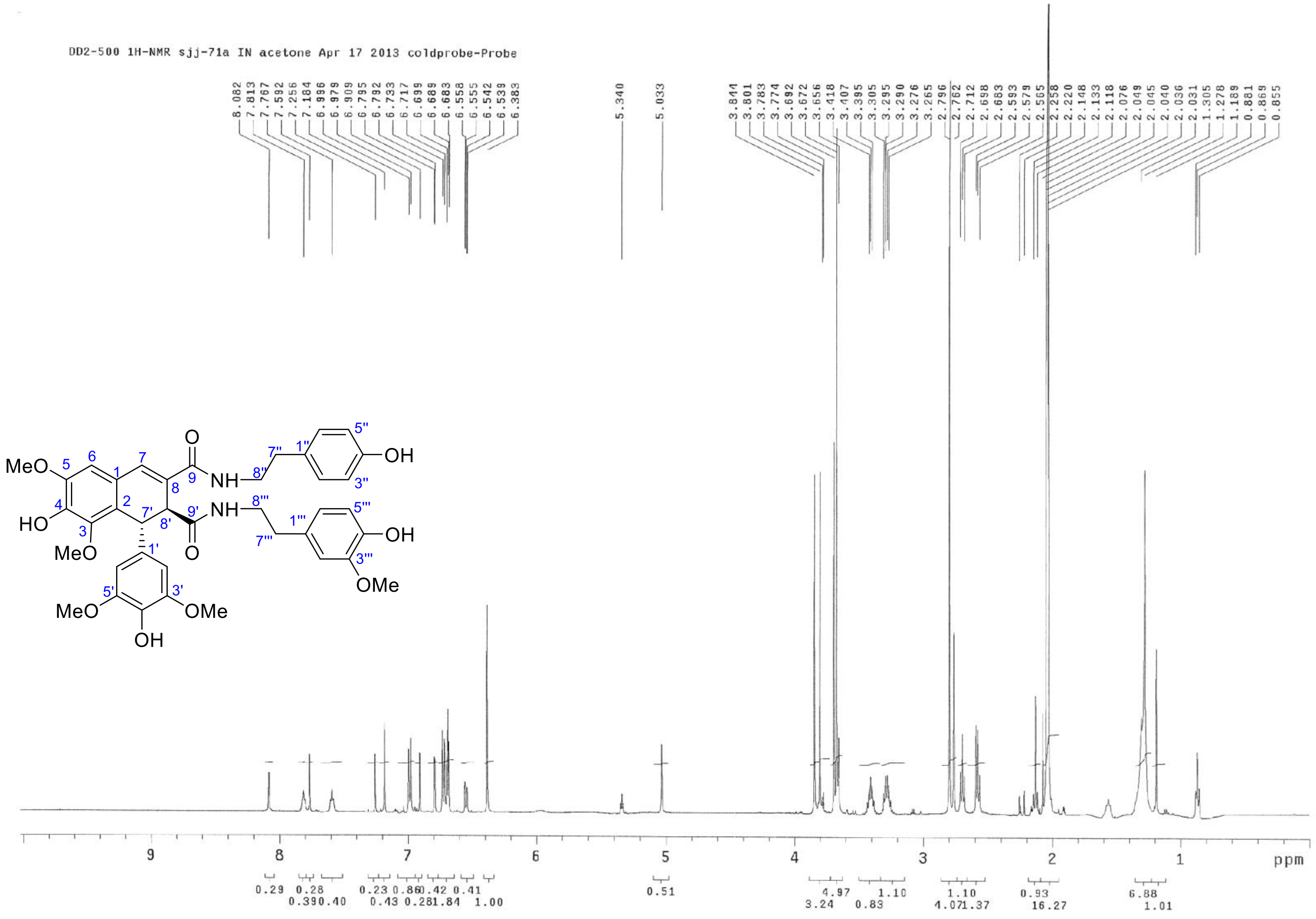

Figure S15. ^{1}**H NMR Spectrum of Compound 2 in Acetone-d_6 (500 MHz)**

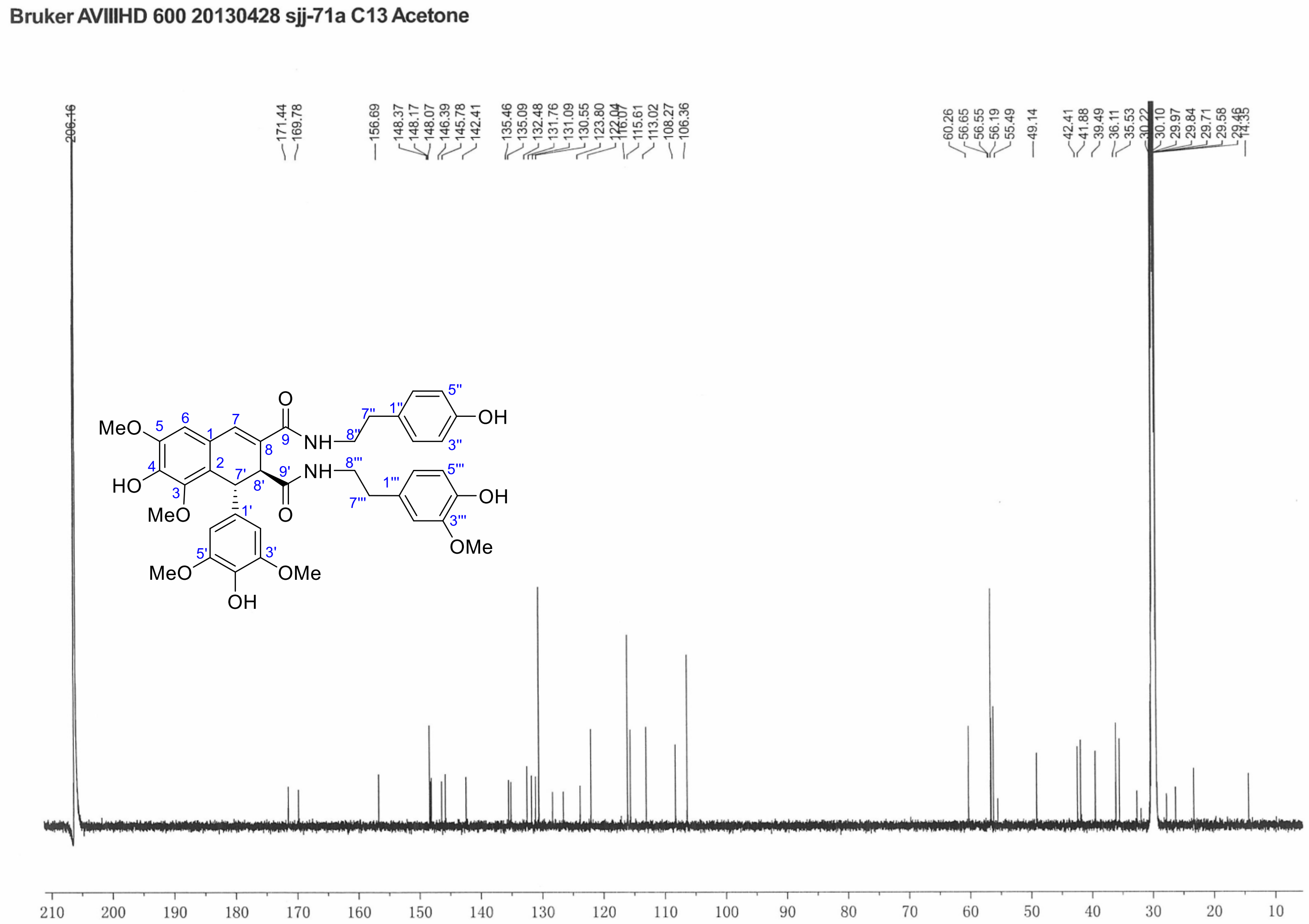

Figure S16. The [13] C NMR Spectrum of Compound 2 in Acetone-d_6 (150 MHz)

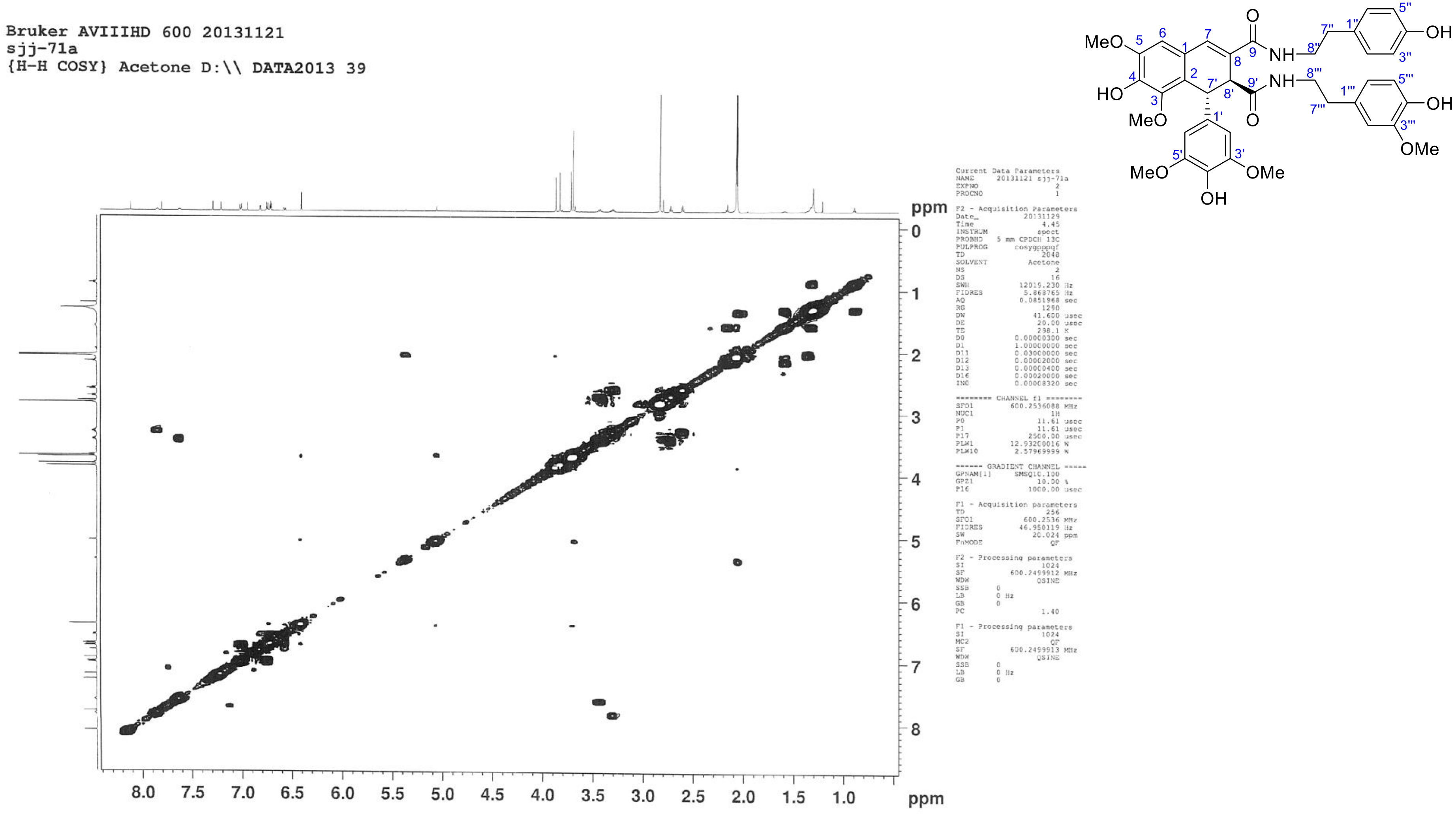

Figure S17. The ^{1}H-^{1}H COSY Spectrum of Compound 2 in Acetone-d_6 (600MHz)

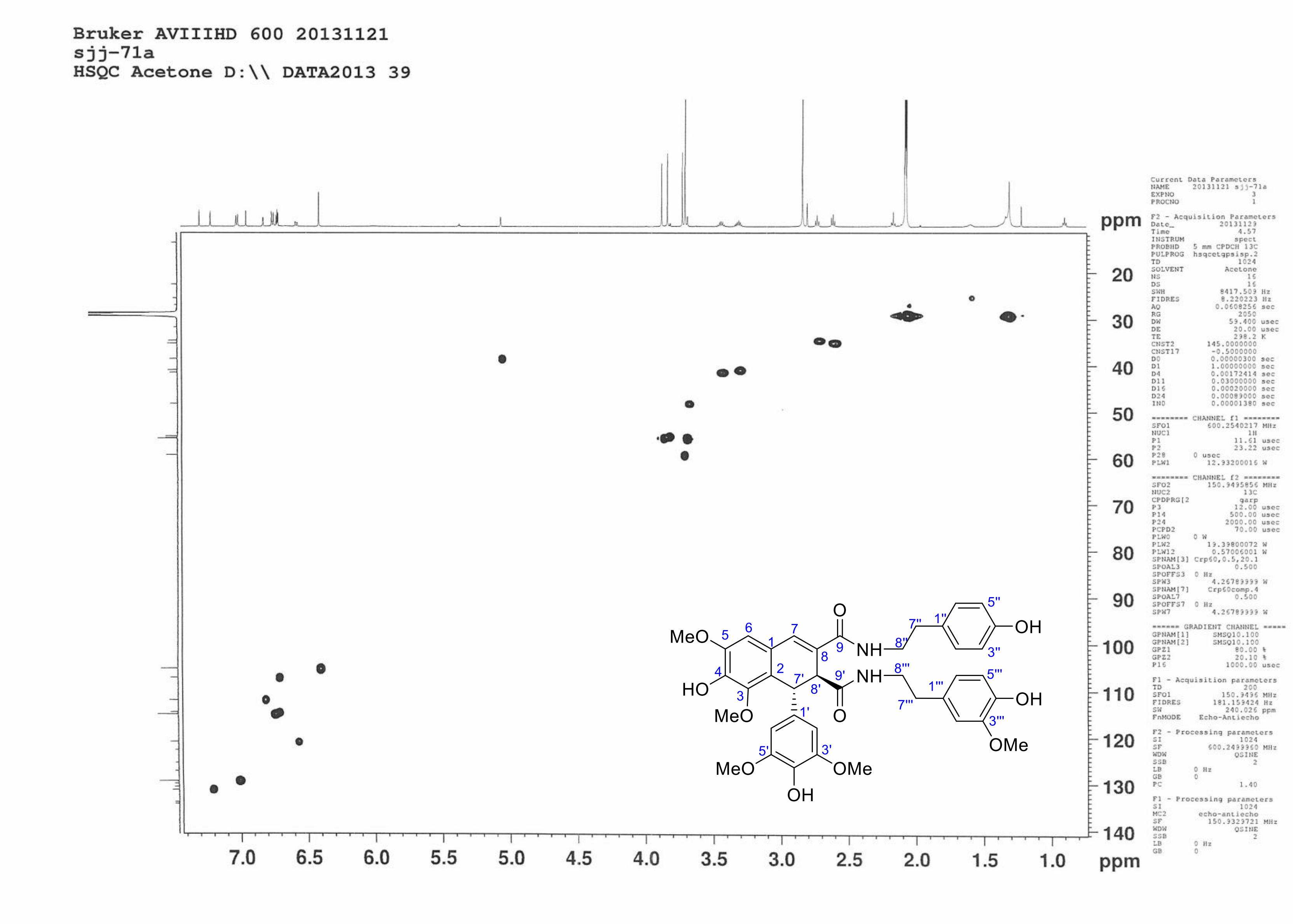

Figure S18. The HSQC Spectrum of Compound 2 in Acetone-d_6 (500 MHz)

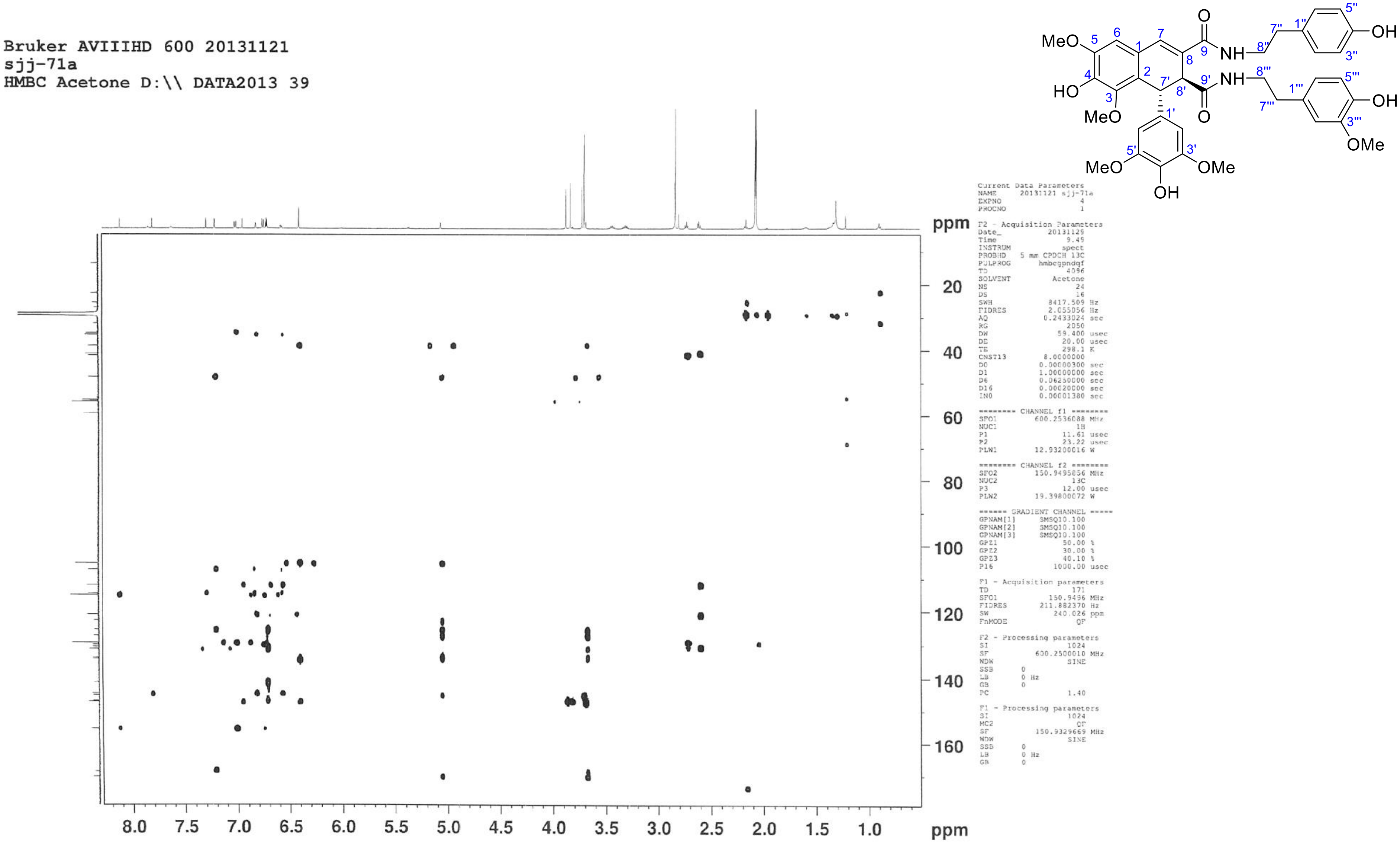

Figure S19. The HMBC Spectrum of Compound 2 in Acetone-d_6 (600MHz)

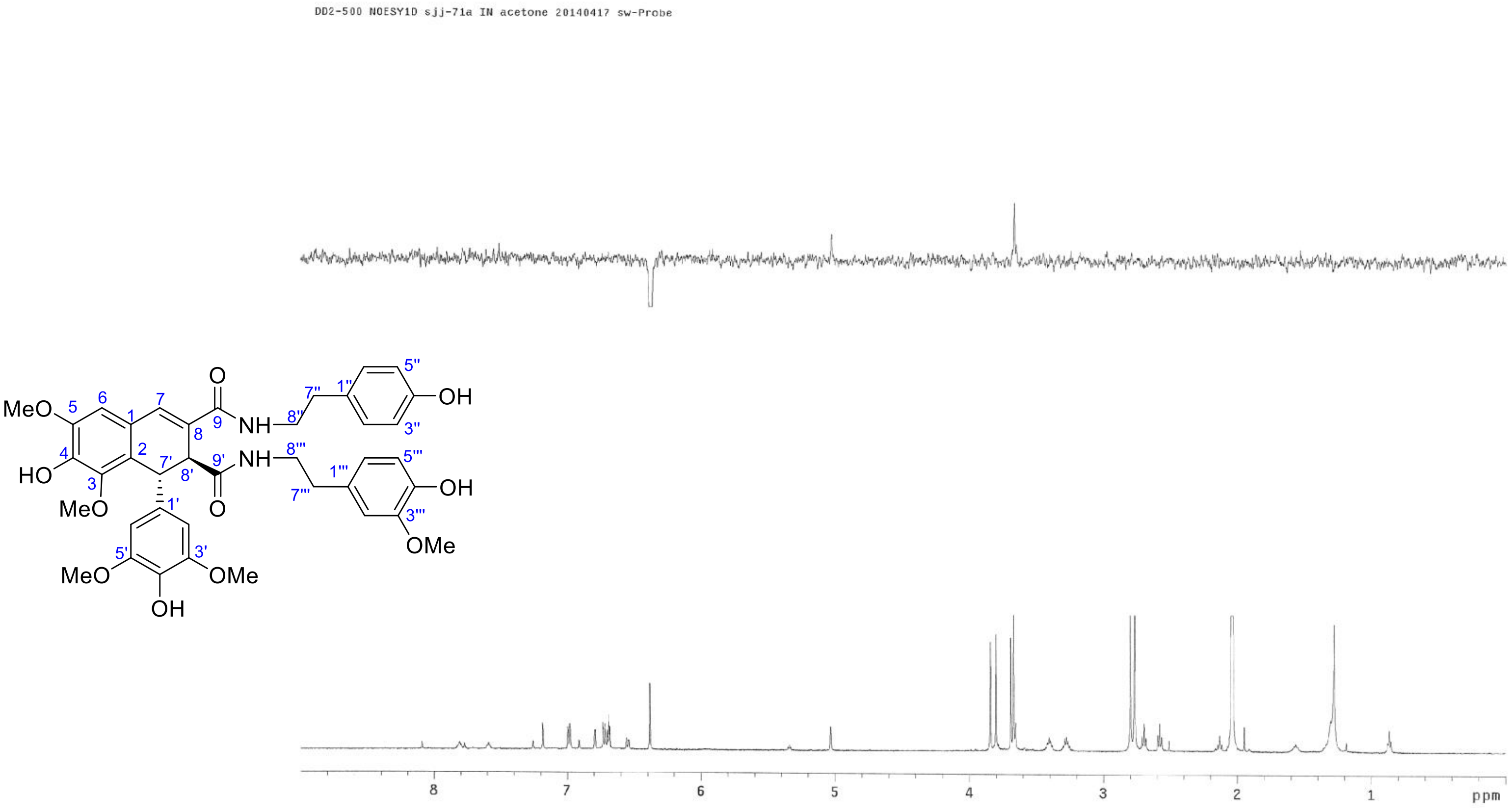

Figure S20. The 1D NOE Difference Spectrum of Compound 2 in Acetone-d_6 (500 MHz)

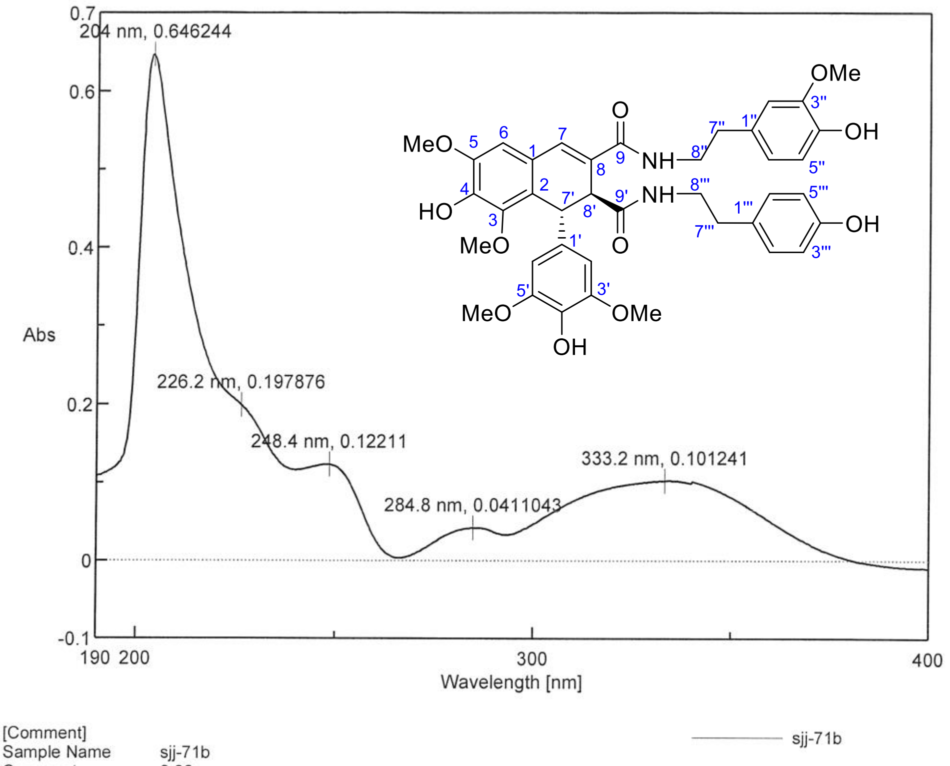

[Comment]
Sample Name sjj-71b
Comment 0.02
User
Division UV
Company 324
[Measurement Information]
Instrument Name V-650
Model Name V-650
Serial No. A034461150

Accessory PSC-718
Accessory S/N A001761114
Position 1
Cell Length 10 mm
Temperature 19.99 C
Control Sensor Holder
Monitor Sensor Holder
Start Mode Start immediately

Photometric Mode Abs
Measurement range 400 - 190 nm
Data pitch 0.2 nm
Band width(UV/Vis) 2.0 nm
Response Medium
Scanning speed 200 nm/min
Source Change 340 nm
Light Source D2/WI
Filter Exchange Step
Correction Baseline

[Data Information]
Creation Date 2014-11-18 20:48

Data array type Linear data array
Horizontal Wavelength [nm]
Vertical Abs
Start 400 nm
End 190 nm
Data pitch 0.2 nm
Data points 1051

Figure S21. The UV Spectra of Compound 3 in MeOH

Single Mass Spectrum Deconvolution Report

**Analysis Name:**	linsh147.d	**Instrument:**	LC-MSD-Trap-SL	**Print Date:**	10/27/2012 11:20:04 AM
**Method:**	def_lcms.m	**Operator:**	Operator	**Acq. Date:**	10/27/2012 11:18:01 AM
**Sample Name:**	sjj-71b				
**Analysis Info:**					

Acquisition Parameter:

Mass Range Mode	Std/Normal	Trap Drive	45.5	Scan Begin	100 m/z
Ion Polarity	Positive	Octopole RF Amplitude	152.8 Vpp	Scan End	800 m/z
Ion Source Type	ESI	Capillary Exit	-113.5 Volt	Averages	7 Spectra
Dry Temp (Set)	330 °C	Skimmer	-40.0 Volt	Max. Accu Time	200000 µs
Nebulizer (Set)	15.00 psi	Oct 1 DC	-12.00 Volt	ICC Target	10000
Dry Gas (Set)	5.00 l/min	Oct 2 DC	-1.70 Volt	Charge Control	on

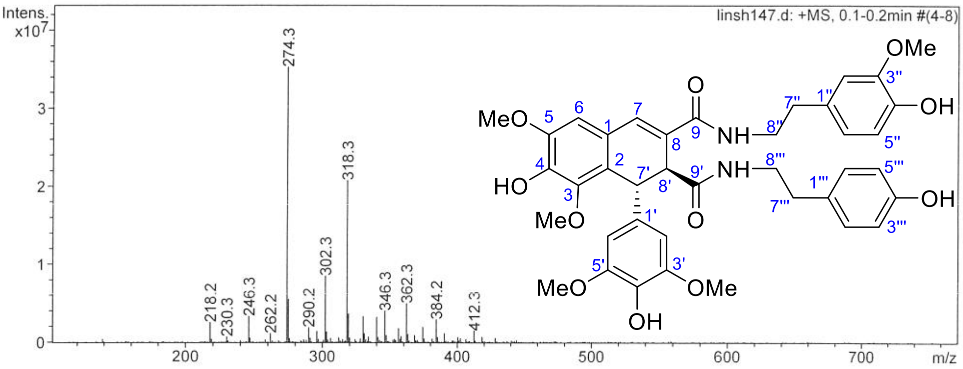

Component	Molecular Mass	Molecule	Absolute Abundance	Relative Abundance

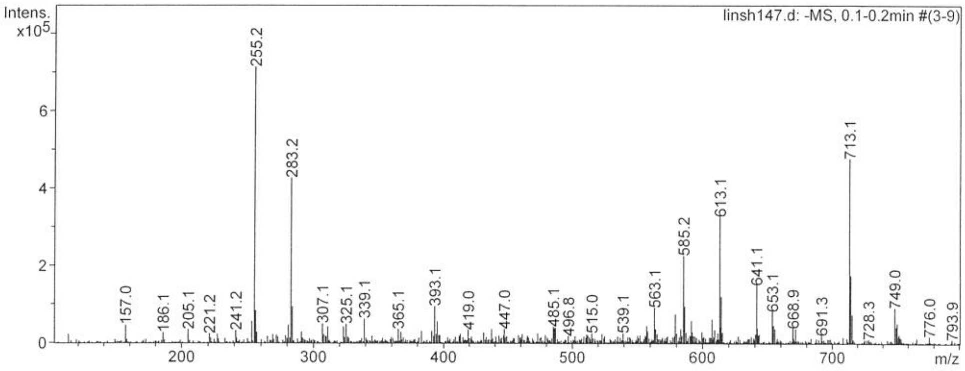

Component	Molecular Mass	Molecule	Absolute Abundance	Relative Abundance

MSD Trap Report v 4 (A4-Opt2) Page 1 of 1 Agilent Technologies

Figure S22. The ESI-Mass Spectrum of Compound 3 in MeOH

Qualitative Analysis Report

Fragmentor Voltage	Collision Energy	Ionization Mode
135	0	Esi

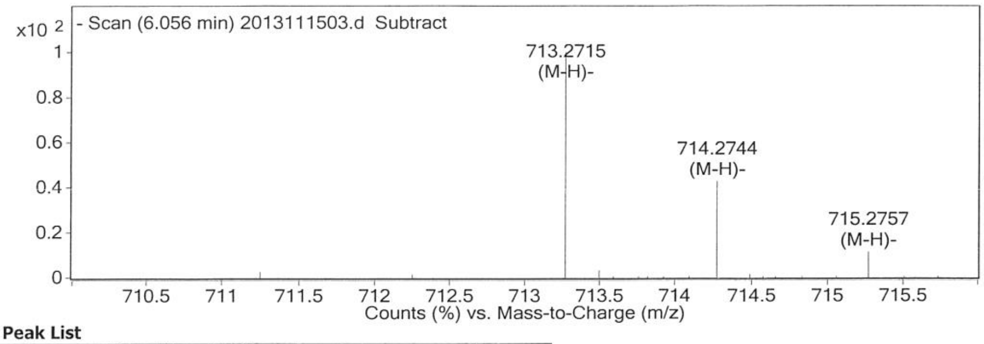

Peak List

m/z	z	Abund	Formula	Ion
713.2715	1	301634	C39 H41 N2 O11	(M-H)-
714.2744	1	129305	C39 H41 N2 O11	(M-H)-
715.2757	1	34080	C39 H41 N2 O11	(M-H)-
781.2575	1	49659		
782.2599	1	21336		

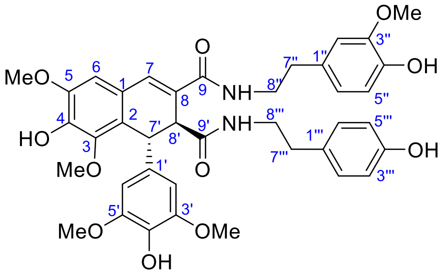

Formula Calculator Element Limits

Element	Min	Max
C	3	100
H	0	500
O	0	90
N	0	5
S	0	5
Cl	0	2
Br	0	0
Si	0	0
F	0	0
P	0	0

Formula Calculator Results

Formula	Best	Mass	Tgt Mass	Diff (ppm)	Ion Species	Score
C39 H42 N2 O11	TRUE	714.2787	714.2789	0.2	C39 H41 N2 O11	99.97
C34 H42 N4 O13		714.2787	714.2748	-5.45	C34 H41 N4 O13	99.3
C44 H42 O9		714.2787	714.2829	5.84	C44 H41 O9	99.04
C43 H42 N2 O6 S		714.2787	714.2764	-3.32	C43 H41 N2 O6 S	99.03
C36 H46 N2 O11 S		714.2787	714.2822	4.9	C36 H45 N2 O11 S	98.98
C31 H46 N4 O13 S		714.2787	714.2782	-0.74	C31 H45 N4 O13 S	98.84
C48 H42 O4 S		714.2787	714.2804	2.33	C48 H41 O4 S	98.45
C51 H38 O4		714.2787	714.277	-2.38	C51 H37 O4	98.25
C40 H46 N2 O6 S2		714.2787	714.2797	1.39	C40 H45 N2 O6 S2	98.06
C27 H46 N4 O18		714.2787	714.2807	2.77	C27 H45 N4 O18	98.04

--- End Of Report ---

Figure S23. The HR-Mass Spectrum of Compound 3 in MeOH

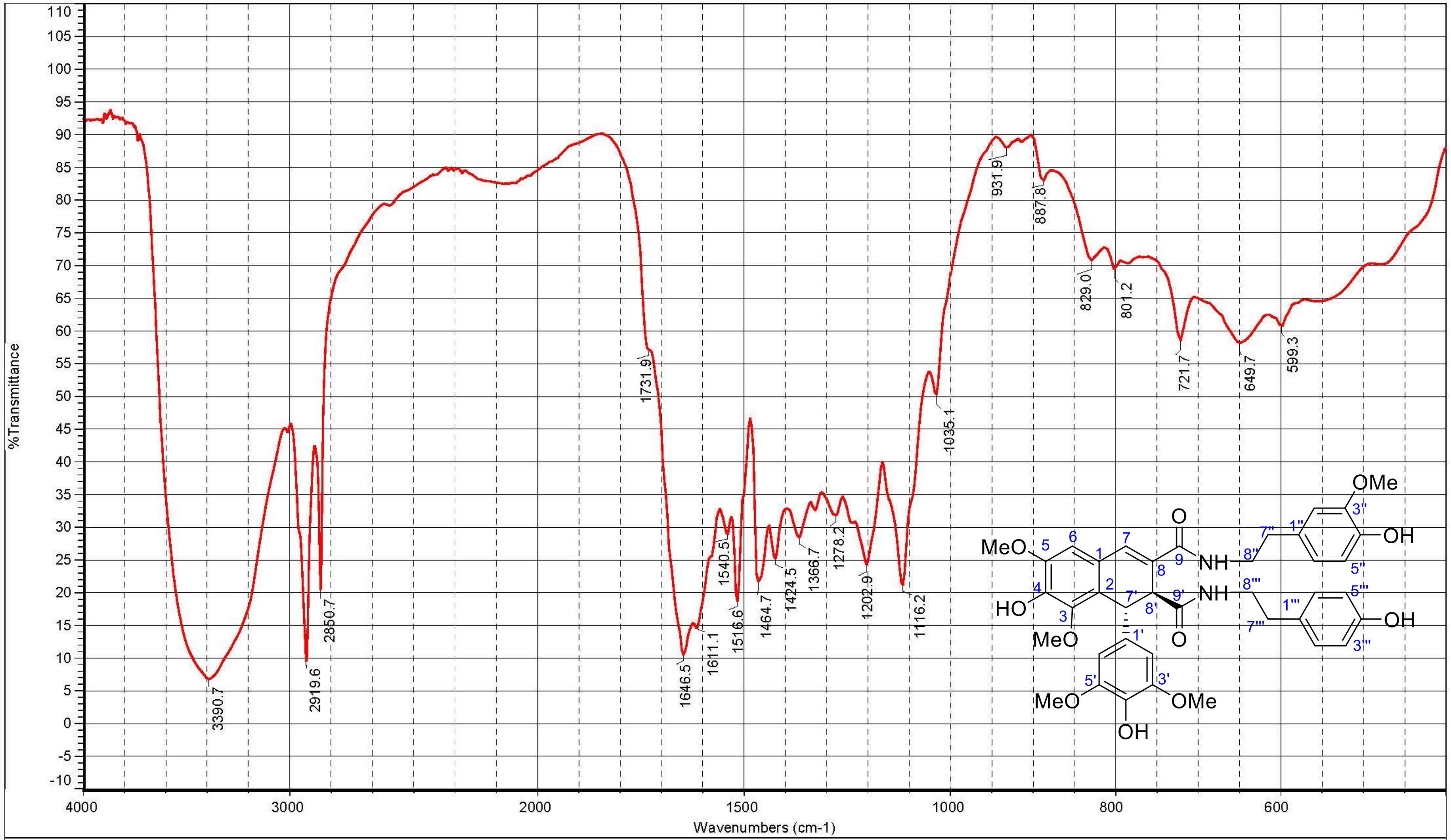

日期: 星期一 7月 14 09:28:22 2014 (GMT+08:00) Sample Name : sjj - 71b (显微镜透射法FT- IR Microscope Transmission)

扫描次数: 100 傅里叶变换显微镜红外(FT-IR Microscope)：Centaurμs

分辨率: 8.000 美国热电公司(Thermo)傅里叶变换红外光谱仪:Nicolet 5700

Figure S24. The IR Spectrum of Compound 3

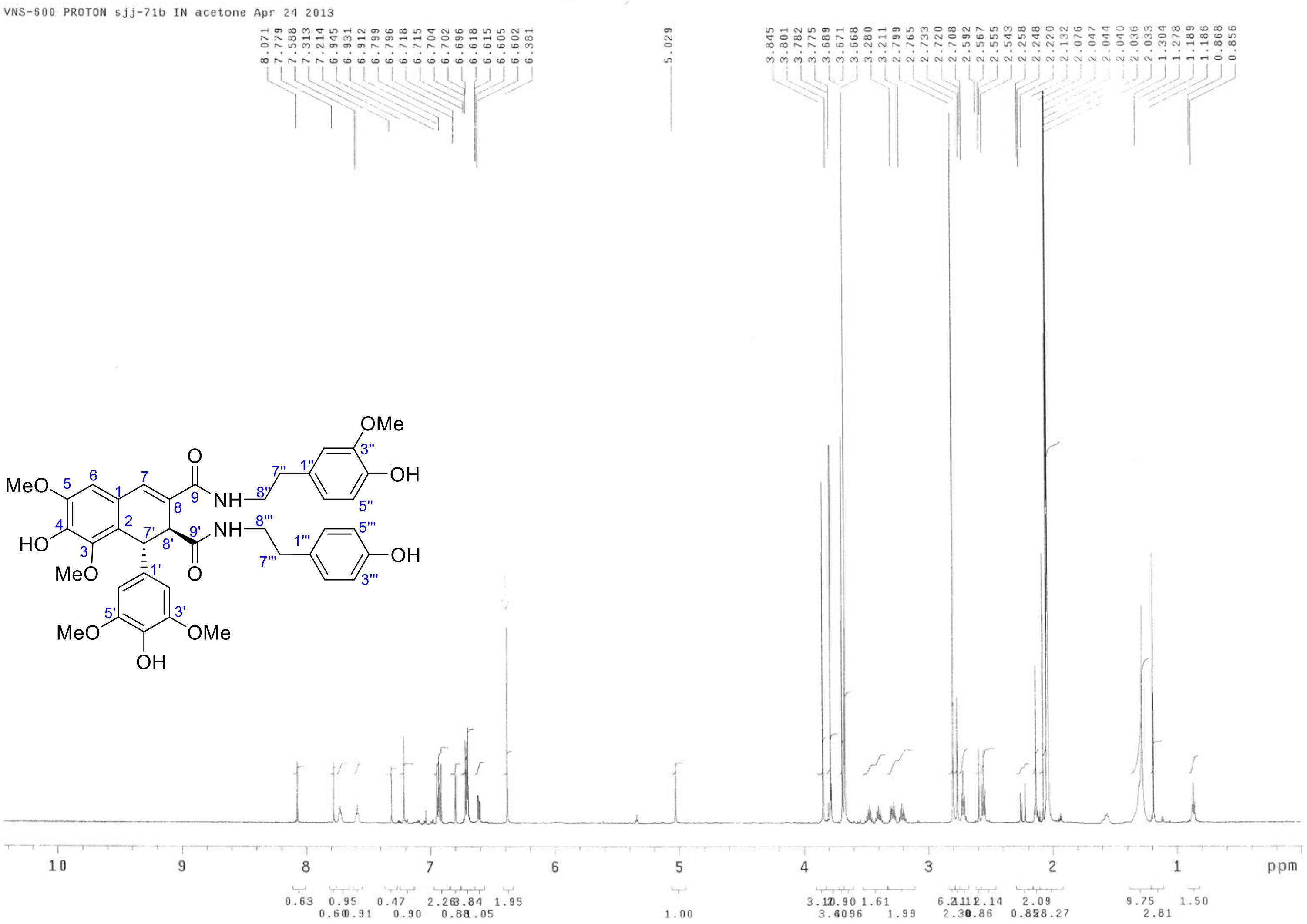

Figure S25. The ^{1}H NMR Spectrum of Compound 3 in Acetone-d_6 (600 MHz)

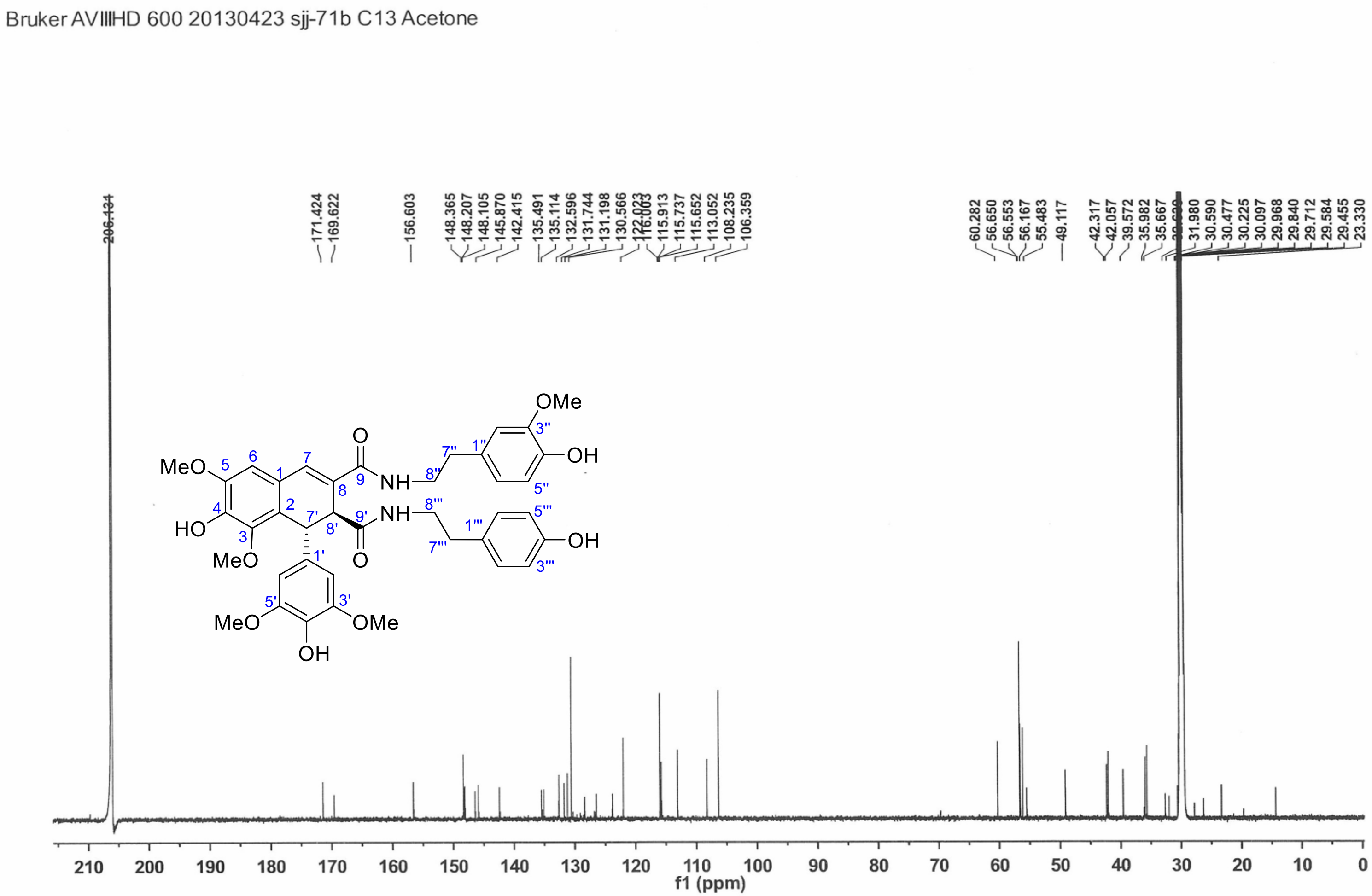

Figure S26. The ¹³ C NMR Spectrum of Compound 3 in Acetone-d_6 (150MHz)

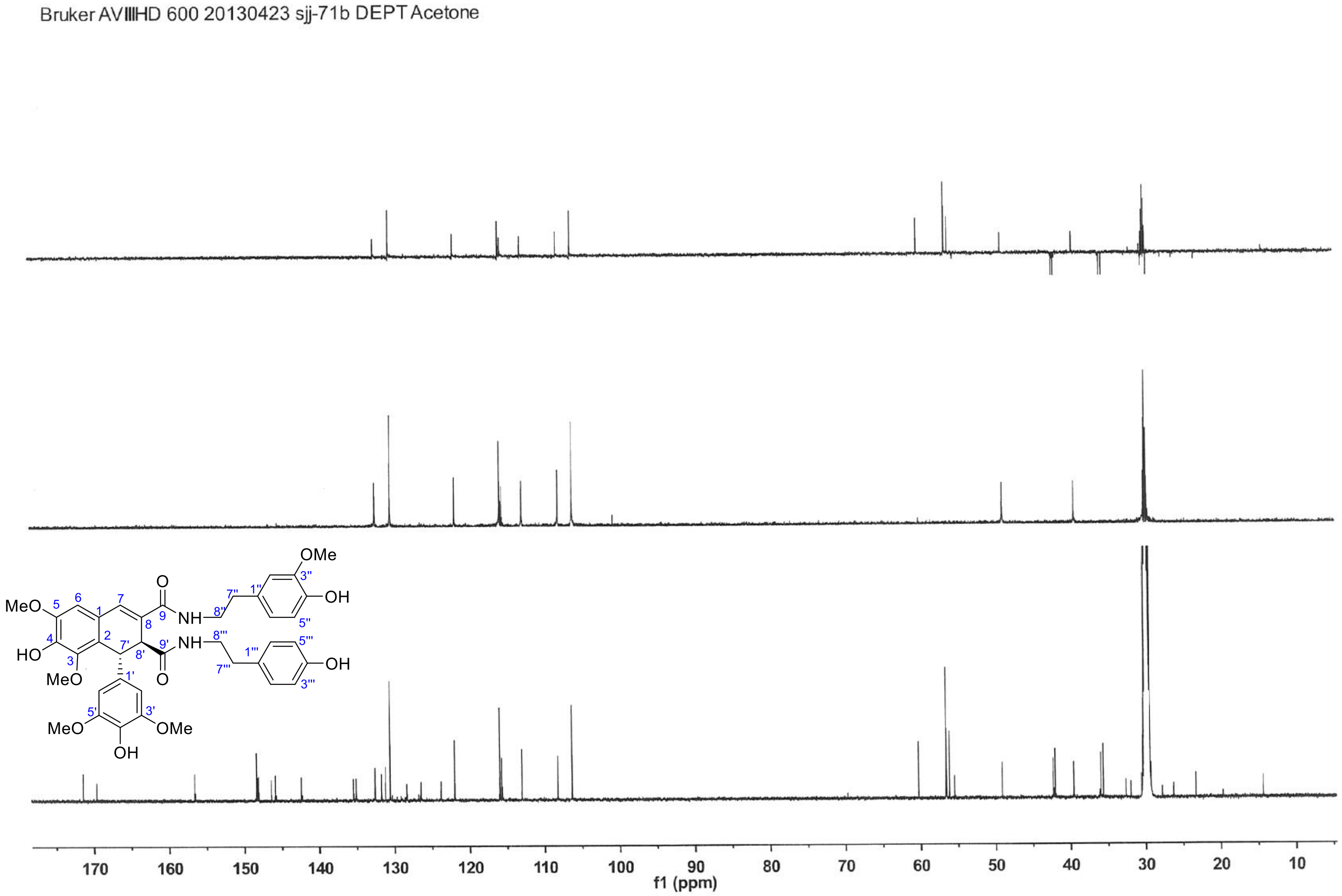

Figure S27. The DEPT Spectrum of Compound 3 in Acetone-d_6 (150MHz)

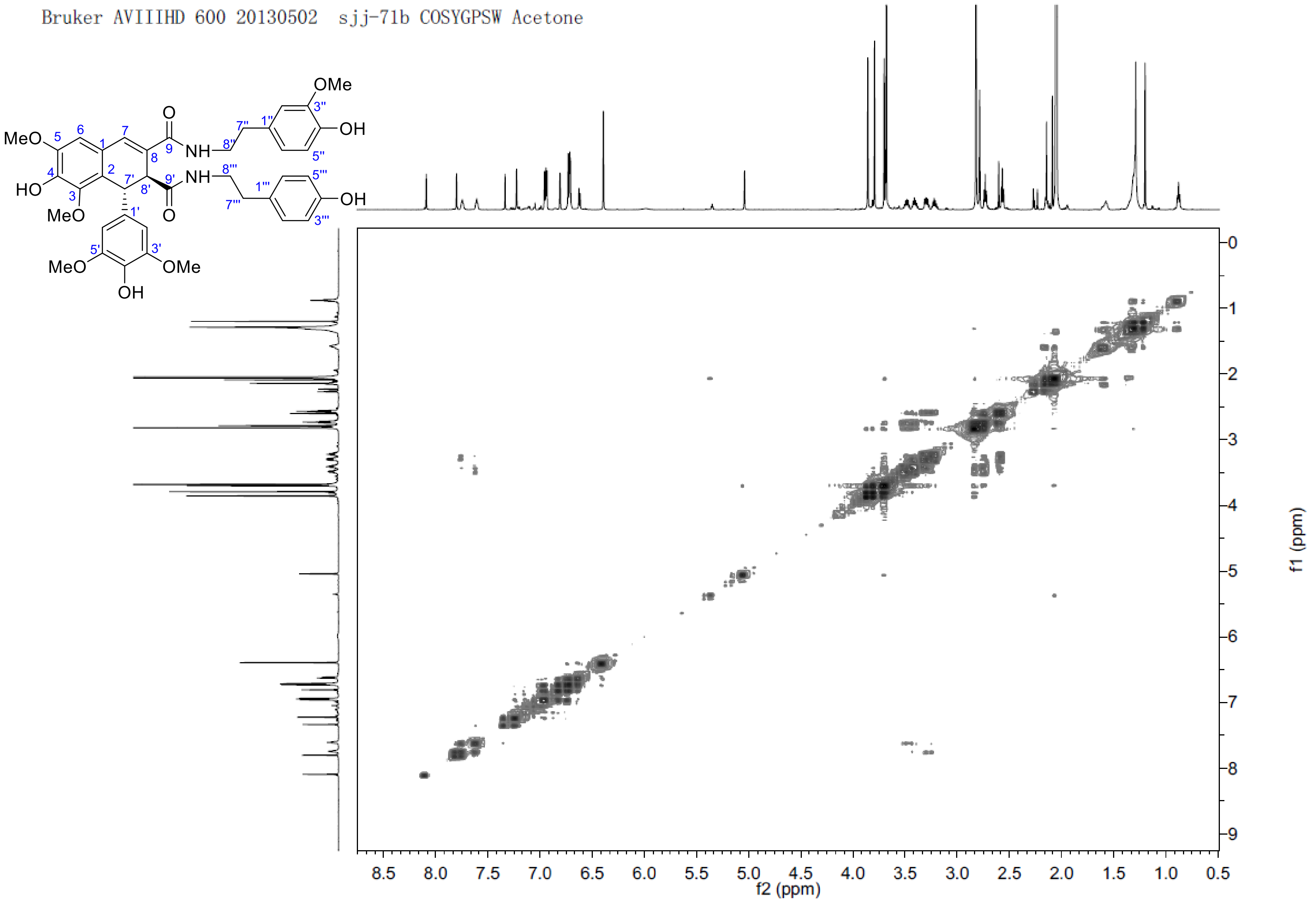

Figure 28. The ^{1}H-^{1}H COSY Spectrum of Compound 3 in Acetone-d_6 (600 MHz)

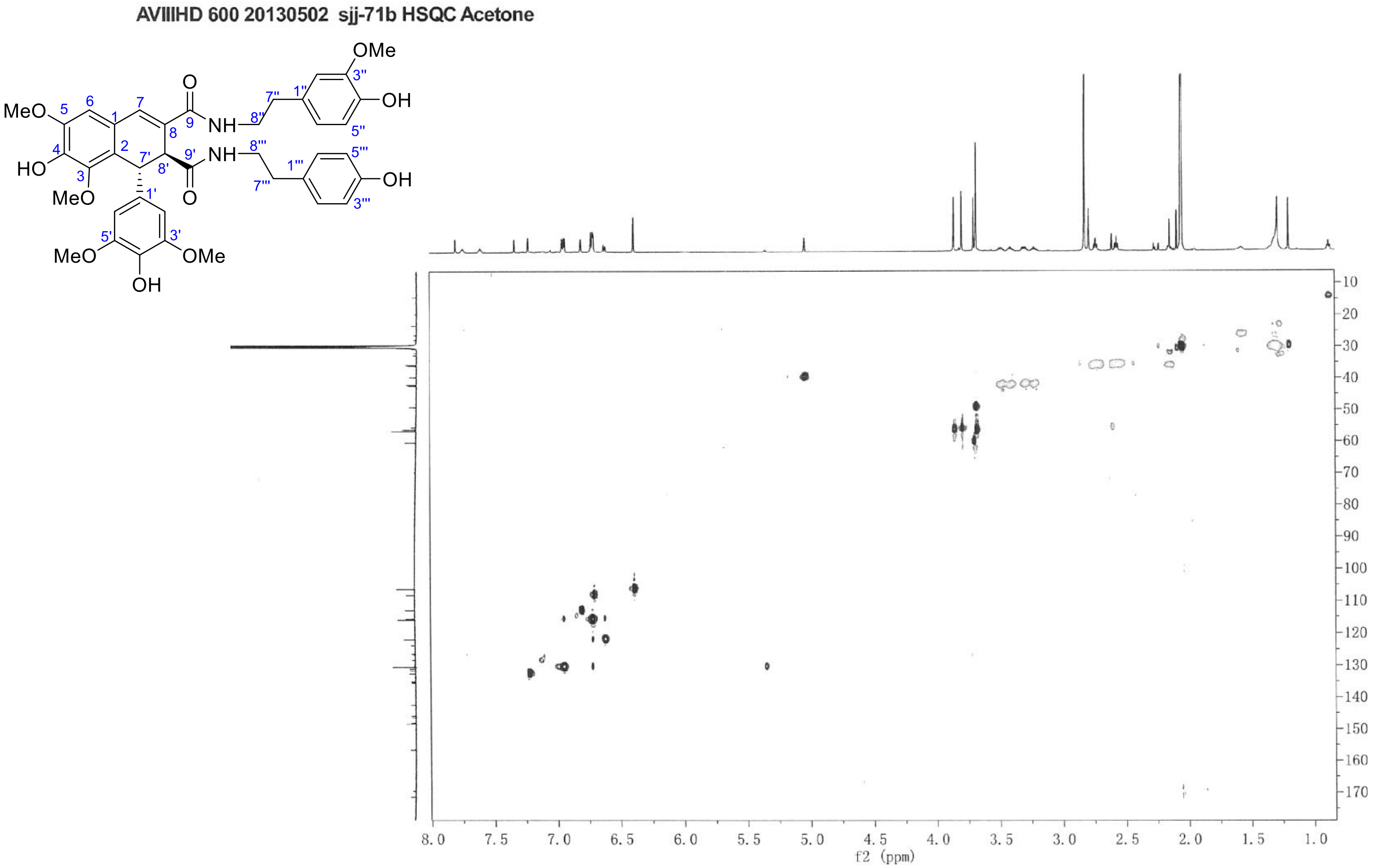

Figure 29. The HSQC Spectrum of Compound 3 in Acetone-d_6 (600 MHz)

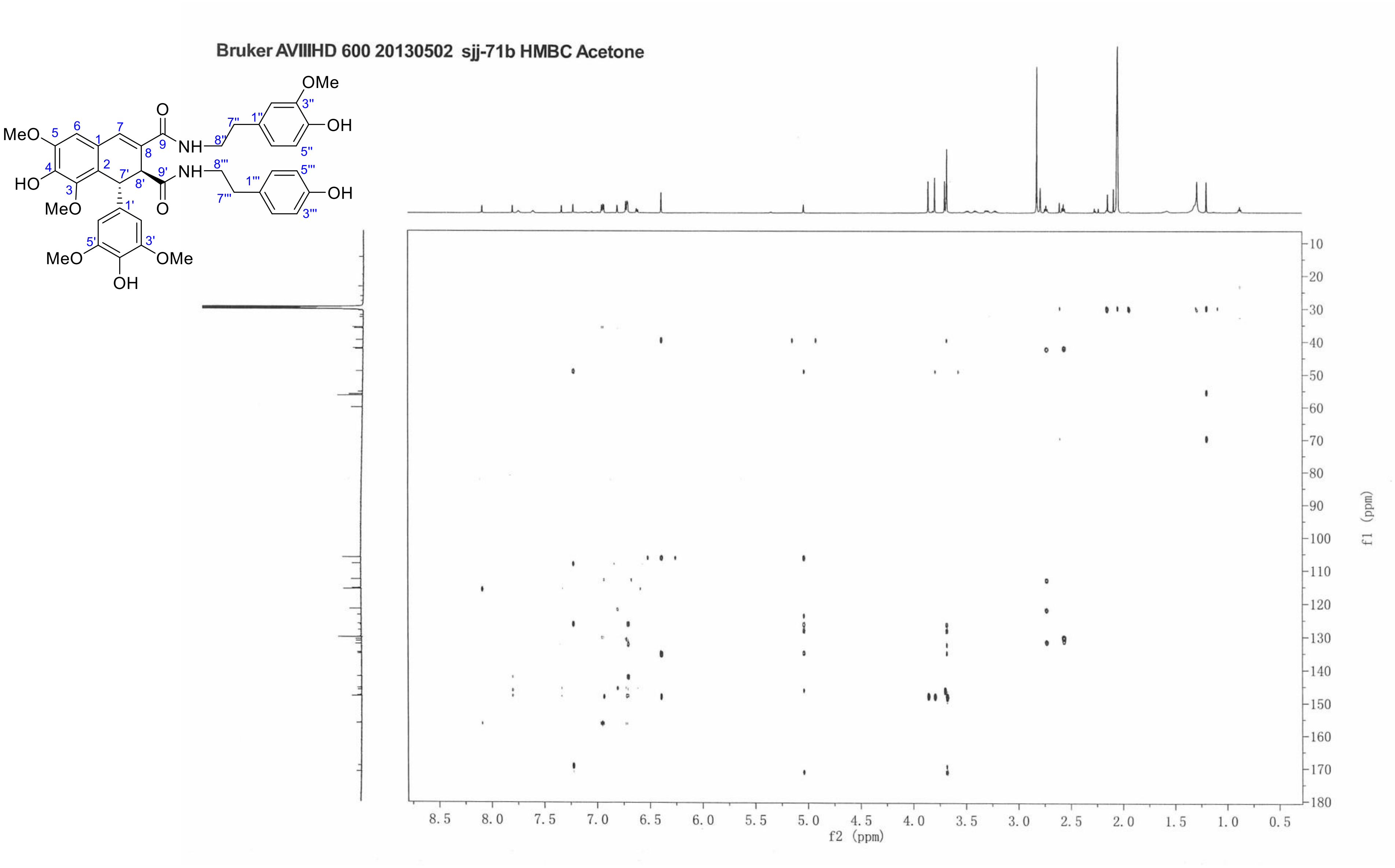

Figure 30. The HMBC Spectrum of Compound 3 in Acetone-d_6 (600 MHz)

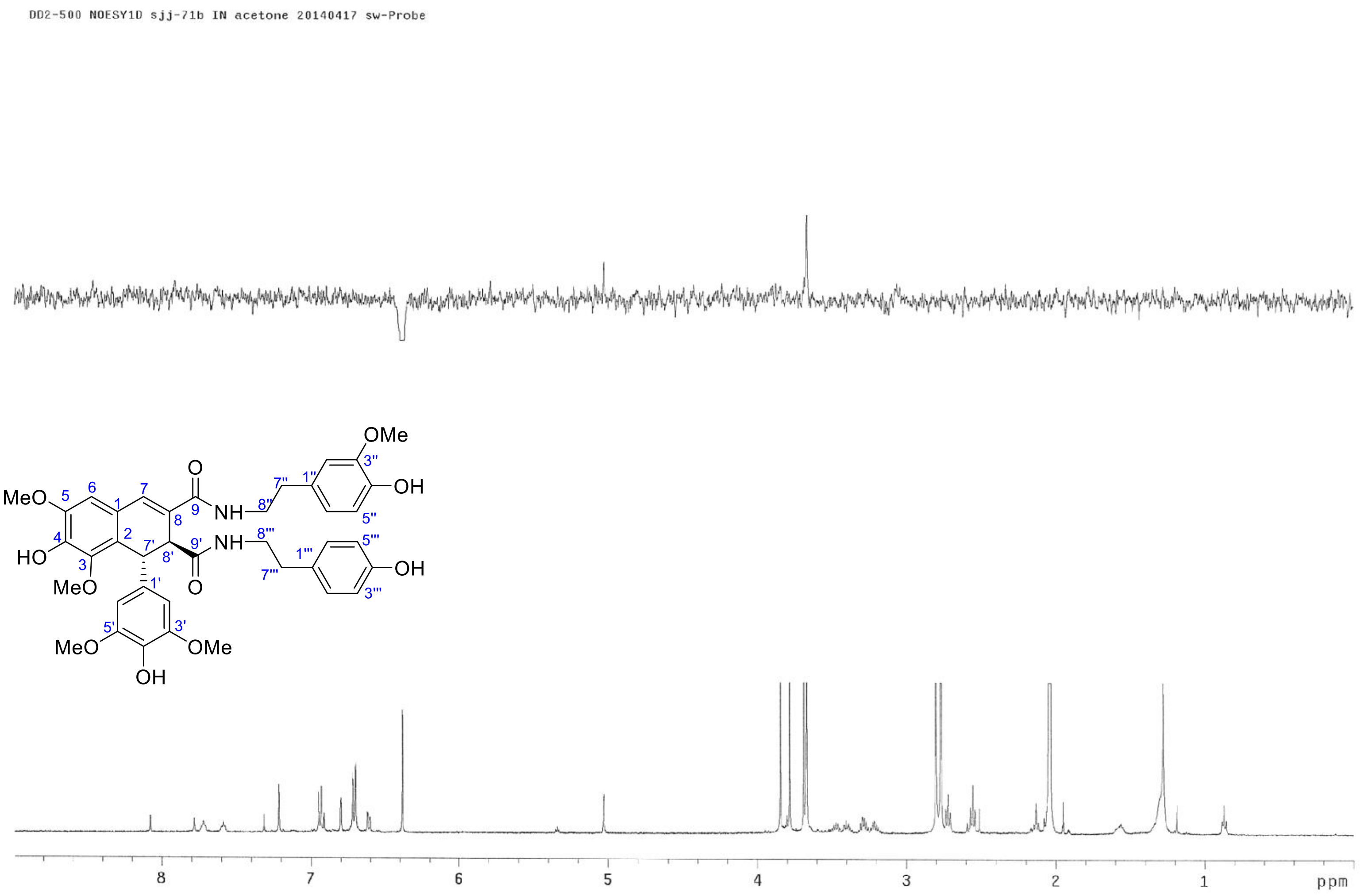

Figure S31. The 1D NOE Difference Spectrum of Compound 3 in Acetone-d_6 (500 MHz)

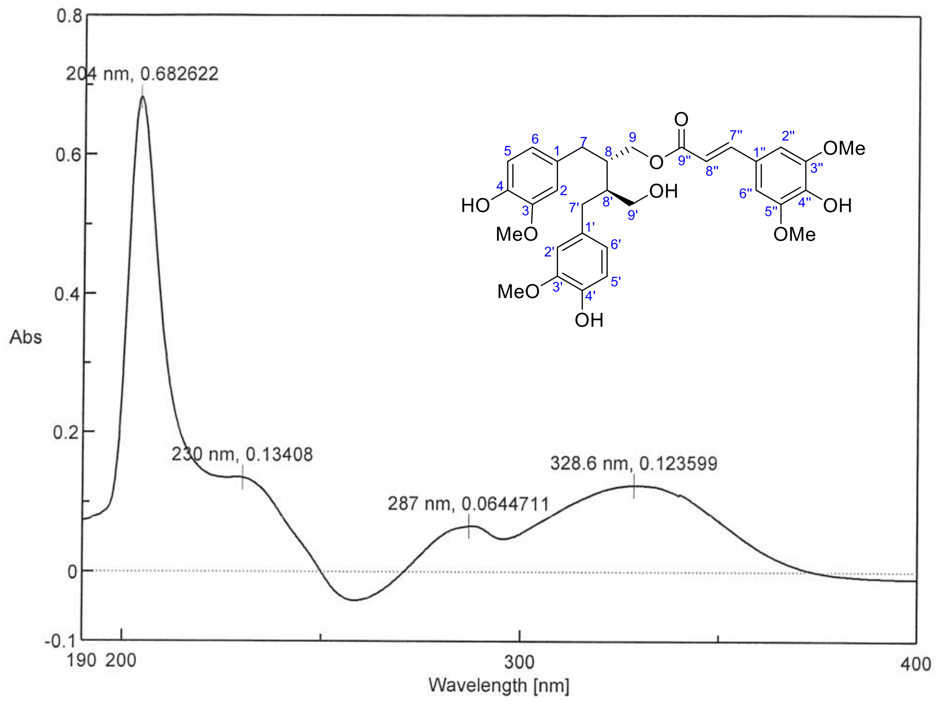

sjj-96

[Comment]
Sample Name sjj-96
Comment 0.02
User
Division UV
Company 324

[Measurement Information]
Instrument Name V-650
Model Name V-650
Serial No. A034461150

Accessory PSC-718
Accessory S/N A001761114
Position 1
Cell Length 10 mm
Temperature 19.99 C
Control Sonsor Holder
Monitor Sensor Holder
Start Mode Start immediately

Photometric Mode Abs
Measurement range 400 - 190 nm
Data pitch 0.2 nm
Band width(UV/Vis) 2.0 nm
Response Medium
Scanning speed 200 nm/min
Source Change 340 nm
Light Source D2/WI
Filter Exchange Step
Correction Baseline

[Data Information]
Creation Date 2014-11-18 20:36

Data array type Linear data array
Horizontal Wavelength [nm]
Vertical Abs
Start 400 nm
End 190 nm
Data pitch 0.2 nm
Data points 1051

Figure S32. The UV Spectra of Compound 4 in MeOH

Single Mass Spectrum Deconvolution Report

Analysis Name: linsh144.d **Instrument:** LC-MSD-Trap-SL **Print Date:** 10/27/2012 11:17:21 AM
Method: def_lcms.m **Operator:** Operator **Acq. Date:** 10/27/2012 11:09:44 AM
Sample Name: sjj-96
Analysis Info:

Acquisition Parameter:

Mass Range Mode	Std/Normal	Trap Drive	29.0	Scan Begin	100 m/z
Ion Polarity	Positive	Octopole RF Amplitude	152.8 Vpp	Scan End	700 m/z
Ion Source Type	ESI	Capillary Exit	113.5 Volt	Averages	7 Spectra
Dry Temp (Set)	330 °C	Skimmer	40.0 Volt	Max. Accu Time	200000 µs
Nebulizer (Set)	15.00 psi	Oct 1 DC	12.00 Volt	ICC Target	10000
Dry Gas (Set)	5.00 l/min	Oct 2 DC	1.70 Volt	Charge Control	on

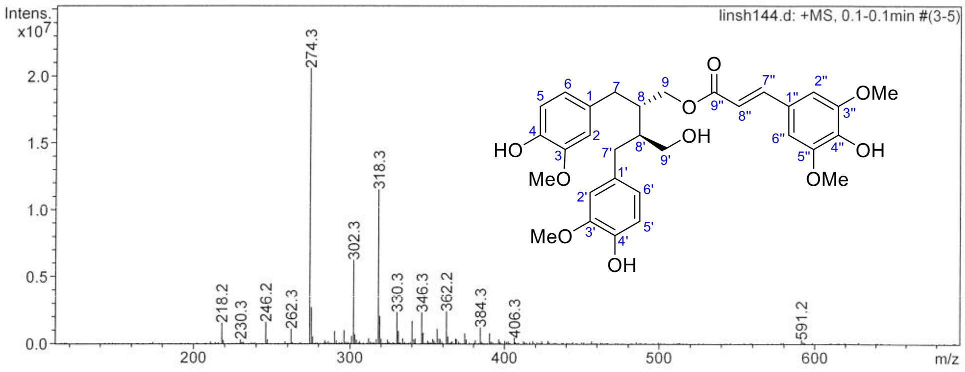

Component	Molecular Mass	Molecule	Absolute Abundance	Relative Abundance

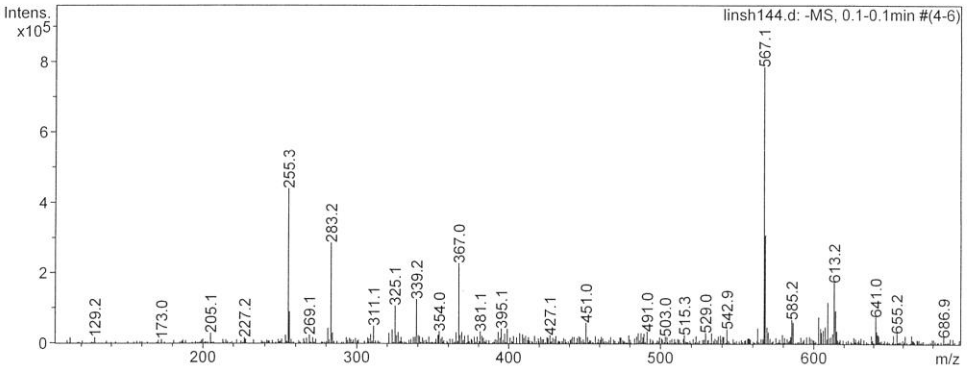

Component	Molecular Mass	Molecule	Absolute Abundance	Relative Abundance

MSD Trap Report v 4 (A4-Opt2) Page 1 of 1 Agilent Technologies

Figure S33. The ESI-Mass Spectrum of Compound 4 in MeOH

Qualitative Analysis Report

**Data Filename**	2013112202.d	**Sample Name**	sjj-96
**Sample Type**	Sample	**Position**	P1-C2
**Instrument Name**	Instrument 1	**User Name**	
**Acq Method**		**IRM Calibration Status**	Success
**DA Method**	TEST LCMS.m	**Comment**	

User Chromatograms

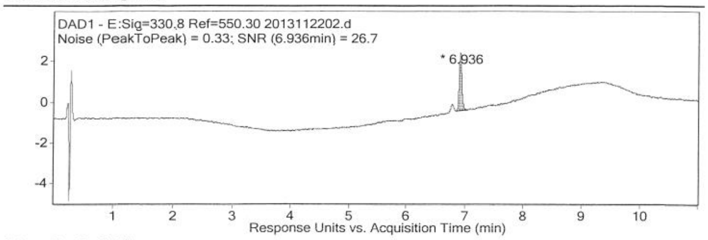

Integration Peak List

Peak	Start	RT	End	Height	Area	Area %	Signal To Noise
1	6.842	6.936	7.043	2.83	8.89	100	26.7

Noise Measurements

Noise Type	Signal Definition	Noise Multiplier	Noise Value
Peak-to-Peak	Area	1	0.333309174

Noise Regions

Start	End
0.5	1
5	5.3
9.99	11

Fragmentor Voltage 135 **Collision Energy** 0 **Ionization Mode** ESI

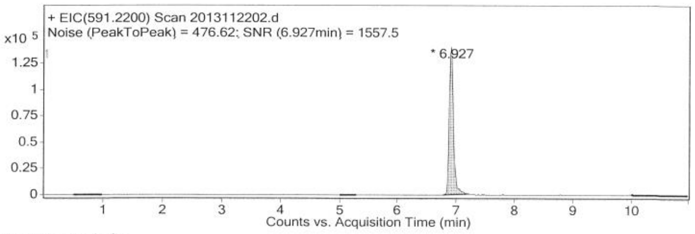

Integration Peak List

Peak	Start	RT	End	Height	Area	Area %	Signal To Noise
1	6.782	6.927	7.233	140373	742339	100	1557.5

Noise Measurements

Noise Type	Signal Definition	Noise Multiplier	Noise Value
Peak-to-Peak	Area	1	476.6247253

Noise Regions

Start	End
0.5	1
5	5.3
9.99	11

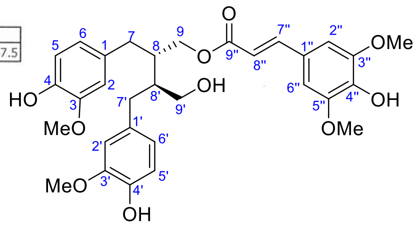

User Spectra

MS Formula Results: + Scan (6.927 min) Sub (2013112202.d)

m/z	Ion	Formula	Abundance
569.2387	(M+H)+	C31 H37 O10	18770.7

Best	Formula (M)	Ion Formula	Calc m/z	Score	Cross S	Mass	Calc Mass	Diff (ppm)	Abs Diff (ppm)	Abund Match	Spacing Mat	Mass Match	m/z	DBE
✓	C31 H36 O10	C31 H37 O10	569.2381	99.95		568.2314	568.2308	-0.98	0.98	99.87	100	99.97	569.2387	14
	C32 H32 N4 O6	C32 H33 N4 O6	569.2395	99.73		568.2314	568.2322	1.36	1.36	99.18	99.99	99.94	569.2387	19
	C28 H40 O10 S	C28 H41 O10 S	569.2415	99.01		568.2314	568.2342	4.94	4.94	98.29	99.59	99.15	569.2387	9
	C35 H36 O5 S	C35 H37 O5 S	569.2356	98.79		568.2314	568.2283	-5.39	5.39	97.65	99.73	98.99	569.2387	18
	C36 H32 N4 O S	C36 H33 N4 O S	569.237	98.78		568.2314	568.2297	-3.05	3.05	96.53	99.7	99.68	569.2387	23
	C23 H40 N2 O12 S	C23 H41 N2 O12 S	569.2375	98.46		568.2314	568.2302	-2.15	2.15	95.36	99.44	99.84	569.2387	5
	C32 H40 O5 S2	C32 H41 O5 S2	569.239	98.19		568.2314	568.2317	0.53	0.53	94.32	99.23	99.99	569.2387	13
	C33 H36 N4 O S2	C33 H37 N4 O S2	569.2403	98.08		568.2314	568.2331	2.87	2.87	94.44	99.19	99.71	569.2387	18
	C19 H40 N2 O17	C19 H41 N2 O17	569.24	97.18		568.2314	568.2327	2.27	2.27	90.43	99.99	99.82	569.2387	1

m/z	Ion	Formula	Abundance
591.2204	(M+Na)+	C31 H36 Na O10	140670.1

Best	Formula (M)	Ion Formula	Calc m/z	Score	Cross S	Mass	Calc Mass	Diff (ppm)	Abs Diff (ppm)	Abund Match	Spacing Mat	Mass Match	m/z	DBE
✓	C31 H36 O10	C31 H36 Na O10	591.2201	99.96		568.2311	568.2308	-0.52	0.52	99.91	99.96	99.99	591.2204	14
	C32 H32 N4 O6	C32 H32 N4 Na O6	591.2214	99.9		568.2312	568.2322	1.82	1.82	99.85	99.98	99.89	591.2204	19
	C36 H32 N4 O S	C36 H32 N4 Na O S	591.2189	99.03		568.2312	568.2297	-2.59	2.59	97.11	99.85	99.78	591.2204	23
	C35 H36 O5 S	C35 H36 Na O5 S	591.2176	98.93		568.2311	568.2283	-4.93	4.93	97.7	99.85	99.22	591.2204	18
	C28 H40 O10 S	C28 H40 Na O10 S	591.2234	98.59		568.2311	568.2342	5.41	5.41	96.85	99.75	99.06	591.2204	9
	C33 H36 N4 O S2	C33 H36 N4 Na O S2	591.2223	97.91		568.2312	568.2331	3.33	3.33	93.72	99.49	99.64	591.2204	18
	C32 H40 O5 S2	C32 H40 Na O5 S2	591.2209	97.89		568.2312	568.2317	0.99	0.99	93.08	99.5	99.97	591.2204	13
	C23 H40 N2 O12 S	C23 H40 N2 Na O12 S	591.2194	97.87		568.2312	568.2302	-1.69	1.69	92.97	99.66	99.91	591.2204	5

Figure S34. The HR-Mass Spectrum of Compound 4 in MeOH

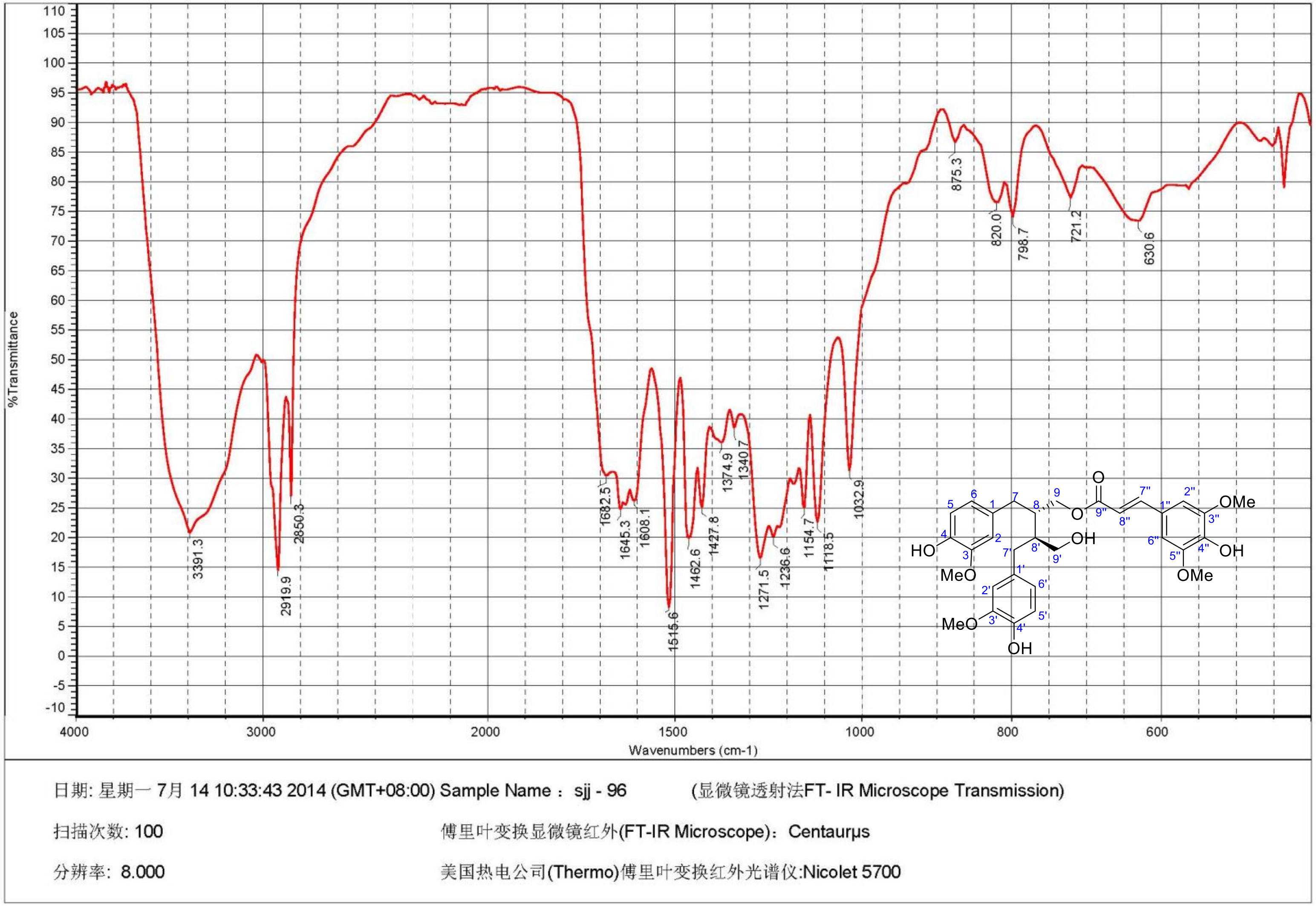

Figure S35. The IR Spectrum of Compound 4

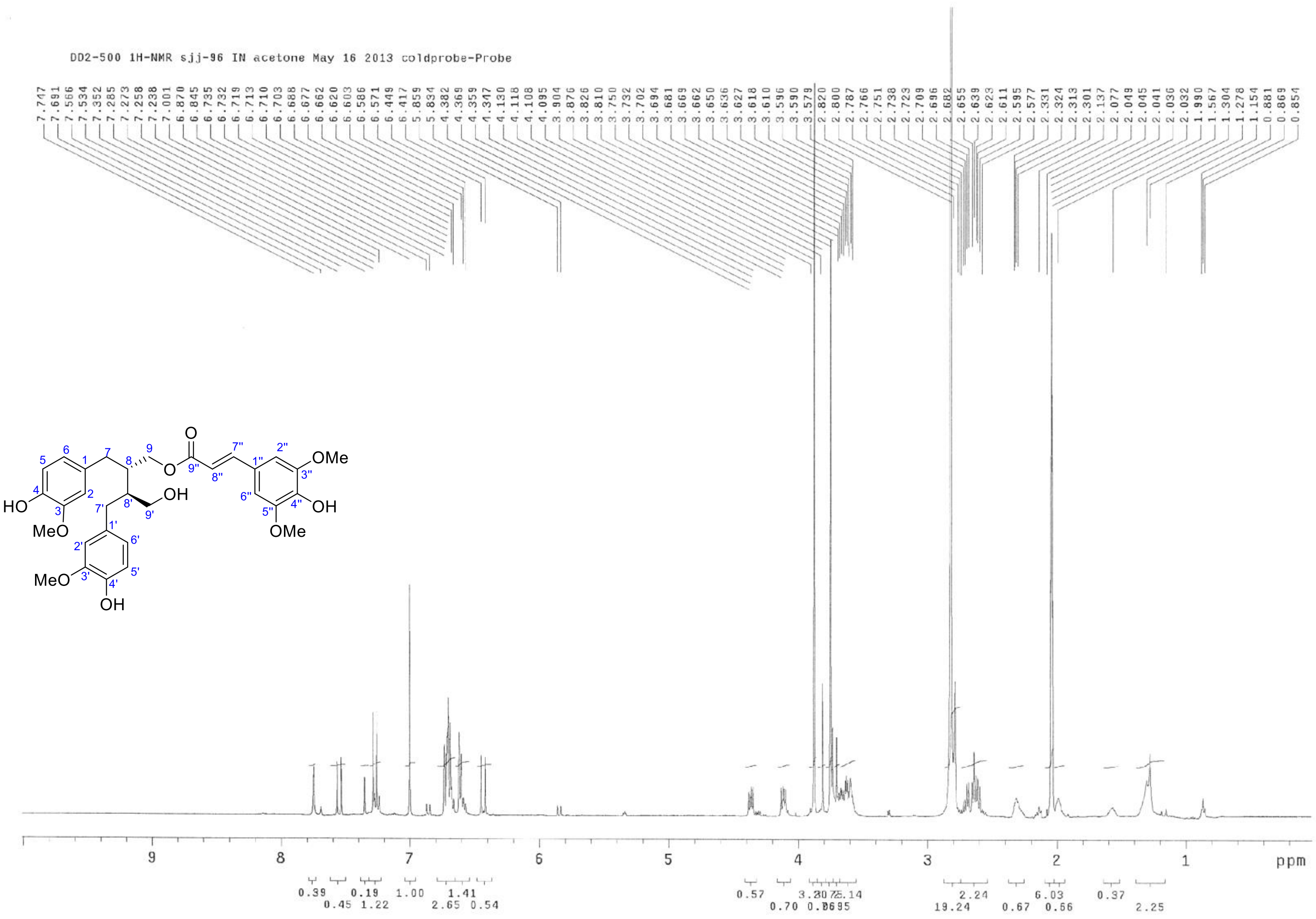

Figure S36.The ^{1}H NMR Spectrum of Compound 4 in Acetone-d_6 (500 MHz)

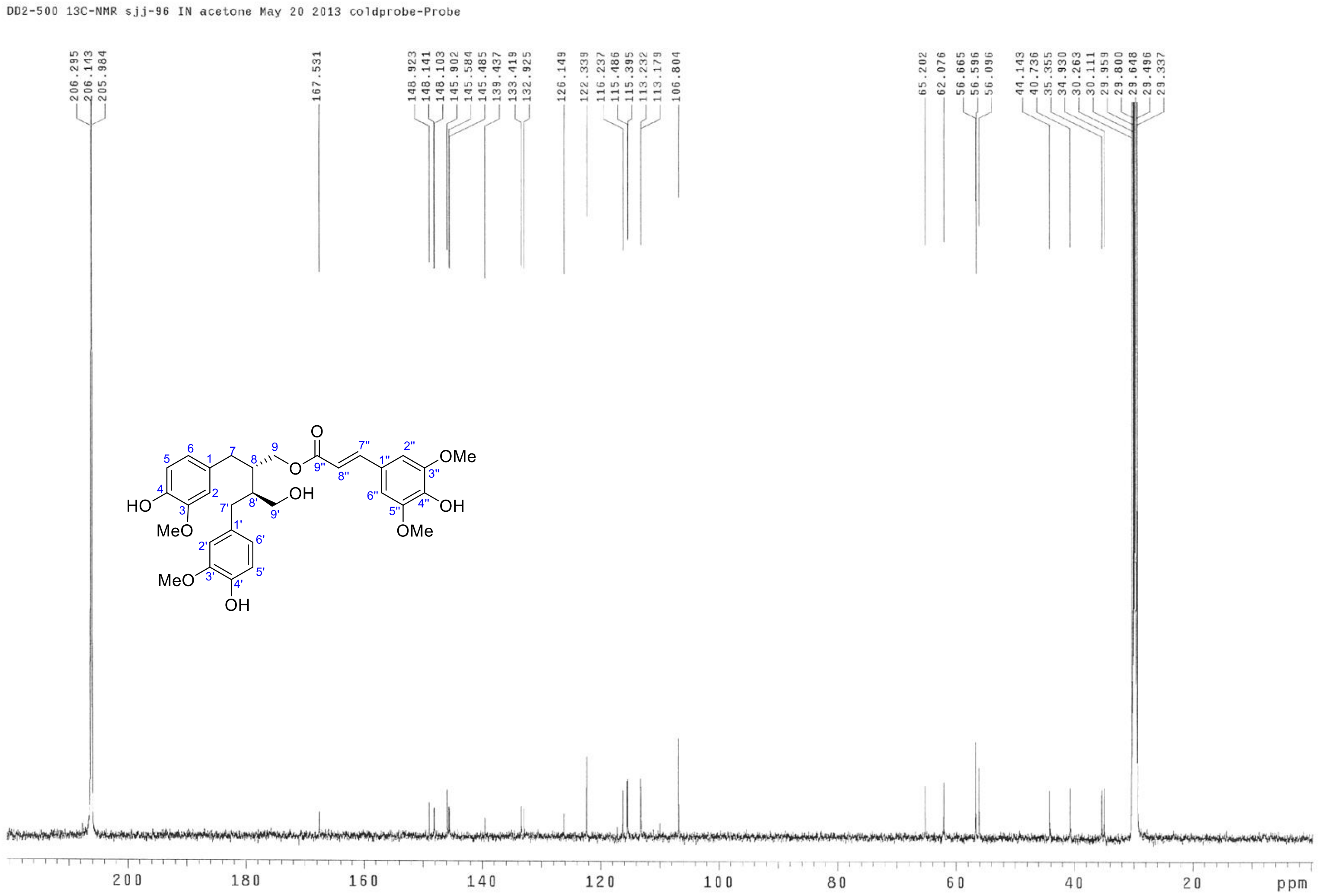

Figure S37. The 13 C NMR Spectrum of Compound 4 in Acetone-d_6 (125 MHz)

DD2-500 gCOSY sjj-96 IN acetone Jun 6 2013 coldprobe

Temp. 25.0 C / 298.1 K
Sample #12, Operator: vnmr1

Relax. delay 1.000 sec
Acq. time 0.150 sec
Width 5630.6 Hz
2D Width 5630.6 Hz
4 repetitions
128 increments
OBSERVE H1, 499.7700461 MHz
DATA PROCESSING
Sq. sine bell 0.075 sec
F1 DATA PROCESSING
Sq. sine bell 0.023 sec
FT size 2048 x 2048
Total time 10 min

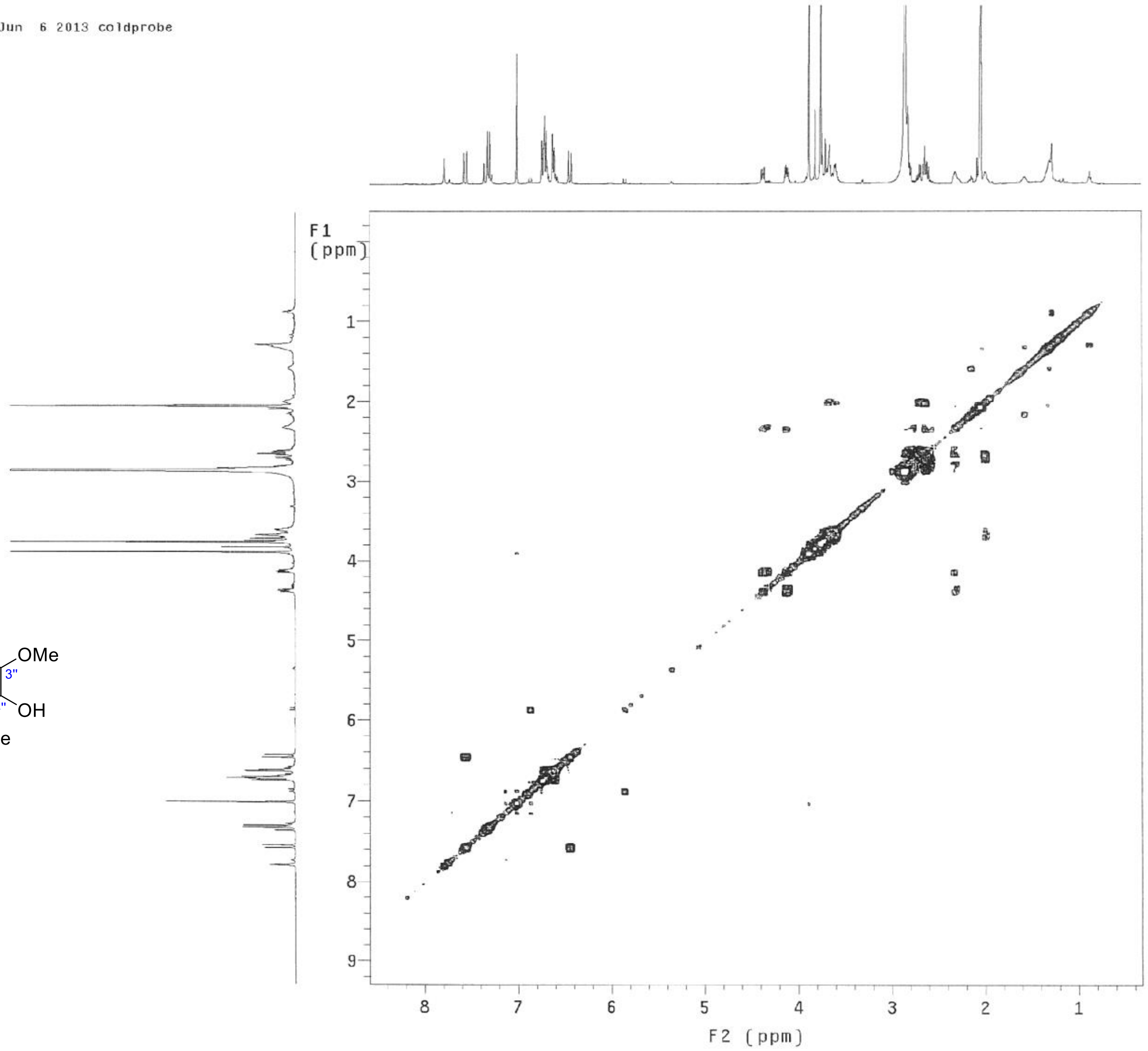

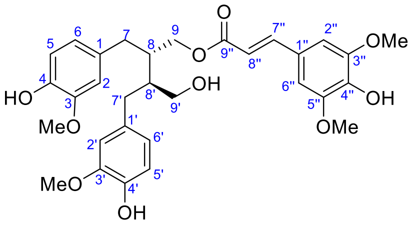

Figure S38. The ^{1}H-^{1}H COSY Spectrum of Compound 4 in Acetone-d_6 (500 MHz)

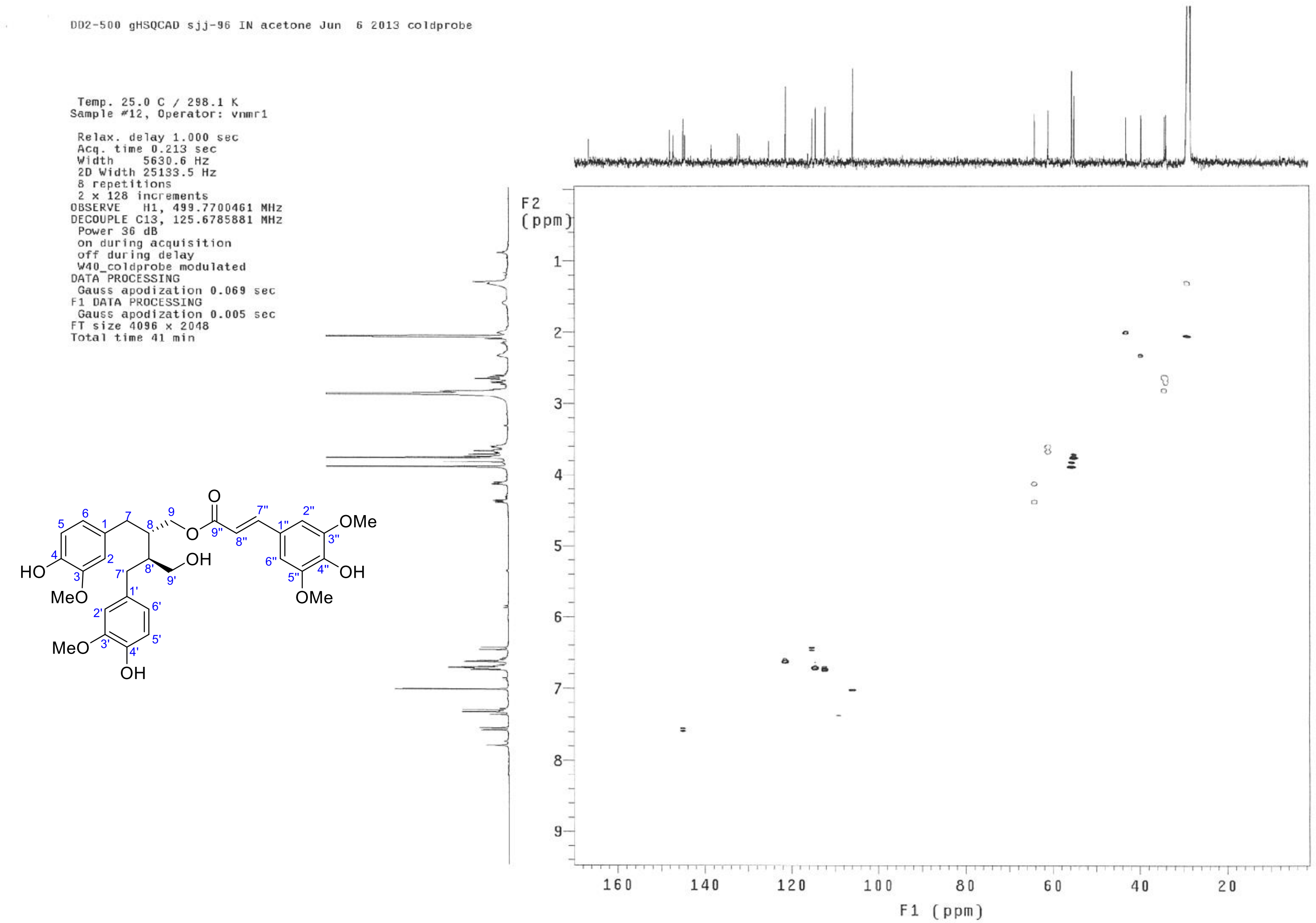

Figure S39. The HSQC Spectrum of Compound 4 in Acetone-d_6 (500 MHz)

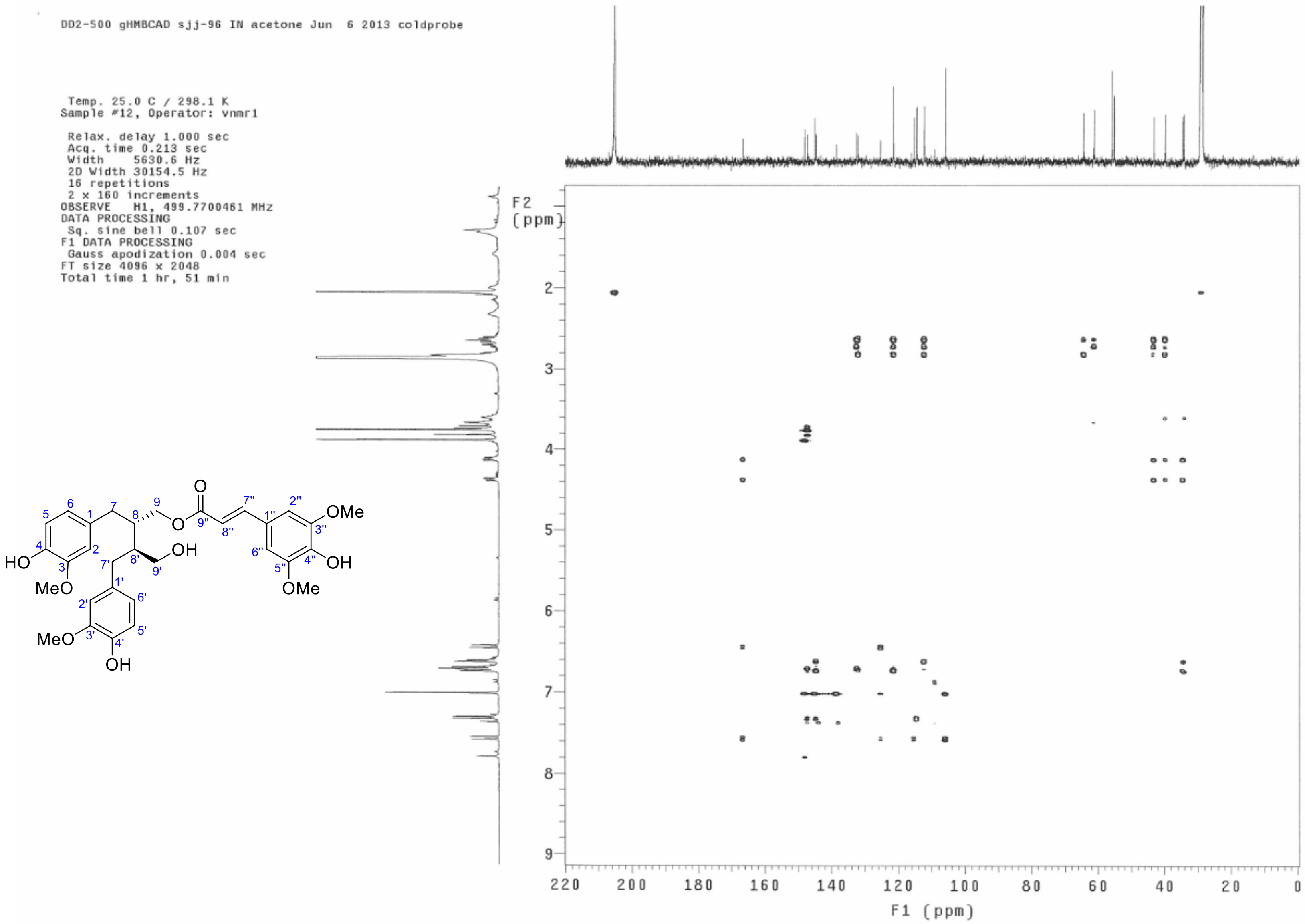

Figure 40. The HMBC Spectrum of Compound 4 in Acetone-d_6 (500 MHz)

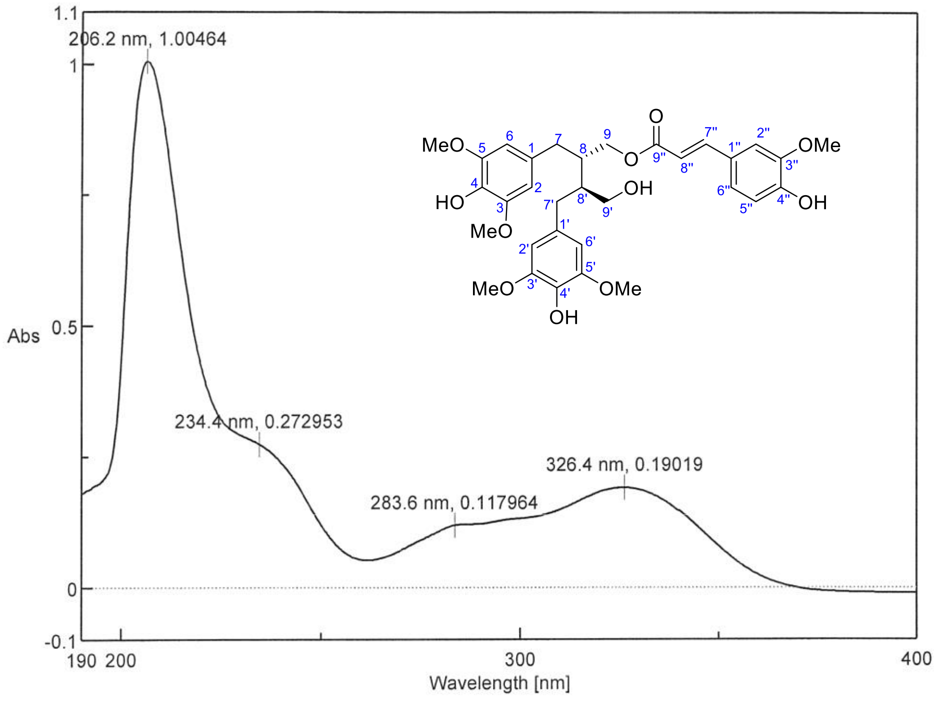

sjj-98a

[Comment]
Sample Name sjj-98a
Comment 0.02
User
Division UV
Company 324
[Measurement Information]
Instrument Name V-650
Model Name V-650
Serial No. A034461150

Accessory PSC-718
Accessory S/N A001761114
Position 1
Cell Length 10 mm
Temperature 19.94 C
Control Sonsor Holder
Monitor Sensor Holder
Start Mode Start immediately

Photometric Mode Abs
Measurement range 400 - 190 nm
Data pitch 0.2 nm
Band width(UV/Vis) 2.0 nm
Response Medium
Scanning speed 200 nm/min
Source Change 340 nm
Light Source D2/WI
Filter Exchange Step
Correction Baseline

[Data Information]
Creation Date 2014-11-18 20:26

Data array type Linear data array
Horizontal Wavelength [nm]
Vertical Abs
Start 400 nm
End 190 nm
Data pitch 0.2 nm
Data points 1051

Figure S41. The UV Spectra of Compound 5 in MeOH

Qualitative Analysis Report

Fragmentor Voltage	Collision Energy	Ionization Mode
135	0	Esi

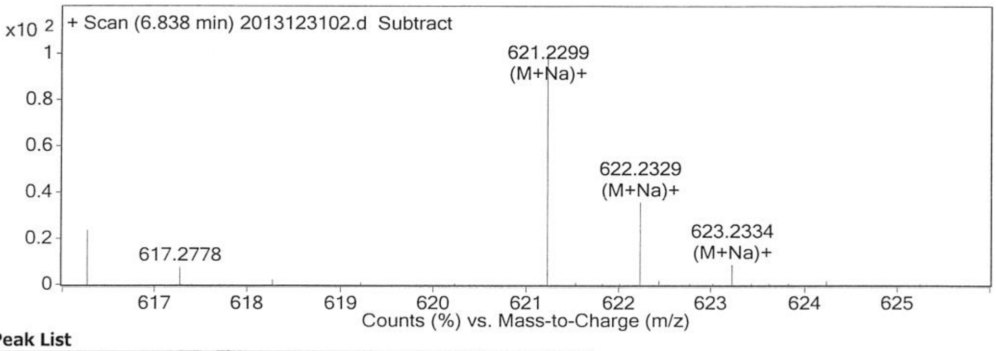

Peak List

m/z	z	Abund	Formula	Ion
158.1546		61508		
180.1368		21958		
405.1909	1	71364		
599.2465		19049		
616.2743	1	73966		
617.2778	1	24403		
621.2299	1	311553	C32 H38 Na O11	(M+Na)+
622.2329	1	110957	C32 H38 Na O11	(M+Na)+
623.2334	1	26969	C32 H38 Na O11	(M+Na)+
637.2031	1	44811		

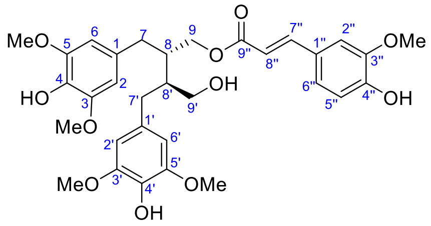

Formula Calculator Element Limits

Element	Min	Max
C	3	100
H	0	500
O	0	90
N	0	5
S	0	5
Cl	0	2
Br	0	0
Si	0	0
F	0	0
P	0	0

Formula Calculator Results

Formula	Best	Mass	Tgt Mass	Diff (ppm)	Ion Species	Score
C32 H38 O11	TRUE	598.2407	598.2414	1.24	C32 H38 Na O11	99.93
C33 H34 N4 O7		598.2407	598.2427	3.46	C33 H34 N4 Na O7	99.65
C36 H38 O6 S		598.2407	598.2389	-2.95	C36 H38 Na O6 S	99.18
C27 H38 N2 O13		598.2407	598.2374	-5.49	C27 H38 N2 Na O13	99.11
C37 H34 N4 O2 S		598.2407	598.2402	-0.73	C37 H34 N4 Na O2 S	99.06
C24 H42 N2 O13 S		598.2407	598.2408	0.13	C24 H42 N2 Na O13 S	98.42
C33 H42 O6 S2		598.2407	598.2423	2.68	C33 H42 Na O6 S2	98.04
C34 H38 N4 O2 S2		598.2407	598.2436	4.9	C34 H38 N4 Na O2 S2	97.88
C28 H42 N2 O8 S2		598.2407	598.2383	-4.06	C28 H42 N2 Na O8 S2	97.67
C42 H34 N2 S		598.2407	598.2443	6	C42 H34 N2 Na S	97.53

--- End Of Report ---

Figure S42. The HR-Mass Spectrum of Compound 5 in MeOH

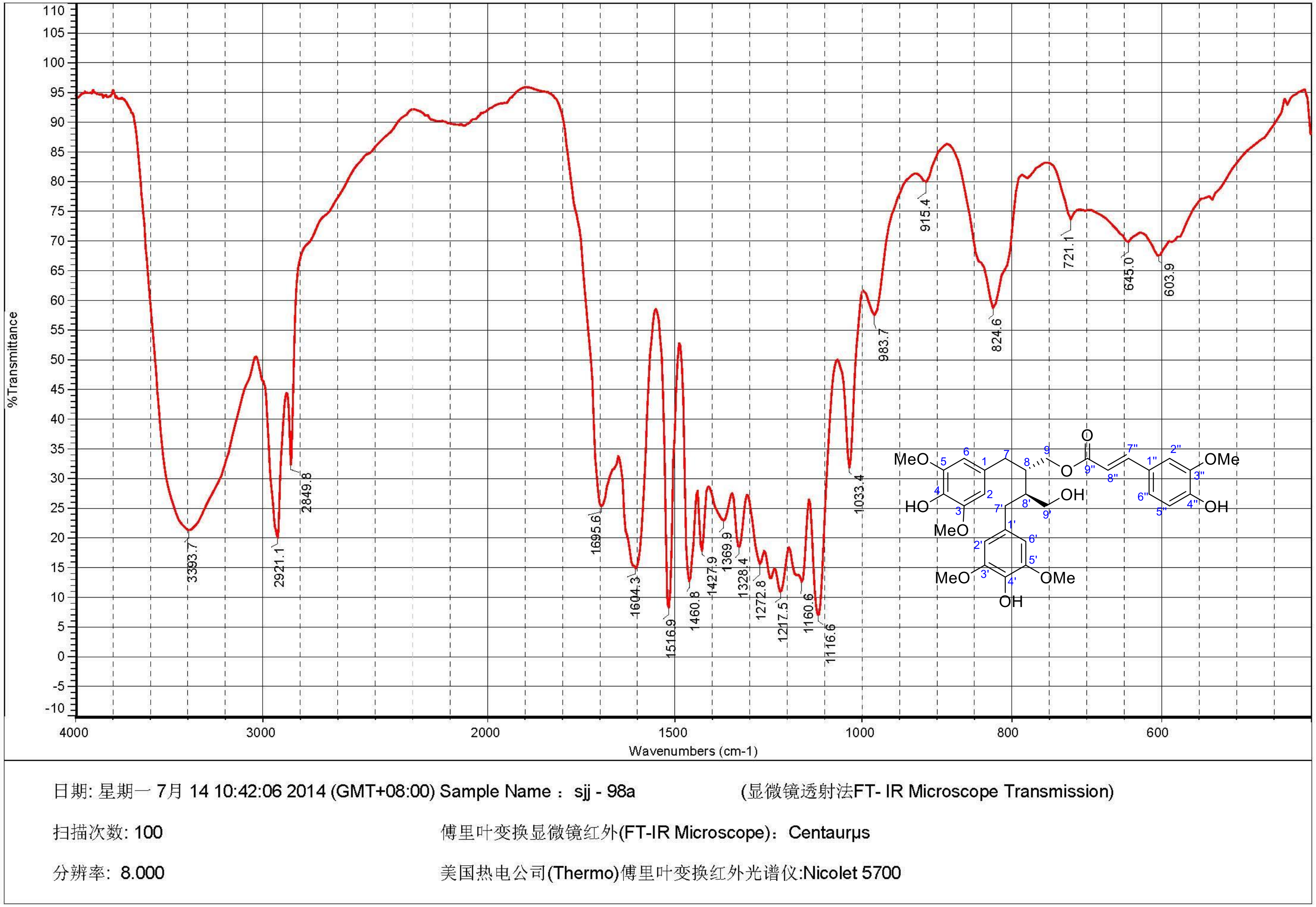

Figure S43. The IR Spectrum of Compound 5

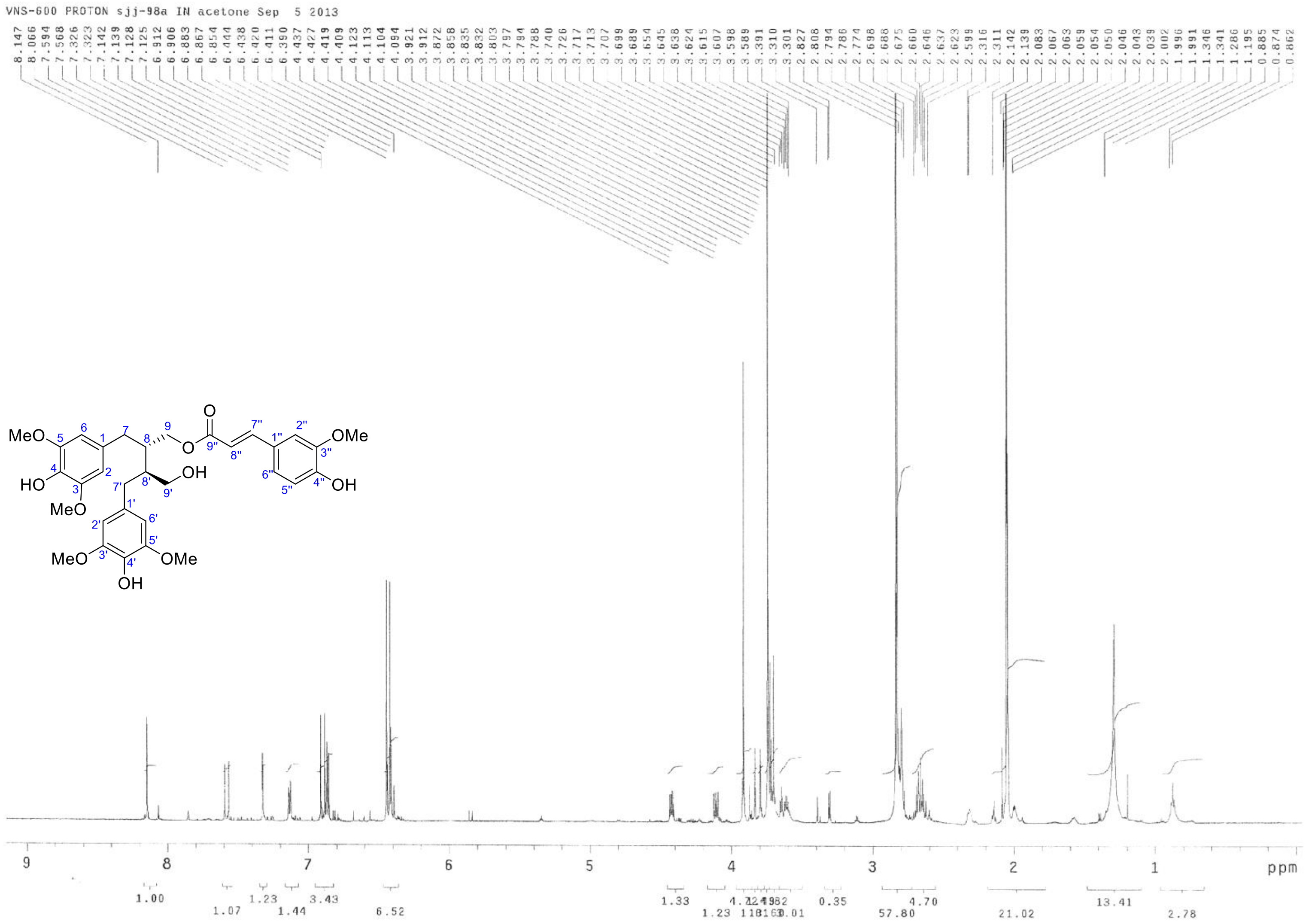

Figure S44.The ^{1}H NMR Spectrum of Compound 5 in Acetone-d_6 (600 MHz)

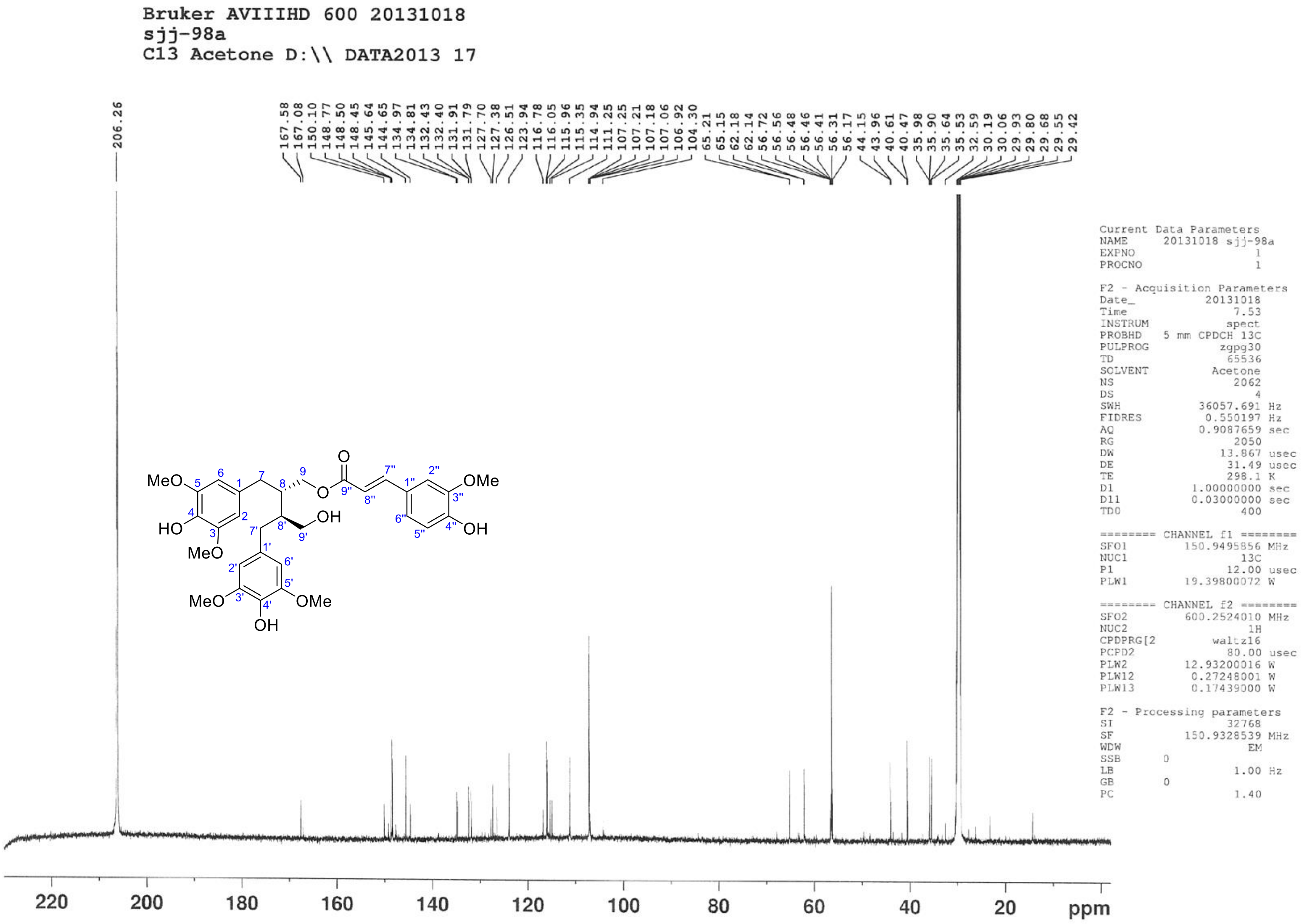

Figure S45.The [13] C NMR Spectrum of Compound 5 in Acetone-d_6 (150 MHz)

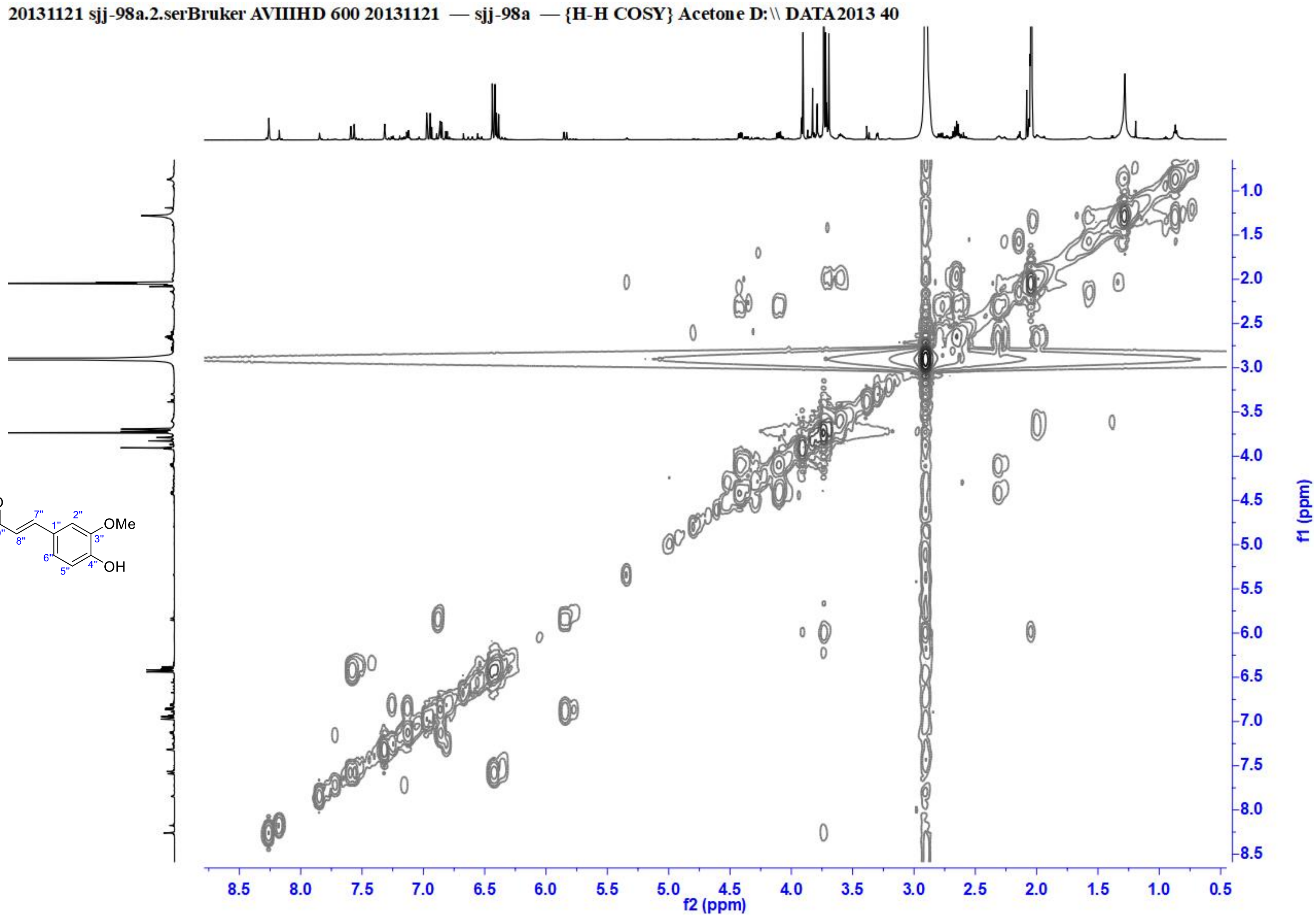

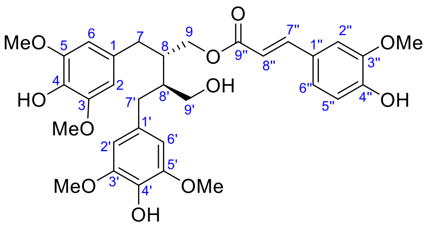

Figure S46. The ^{1}H-^{1}H COSY Spectrum of Compound 5 in Acetone-d_6 (600 MHz)

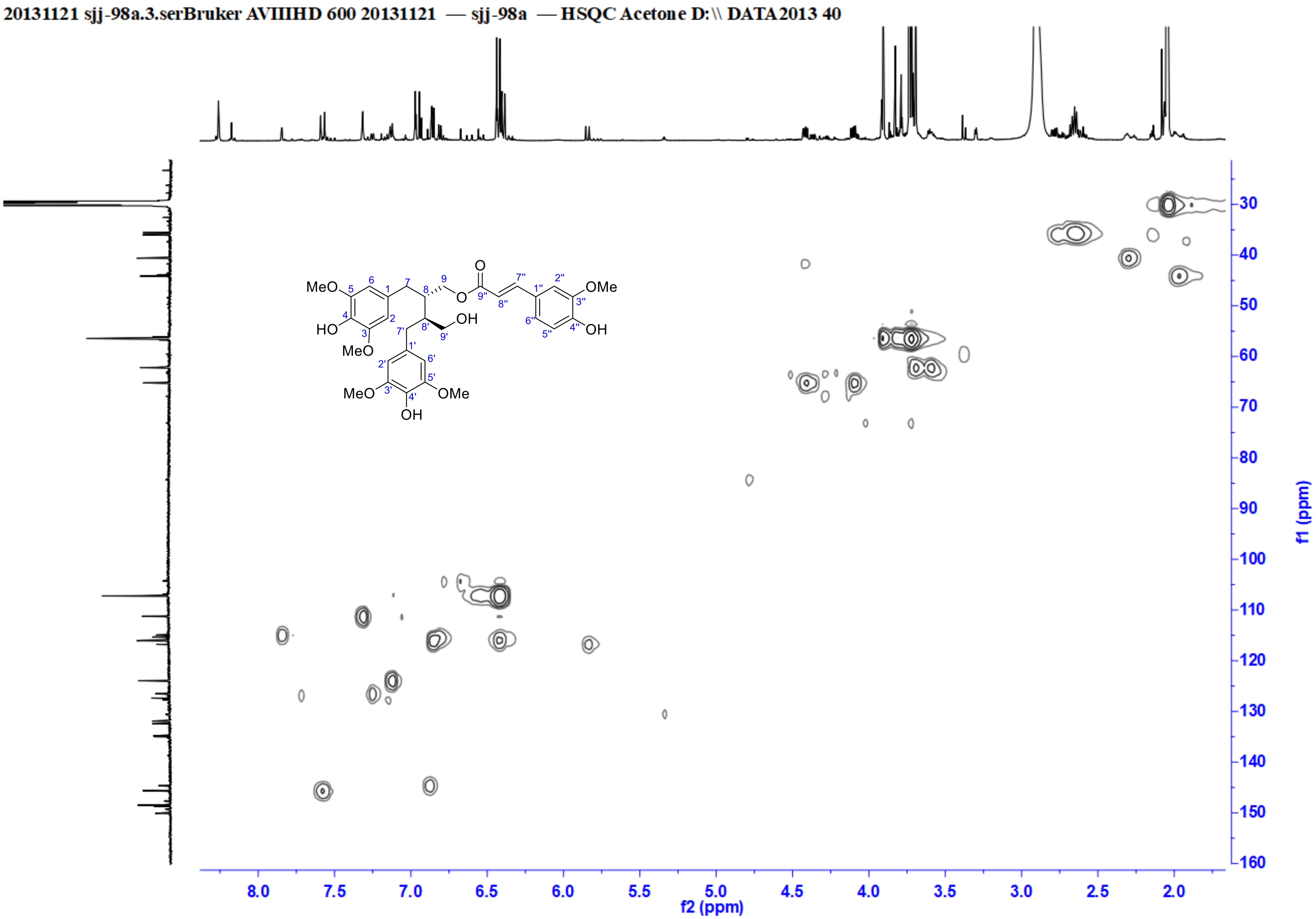

Figure S47. The HSQC Spectrum of Compound 5 in Acetone-d_6 (600 MHz)

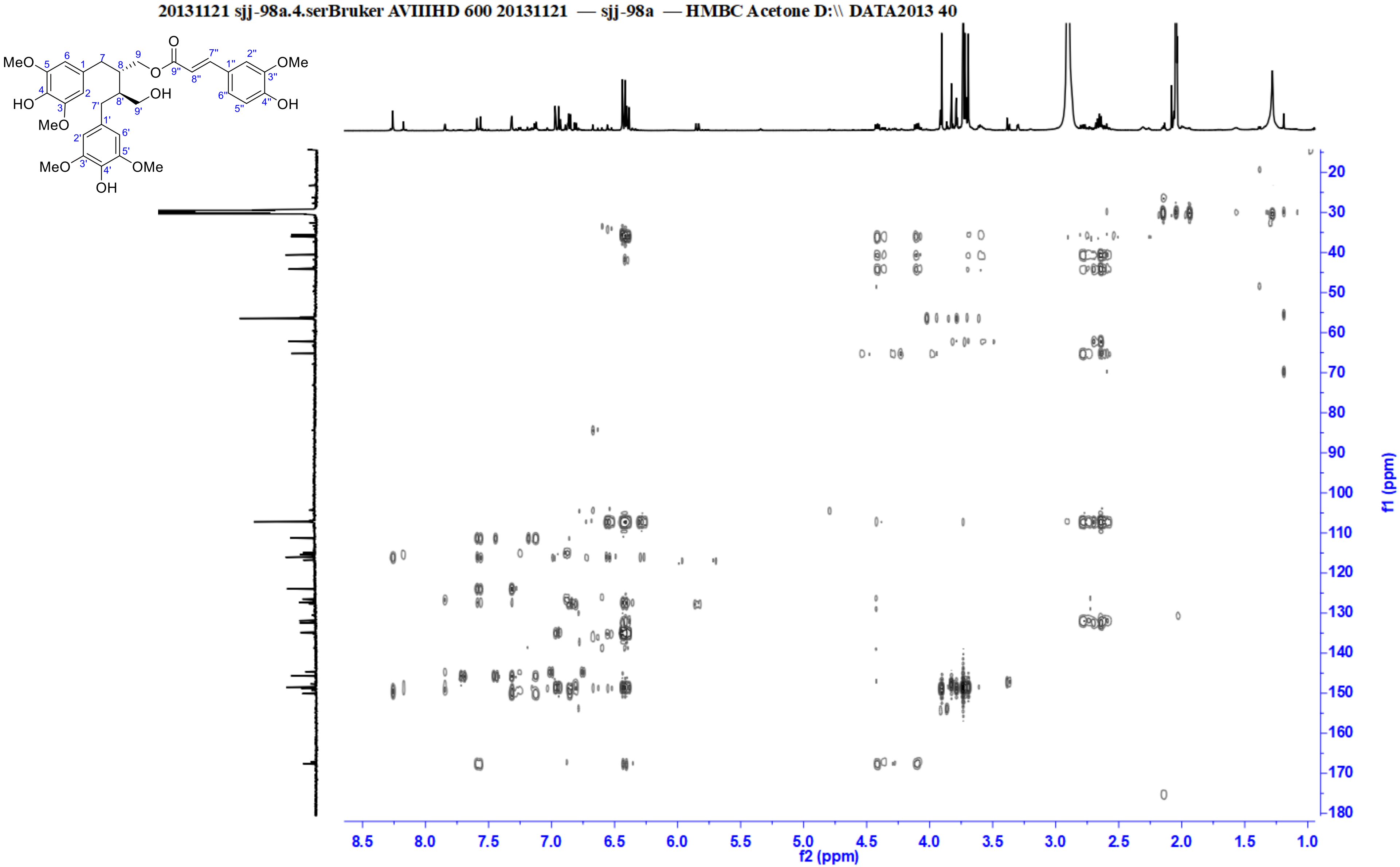

Figure S48. The HMBC Spectrum of Compound 5 in Acetone-d_6 (600 MHz)

Thermo Qexactive Focus Report

compound NO. : sjj-95a
Method : LCMS(compound)-low

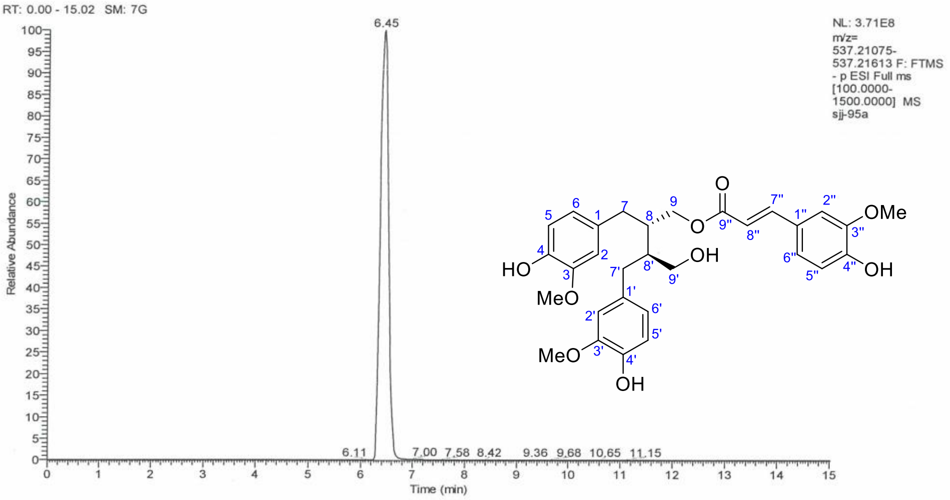

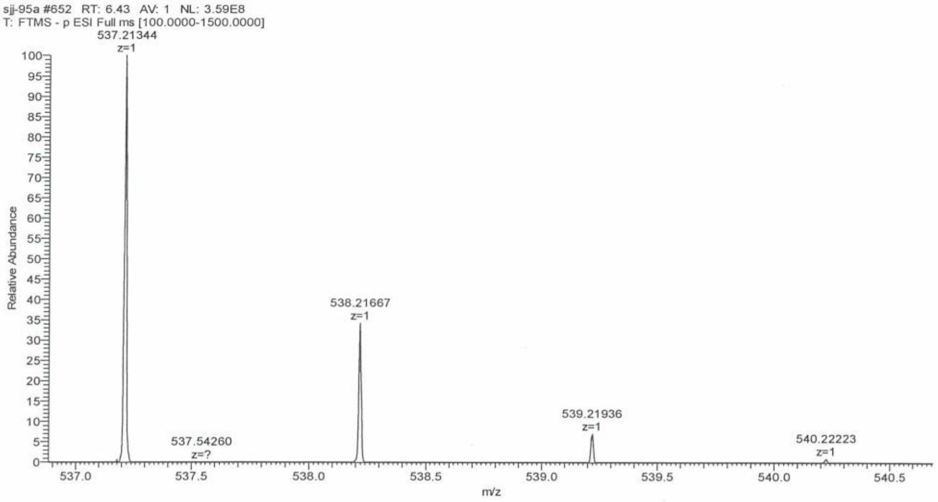

m/z	Theo. Mass	Delta (ppm)	RDB equiv.	Composition	
537.21344	537.21301	0.81	14.5	C30 H33 O9	M-H

Figure S49. The HR-Mass Spectrum of Compound 6 in MeOH

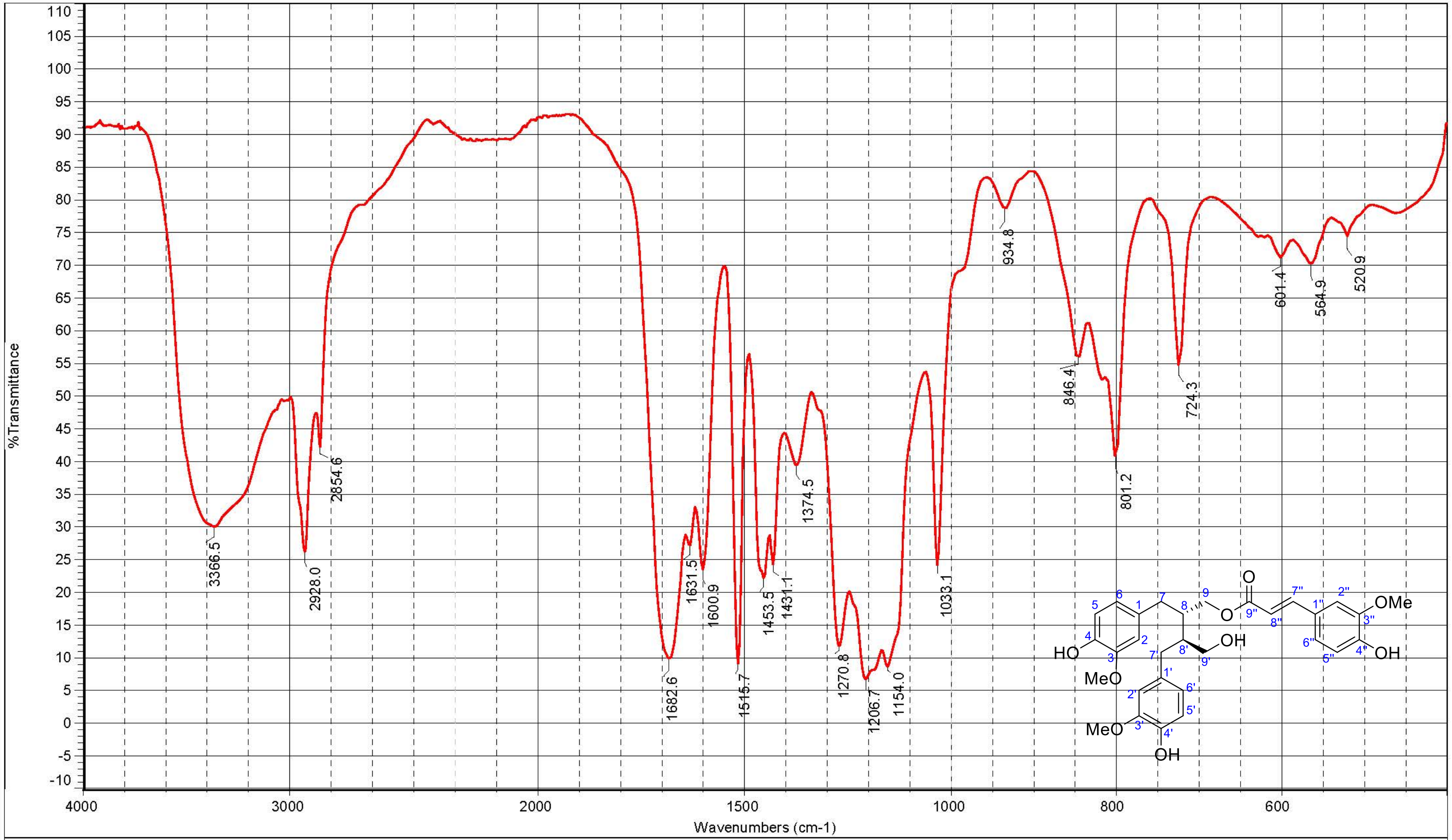

日期: 星期二 12月 11 12:10:49 2018 (GMT+08:00Sample Name： SJJ - 95a (显微镜透射法FT- IR Microscope Transmission)

扫描次数: 100 傅立叶变换显微镜红外(FT-IR Microscope)：Centaurμs

分辨率: 8.000 美国热电公司(Thermo)傅立叶变换红外光谱仪:Nicolet 5700

Figure S50.The IR Spectrum of 6

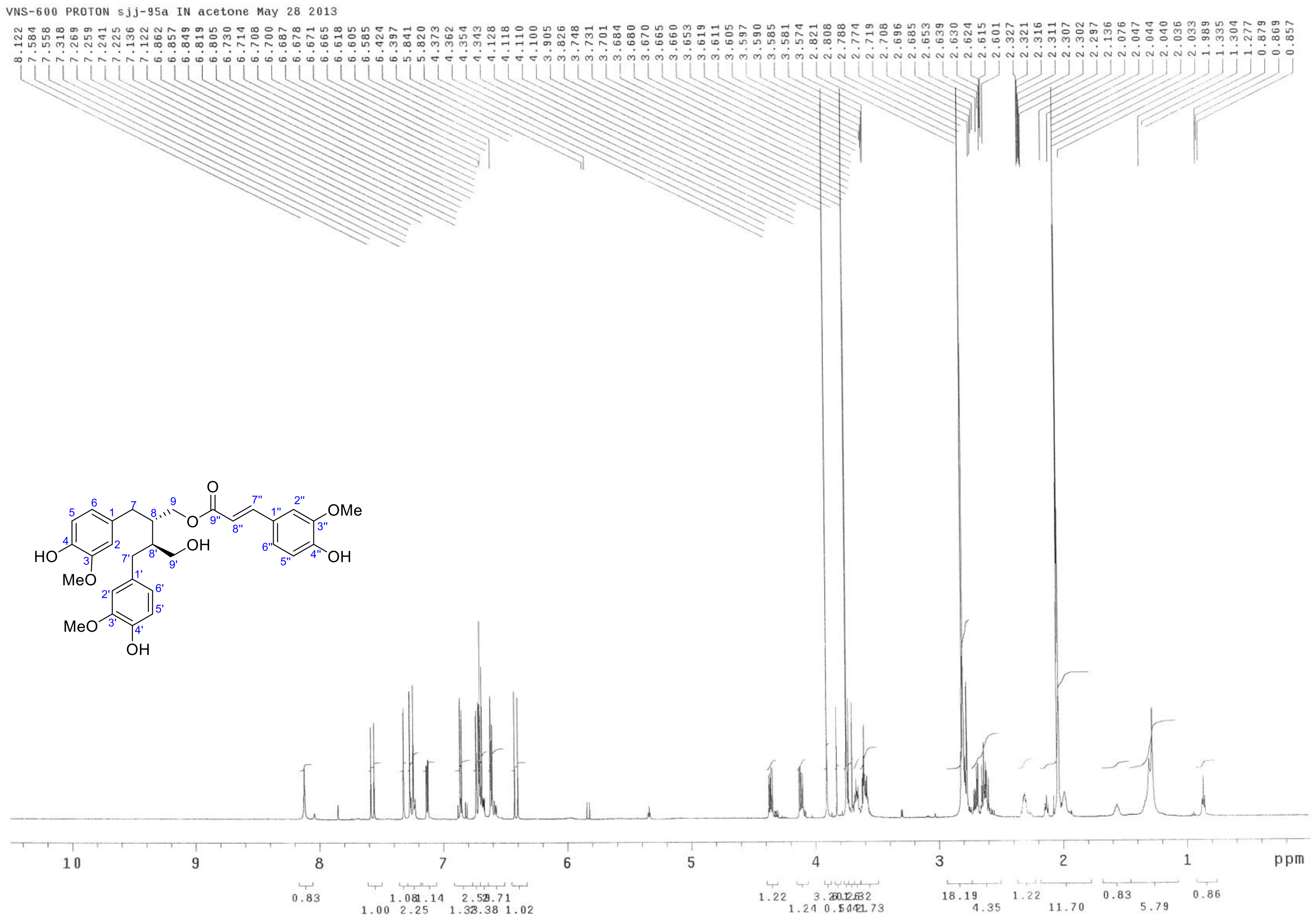

Figure S51. The ^{1}H NMR Spectrum of Compound 6 in Acetone-d_6 (600 MHz)

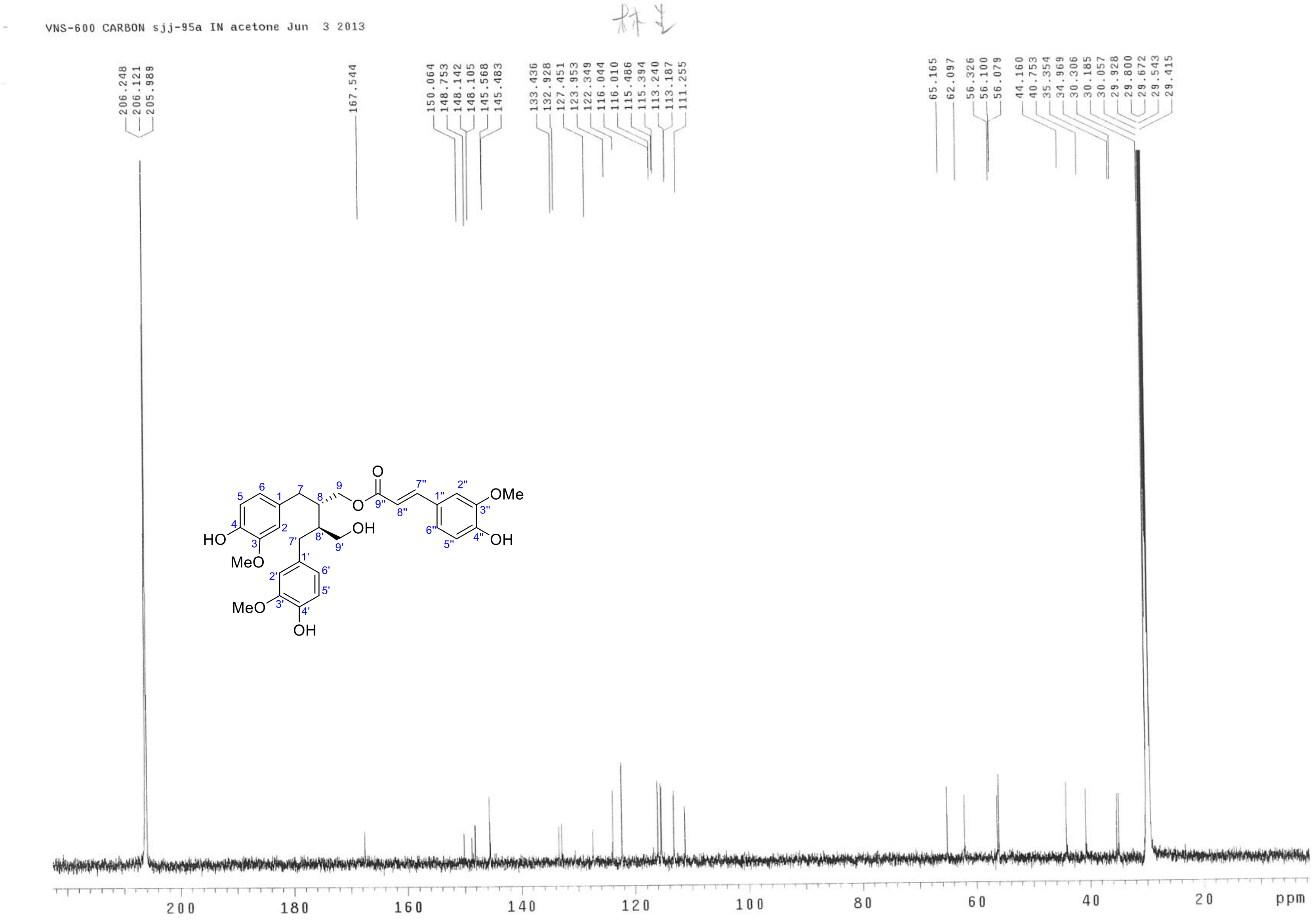

Figure S52.The [13] C NMR Spectrum of Compound 6 in Acetone-d_6 (150 MHz)

VNS-600 gCOSY sjj-95a IN acetone Jun 3 2013

Temp. 25.0 C / 298.1 K
Sample #7, Operator: vjwalk

Relax. delay 1.000 sec
Acq. time 0.150 sec
Width 5630.6 Hz
2D Width 5630.6 Hz
2 repetitions
256 increments
OBSERVE H1, 599.6908039 MHz
DATA PROCESSING
Sq. sine bell 0.075 sec
F1 DATA PROCESSING
Sq. sine bell 0.027 sec
FT size 2048 x 2048
Total time 10 min

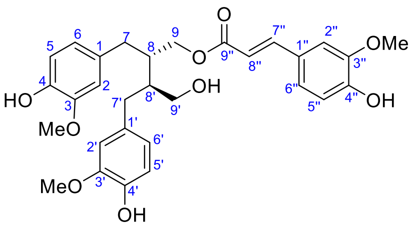

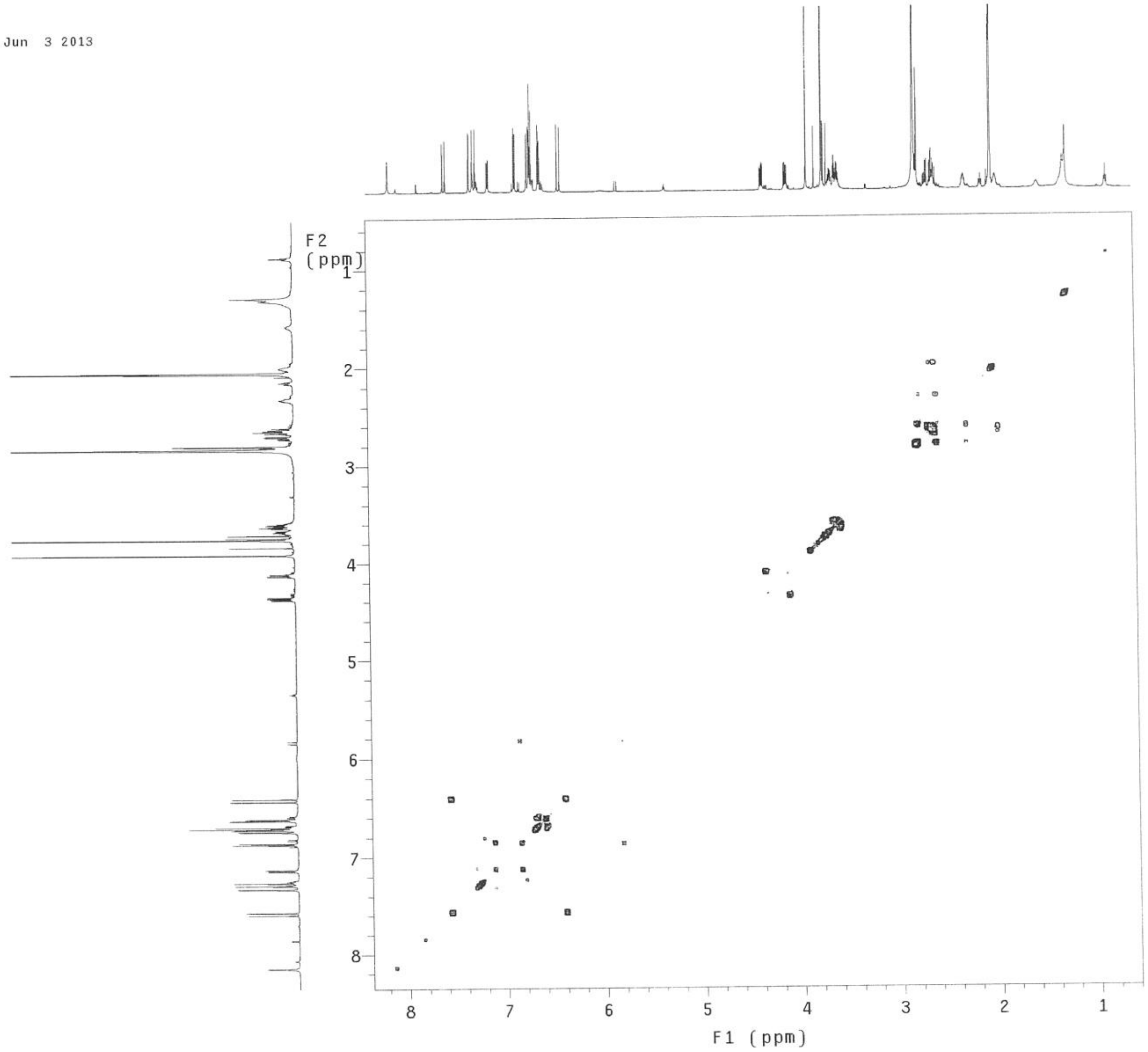

Figure S53. The ^{1}H-^{1}H COSY Spectrum of Compound 6 in Acetone-d_6 (600MHz)

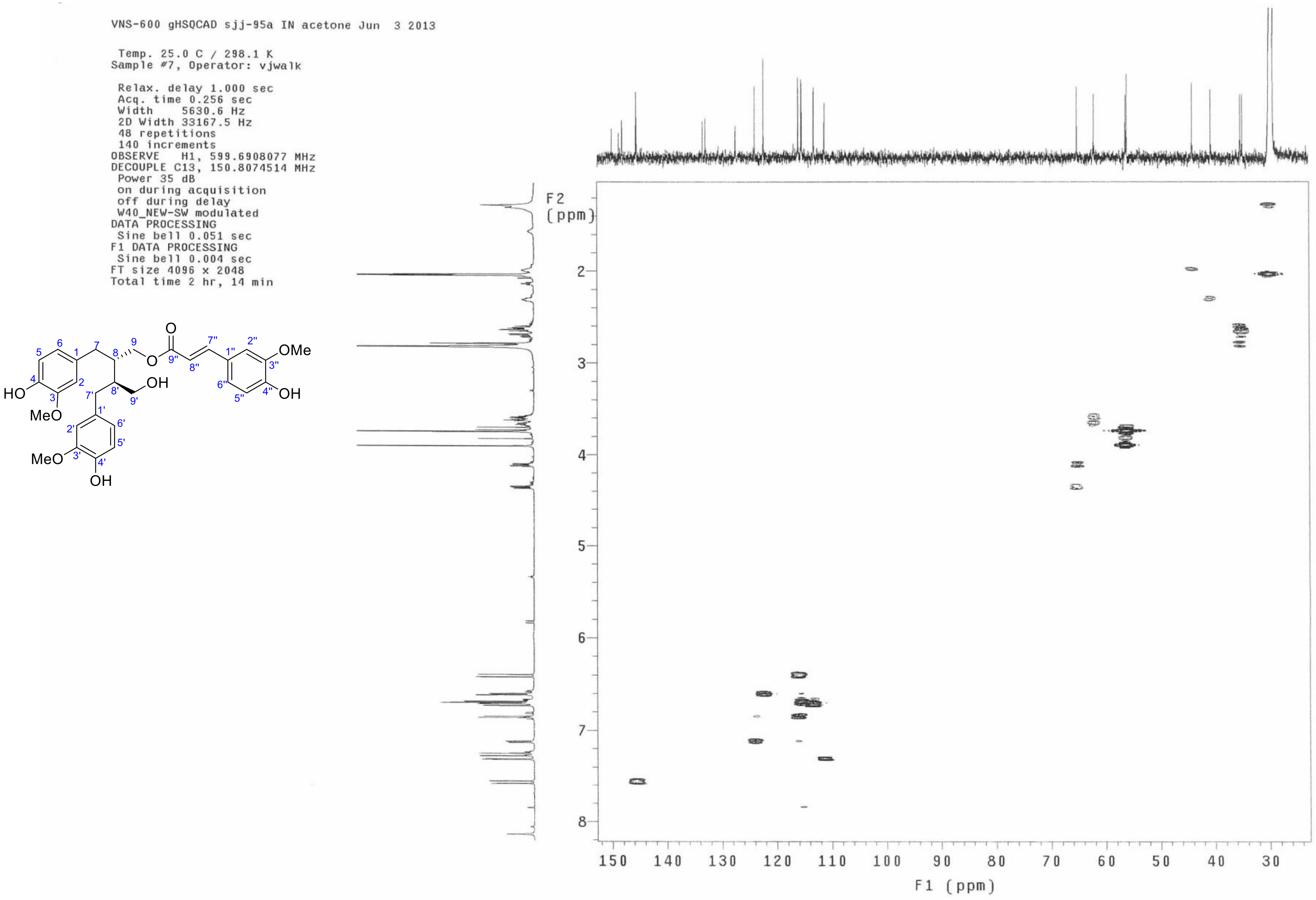

Figure S54. The HSQC Spectrum of Compound 6 in Acetone-d_6 (600MHz)

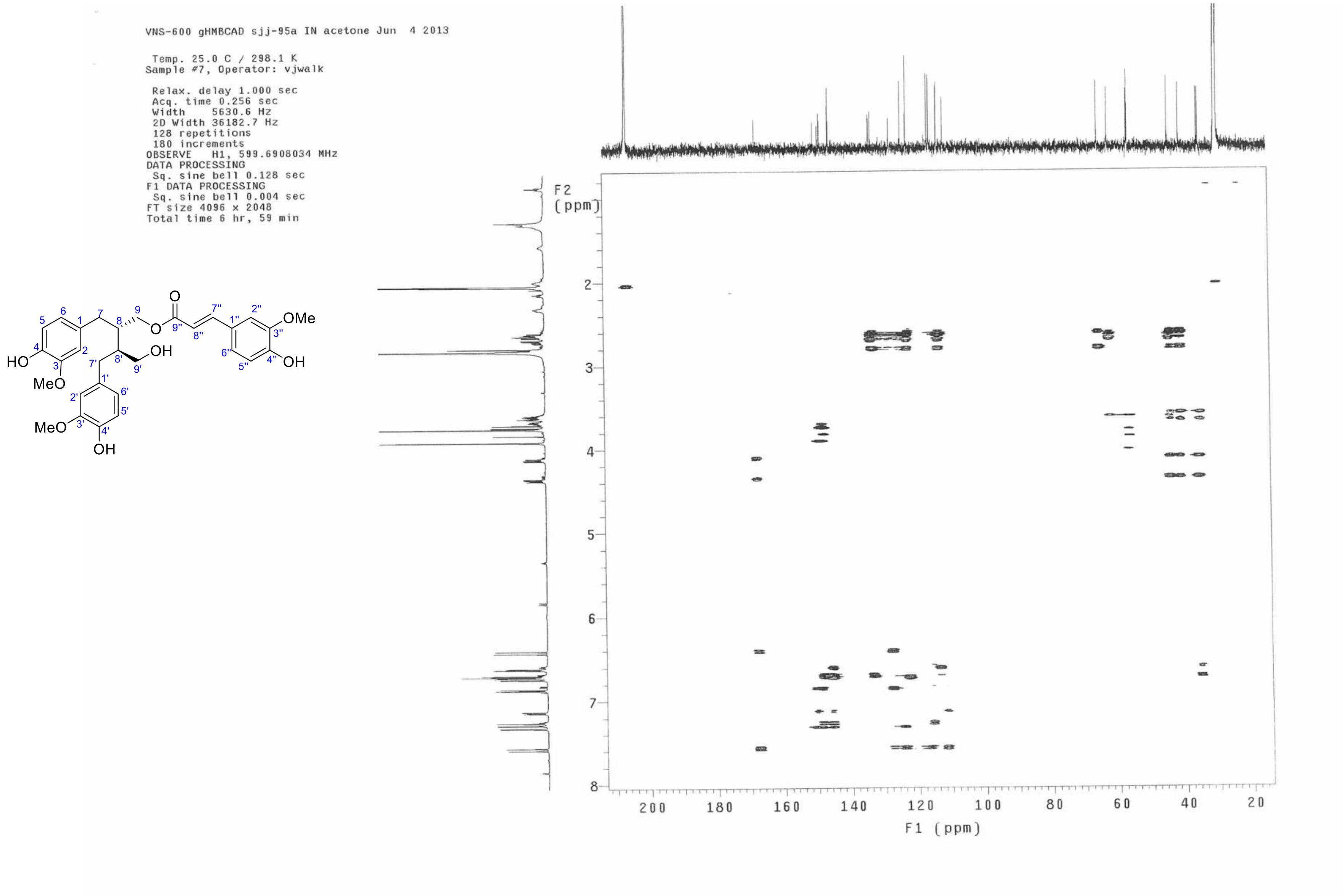

Figure S55. The HMBC Spectrum of Compound 6 in Acetone-d_6 (600MHz)

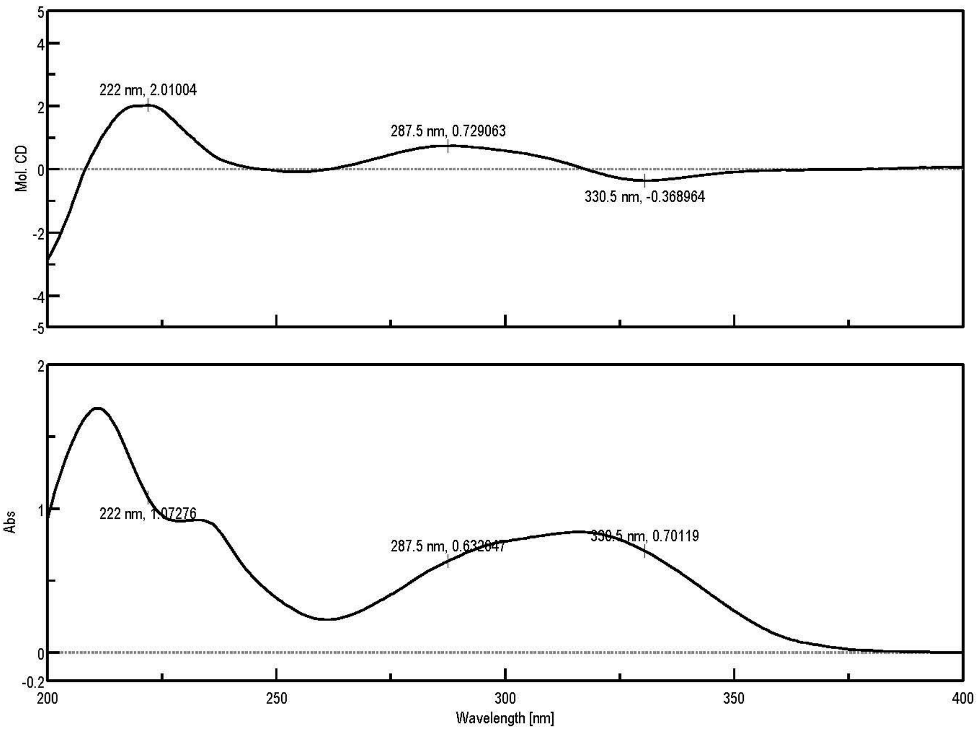

sjj-94-1-S-M.jws

[Comments]

Sample name	sjj-94
Comment	
User	
Division	
Company	dell

[Measurement Information]

Instrument Name	J-815
Model Name	J-815
Serial No.	A024461168
Accessory	Standard
Accessory S/N	A024461168
Cell Length	1 mm
Measurement date	2014/1/17 15:54
Photometric Mode	CD, HT, Abs
Measure Range	400 - 200 nm
Data pitch	0.5 nm
Sensitivity	Standard
D.I.T.	1 sec
Bandwidth	2.00 nm
Start Mode	Immediately
Scanning Speed	100 nm/min
Baseline Correction	Baseline
Shutter Control	Auto
PMT Voltage	Auto
Accumulations	3
Solvent	meoh
Concentration	0.406 (w/v)%

[Detailed Information]

Creation date	2014/1/20 9:58
Data array type	Linear data array * 3
Horizontal axis	Wavelength [nm]
Vertical axis(1)	Mol. CD
Vertical axis(2)	HT [V]
Vertical axis(3)	Abs
Start	400 nm
End	200 nm
Data interval	0.5 nm
Data points	401

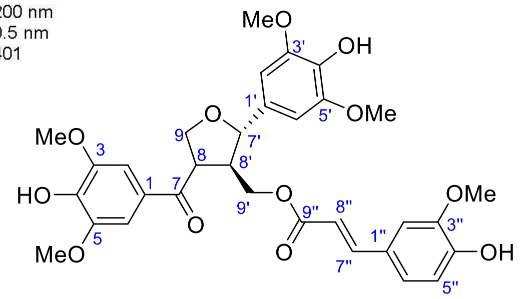

Figure S56. The UV and CD Spectra of Compound 7 in MeOH

Single Mass Spectrum Deconvolution Report

**Analysis Name:**	linsh142.d	**Instrument:**	LC-MSD-Trap-SL	**Print Date:**	10/27/2012 11:16:31 AM
**Method:**	def_lcms.m	**Operator:**	Operator	**Acq. Date:**	10/27/2012 11:04:46 AM
**Sample Name:**	sjj-94				
**Analysis Info:**					

Acquisition Parameter:

Mass Range Mode	Std/Normal	Trap Drive	29.0	Scan Begin	100 m/z
Ion Polarity	Positive	Octopole RF Amplitude	152.8 Vpp	Scan End	700 m/z
Ion Source Type	ESI	Capillary Exit	113.5 Volt	Averages	7 Spectra
Dry Temp (Set)	330 °C	Skimmer	40.0 Volt	Max. Accu Time	200000 µs
Nebulizer (Set)	15.00 psi	Oct 1 DC	12.00 Volt	ICC Target	10000
Dry Gas (Set)	5.00 l/min	Oct 2 DC	1.70 Volt	Charge Control	on

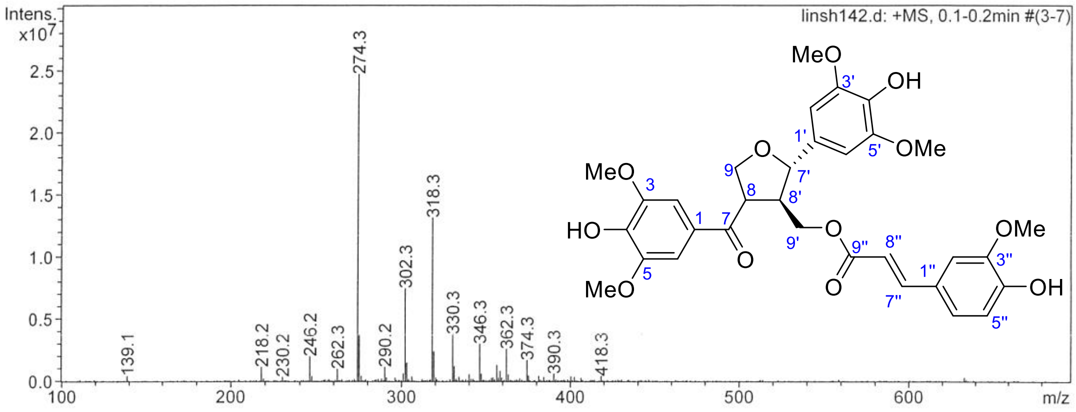

Component	Molecular Mass	Molecule	Absolute Abundance	Relative Abundance

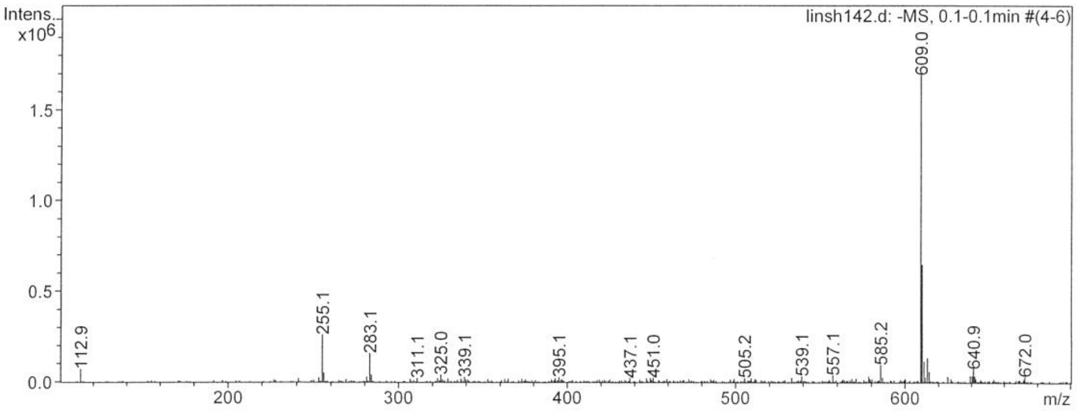

Component	Molecular Mass	Molecule	Absolute Abundance	Relative Abundance

Figure S57. The ESI-Mass Spectrum of Compound 7 in MeOH

Qualitative Analysis Report

Fragmentor Voltage	Collision Energy	Ionization Mode
135	0	Esi

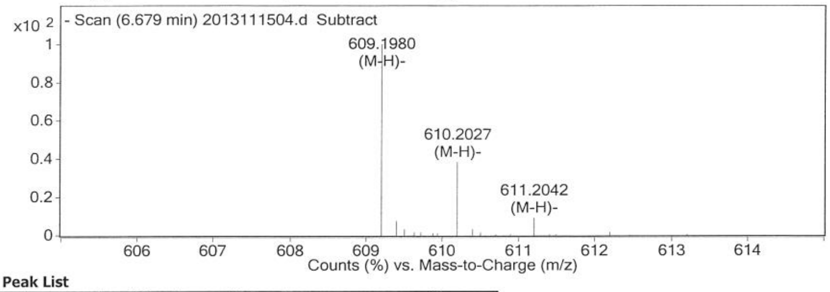

Peak List

m/z	z	Abund	Formula	Ion
609.198	1	2384458	C32 H33 O12	(M-H)-
609.401		180970		
610.2027	1	913744	C32 H33 O12	(M-H)-
611.2042	1	225099	C32 H33 O12	(M-H)-
631.1799		182535		
1241.384		153230		

Formula Calculator Element Limits

Element	Min	Max
C	3	100
H	0	500
O	0	90
N	0	5
S	0	5
Cl	0	2
Br	0	0
Si	0	0
F	0	0
P	0	0

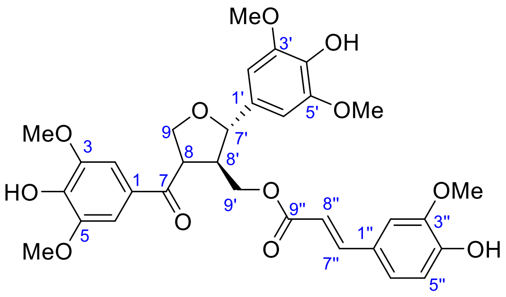

Formula Calculator Results

Formula	Best	Mass	Tgt Mass	Diff (ppm)	Ion Species	Score
C33 H30 N4 O8		610.2054	610.2064	1.64	C33 H29 N4 O8	99.89
C32 H34 O12	TRUE	610.2054	610.205	-0.53	C32 H33 O12	99.84
C37 H30 N4 O3 S		610.2054	610.2039	-2.47	C37 H29 N4 O3 S	99.23
C36 H34 O7 S		610.2054	610.2025	-4.64	C36 H33 O7 S	99.03
C42 H30 N2 O S		610.2054	610.2079	4.13	C42 H29 N2 O S	98.4
C29 H38 O12 S		610.2054	610.2084	4.98	C29 H37 O12 S	98.34
C45 H26 N2 O		610.2054	610.2045	-1.38	C45 H25 N2 O	98.32
C34 H34 N4 O3 S2		610.2054	610.2072	3.04	C34 H33 N4 O3 S2	97.96
C33 H38 O7 S2		610.2054	610.2059	0.87	C33 H37 O7 S2	97.83
C24 H38 N2 O14 S		610.2054	610.2044	-1.63	C24 H37 N2 O14 S	97.39

--- End Of Report ---

Figure S58. The HR-Mass Spectrum of Compound 7 in MeOH

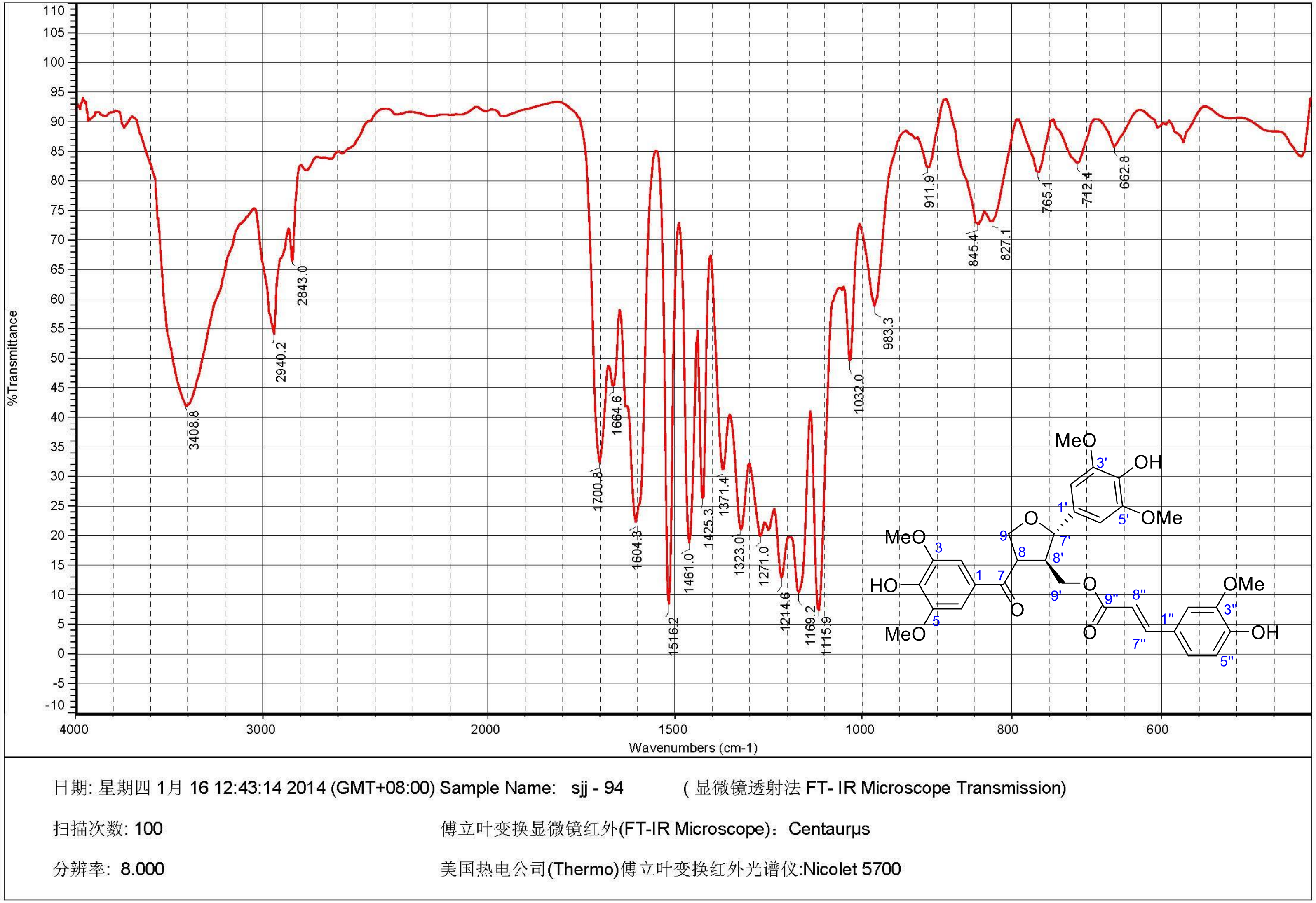

Figure S59. The IR Spectrum of Compound 7

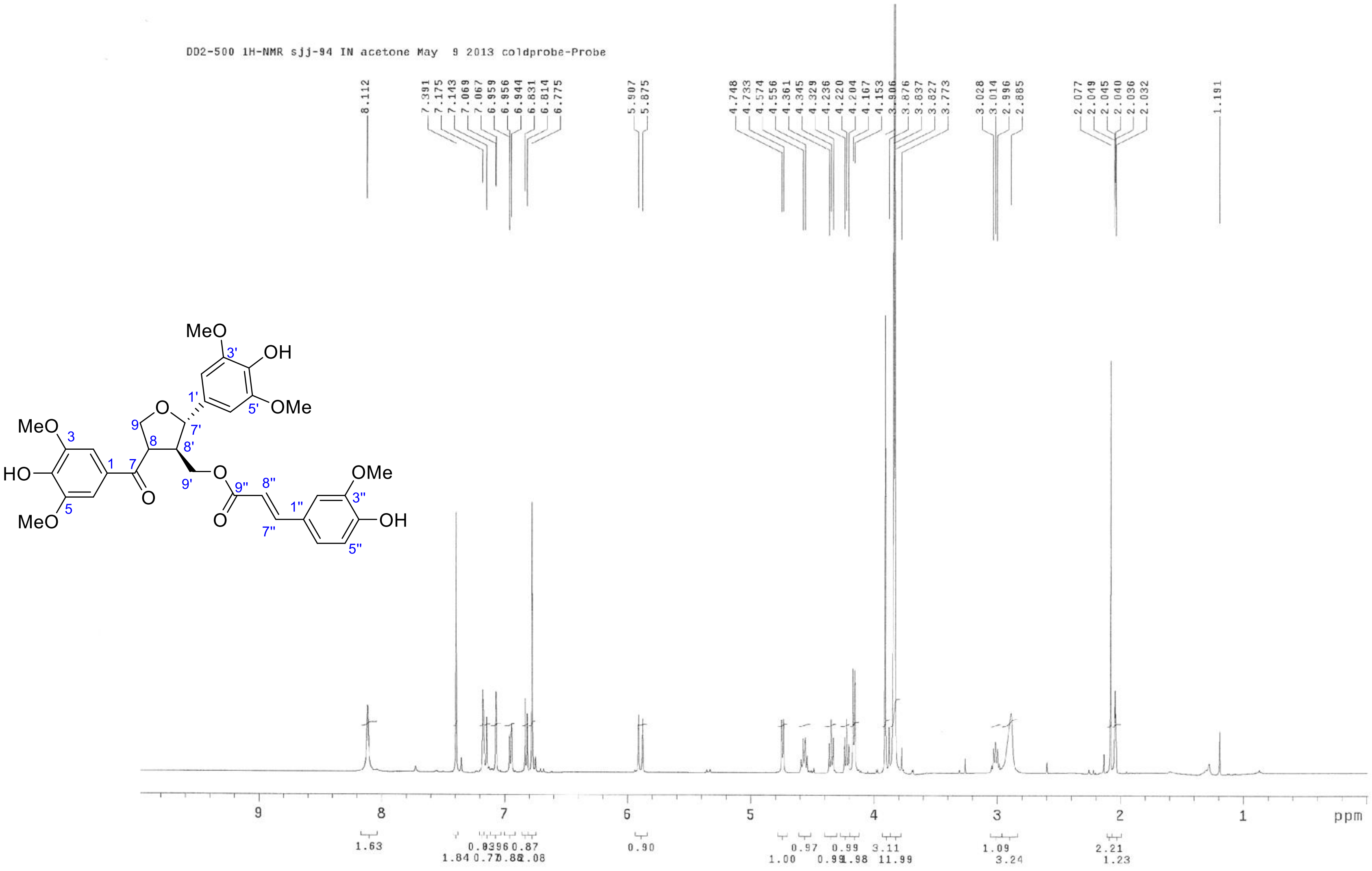

Figure S60.The ^{1}H NMR Spectrum of Compound 7 in Acetone-d_6 (500 MHz)

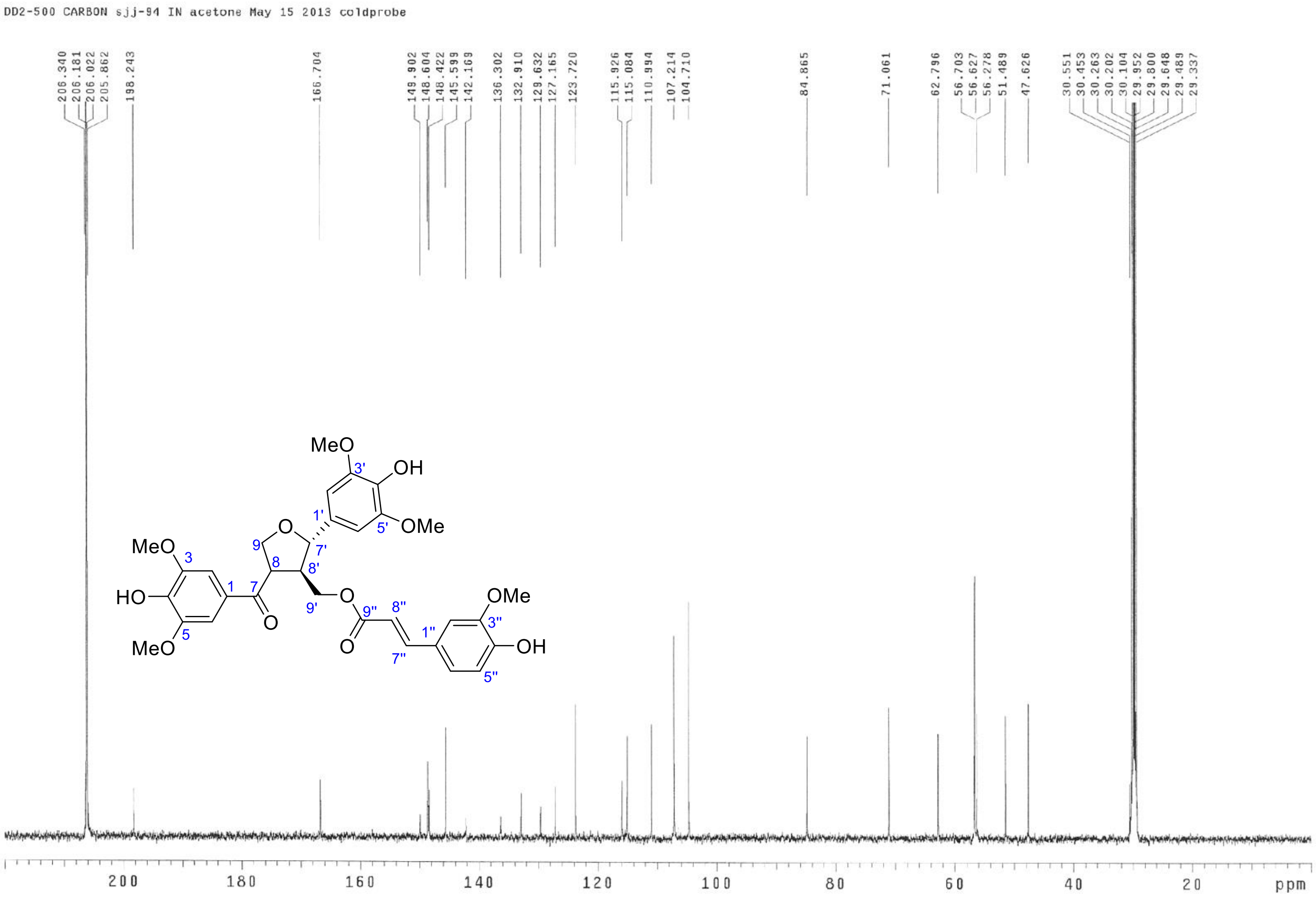

Figure S61. The 13 C NMR Spectrum of Compound 7 in Acetone-d_6 (125 MHz)

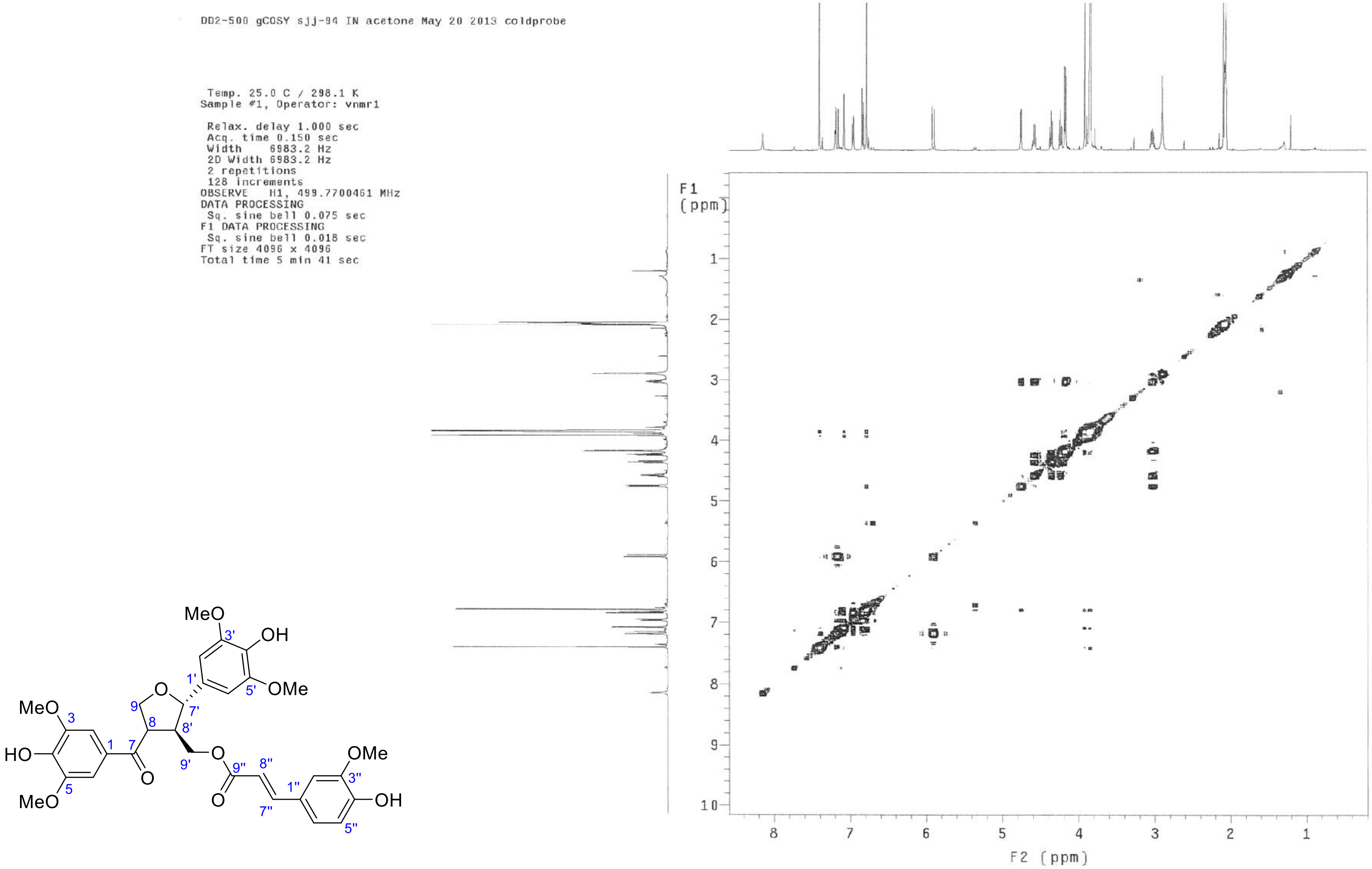

Figure S62. The ^{1}H-^{1}H COSY Spectrum of Compound 7 in Acetone-d_6 (500 MHz)

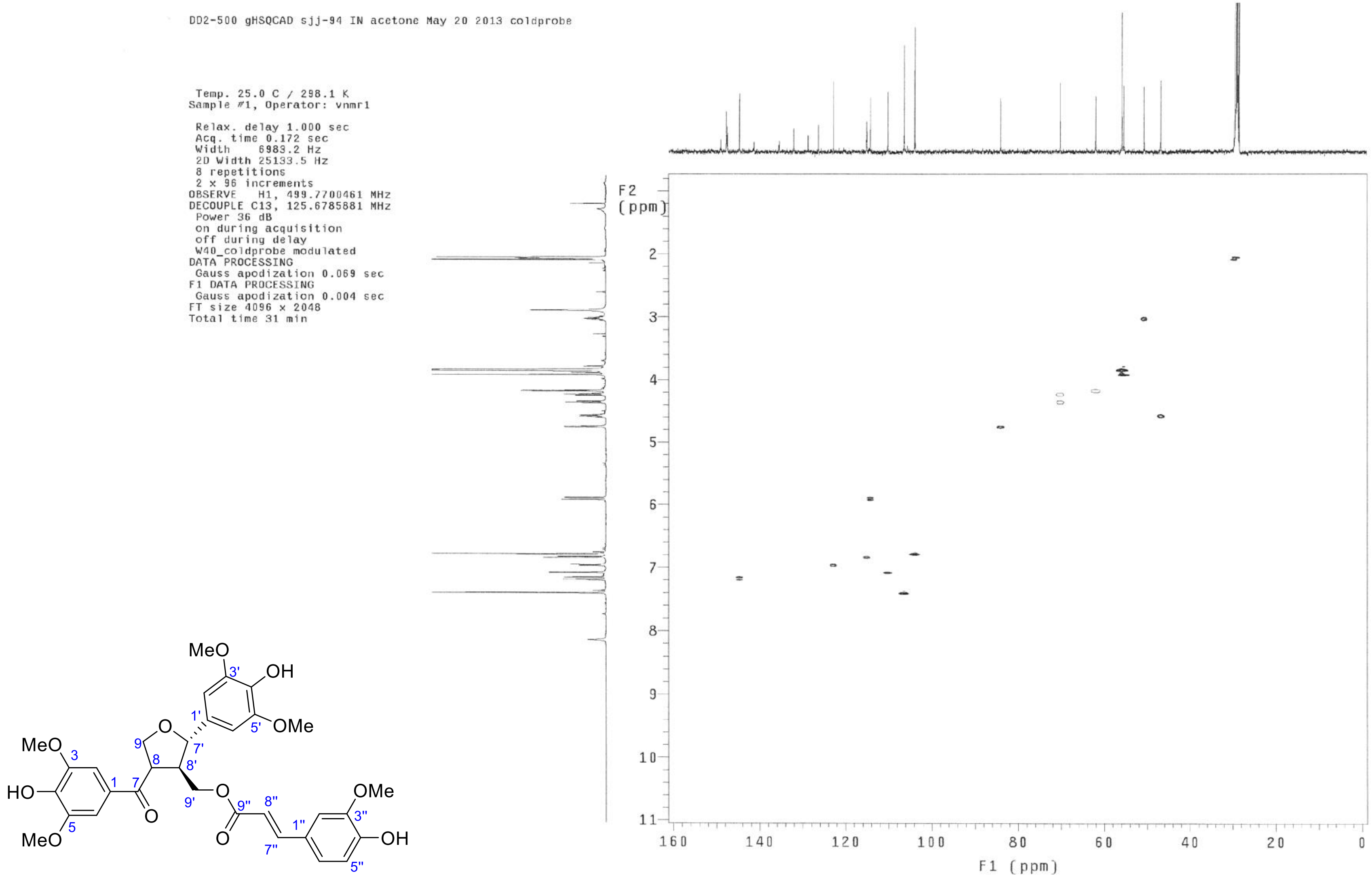

Figure S63. The HSQC Spectrum of Compound 7 in Acetone-d_6 (500 MHz)

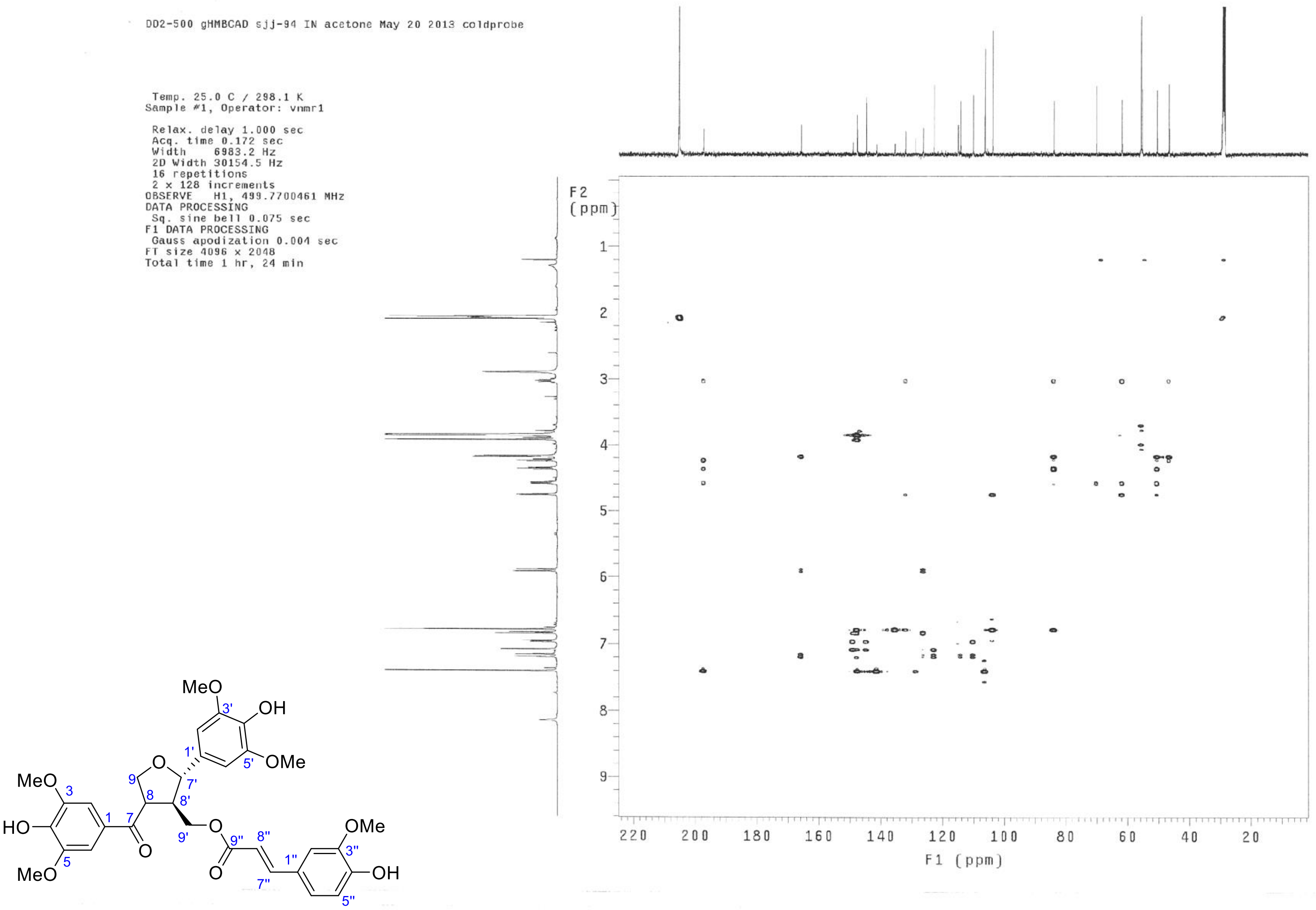

Figure S64. The HMBC Spectrum of Compound 7 in Acetone-d_6 (500 MHz)

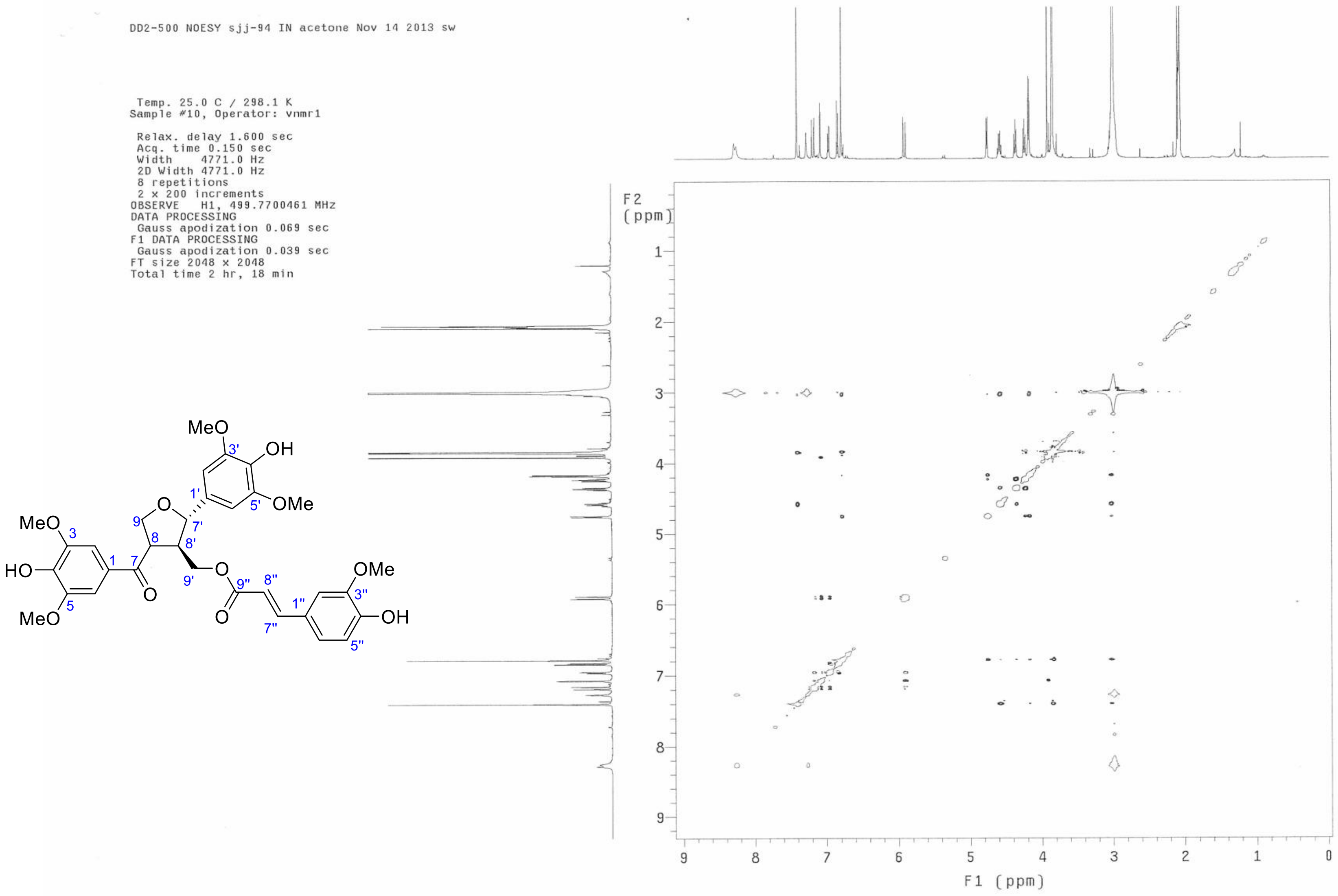

Figure S65. The NOESY Spectrum of Compound 7 in Acetone-d_6 (500 MHz)

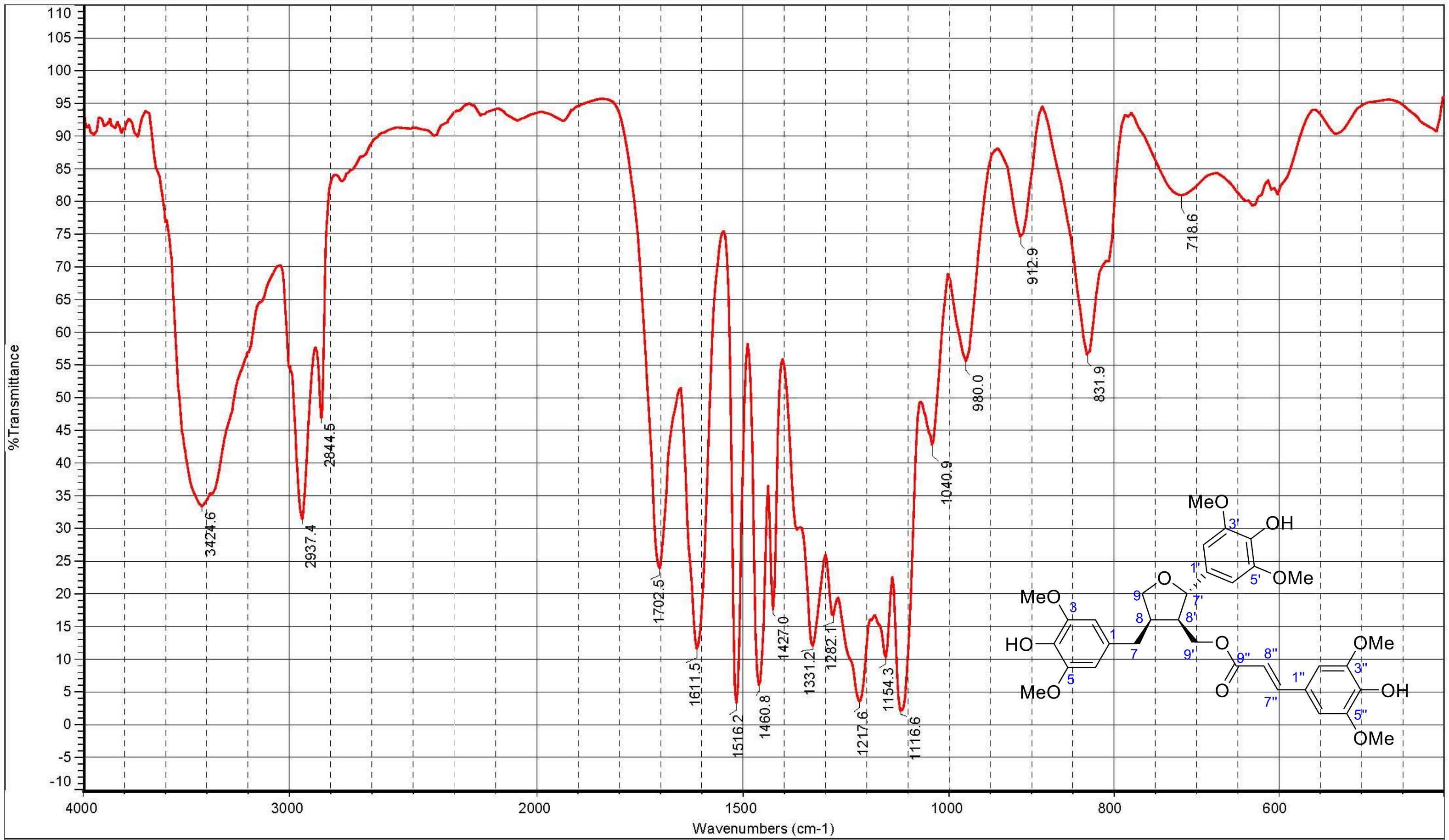

日期: 星期四 1月 16 12:35:04 2014 (GMT+08:00) Sample Name: sjj - 131 (显微镜透射法 FT- IR Microscope Transmission)

扫描次数: 100 傅立叶变换显微镜红外(FT-IR Microscope)：Centaurμs

分辨率: 8.000 美国热电公司(Thermo)傅立叶变换红外光谱仪:Nicolet 5700

Figure S66. The IR Spectrum of Compound 8

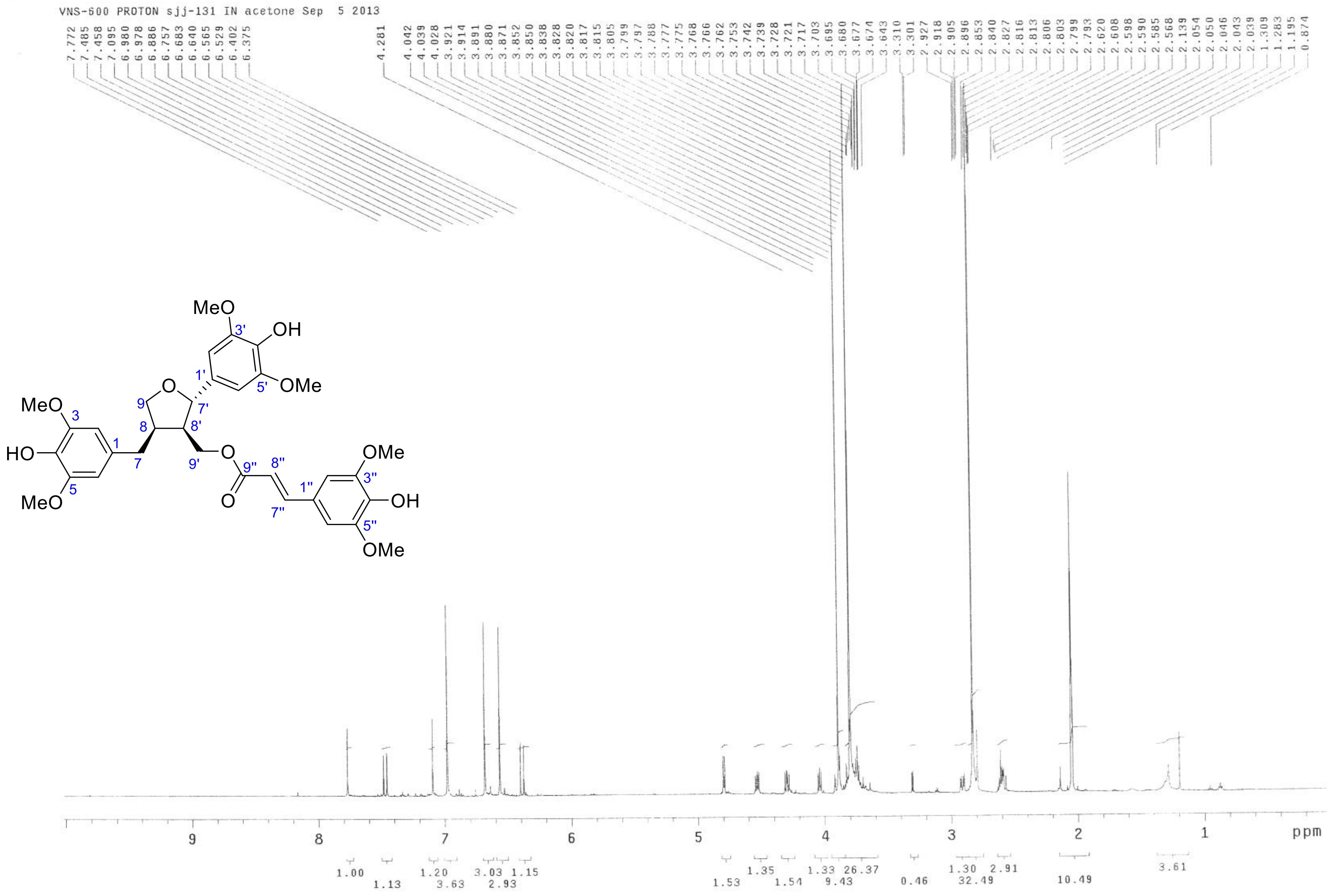

Figure S67.THe ^{1}H NMR Spectrum of Compound 8 in Acetone-d_6 (600 MHz)

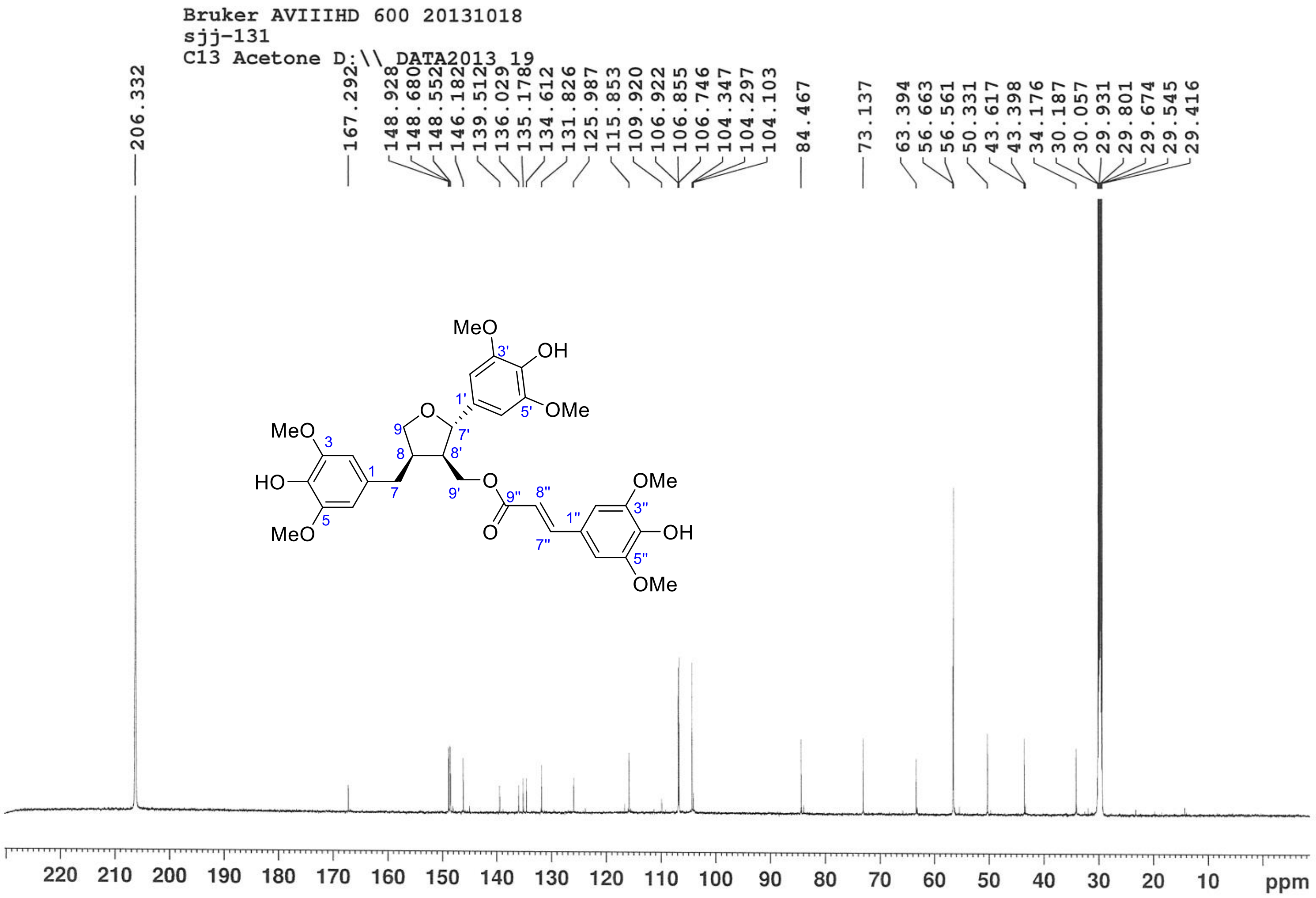

Figure S68.The ^{13}C NMR spectrum of compound 8 in Acetone-d_6 (150 MHz)

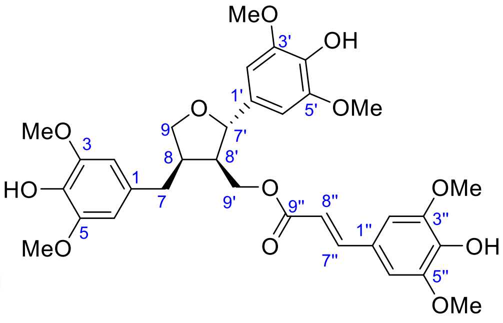

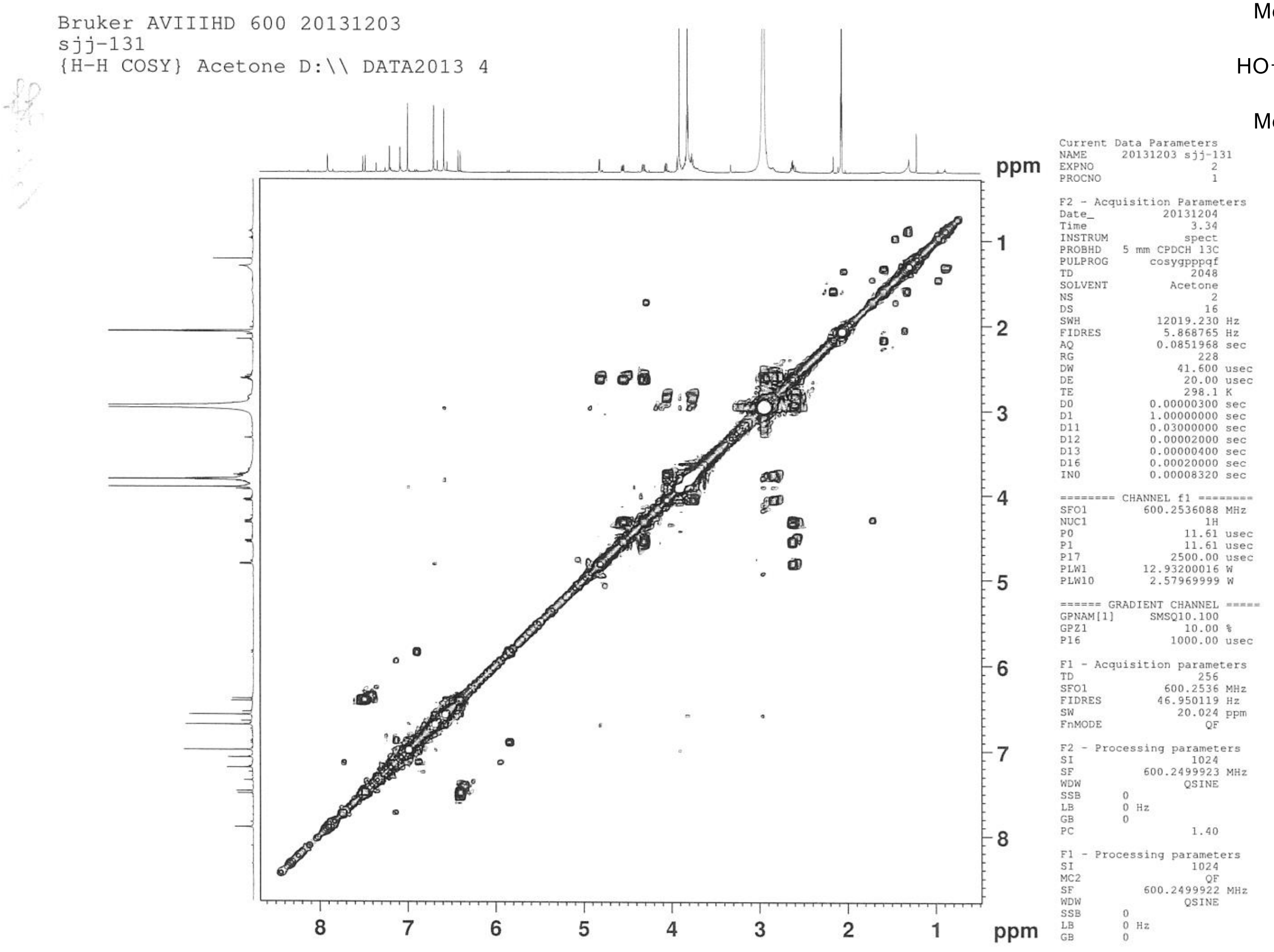

Figure S69. The ^{1}H-^{1}H COSY Spectrum of Compound 8 in Acetone-d_6 (600 MHz)

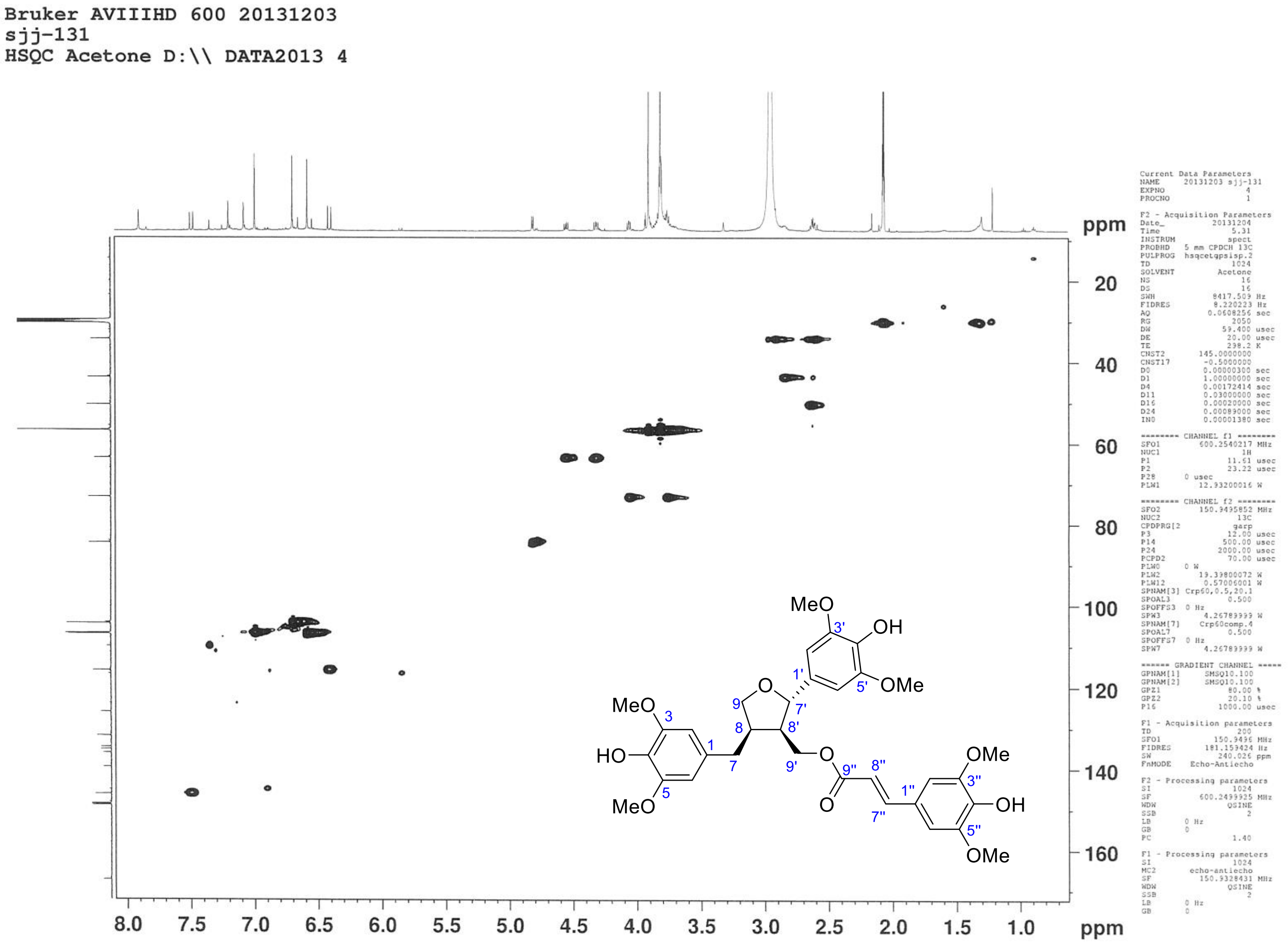

Figure S70. The HSQC Spectrum of Compound 8 in Acetone-d_6 (600 MHz)

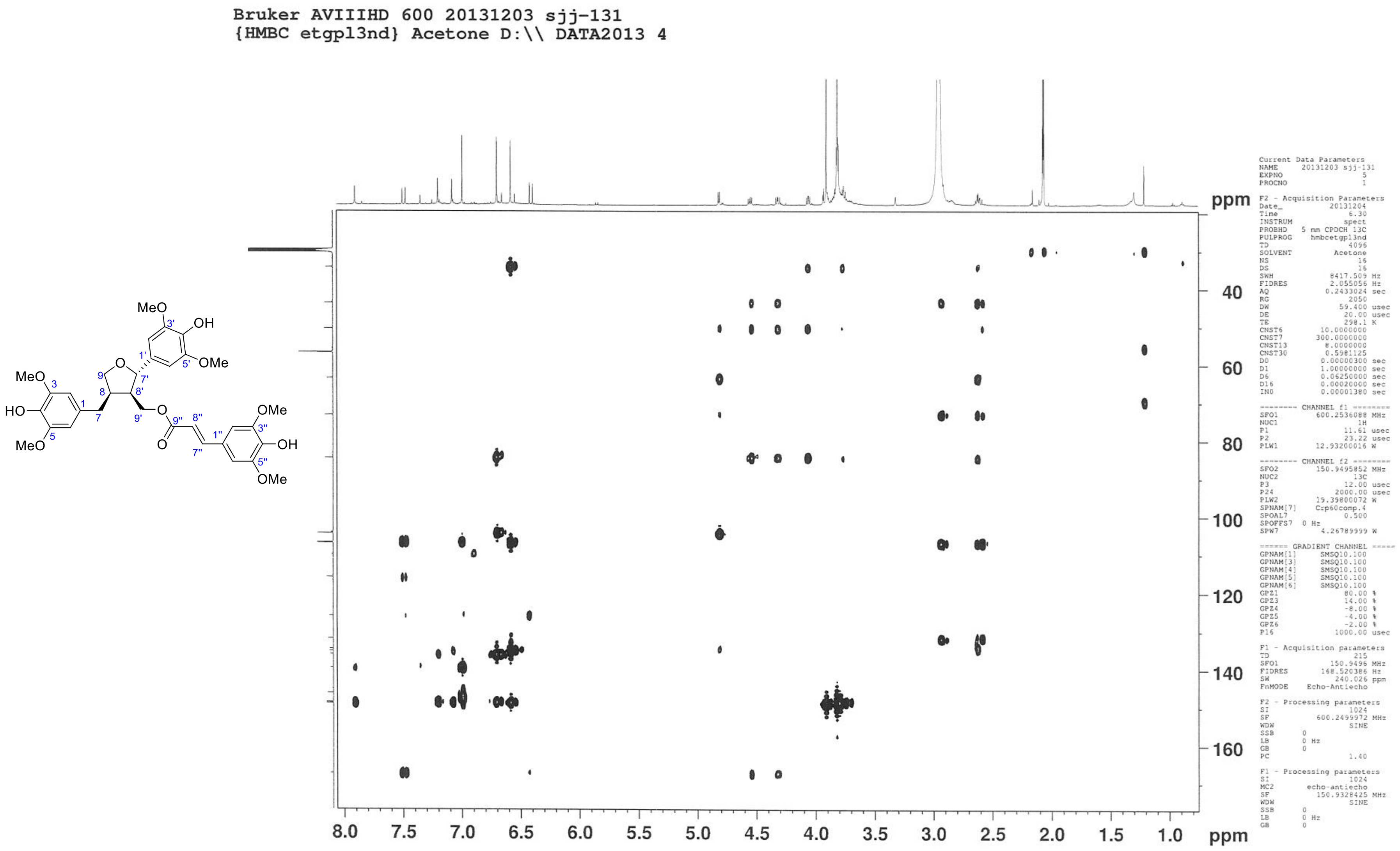

Figure S71. The HMBC Spectrum of Compound 8 in Acetone-d_6 (600 MHz)

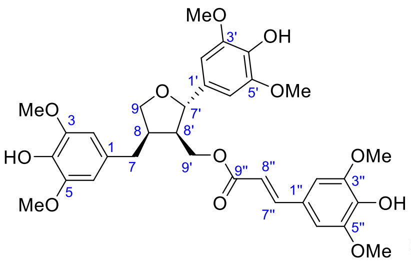

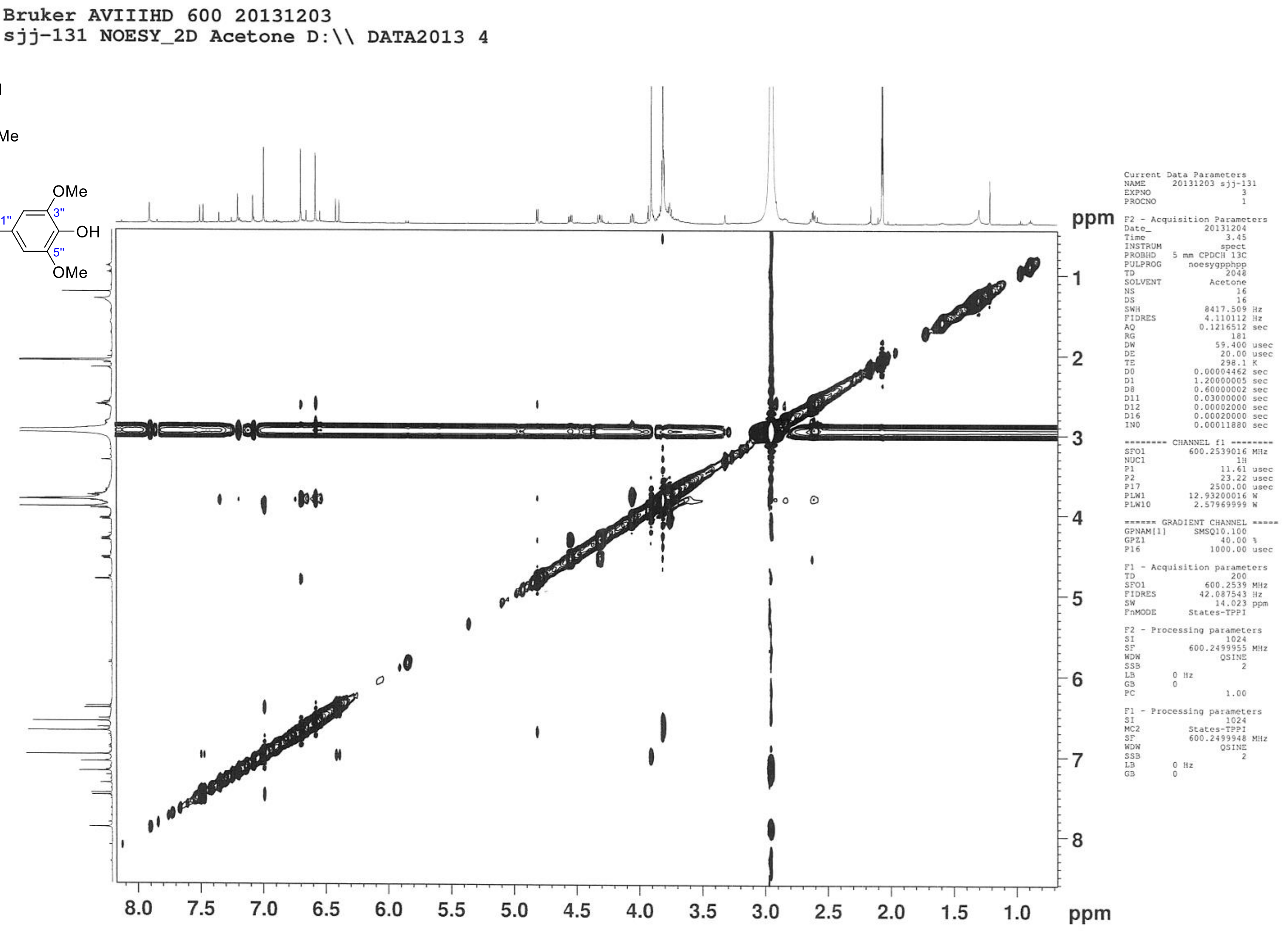

Figure S72. The NOESY Spectrum of Compound 8 in Acetone-d_6 (600 MHz)

Thermo Qexactive Focus Report

compound NO. : sjj-120

Method : LCMS(compound)-low

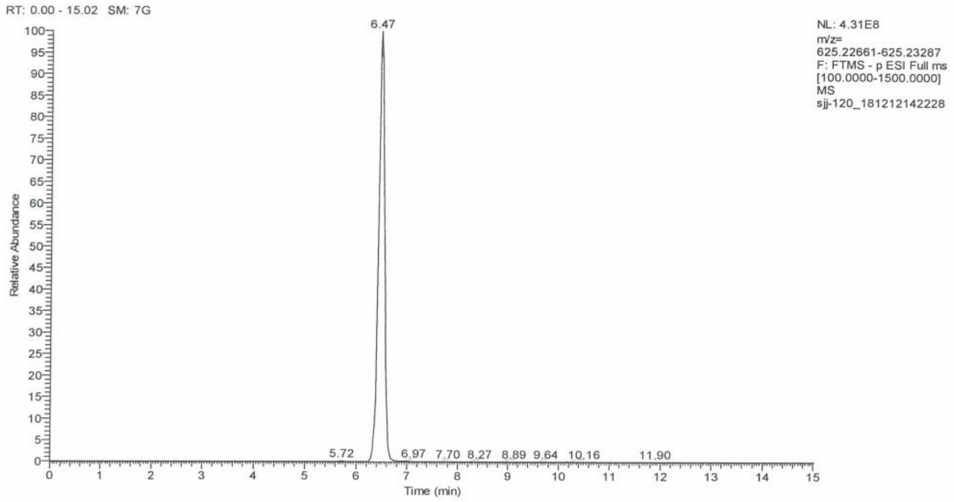

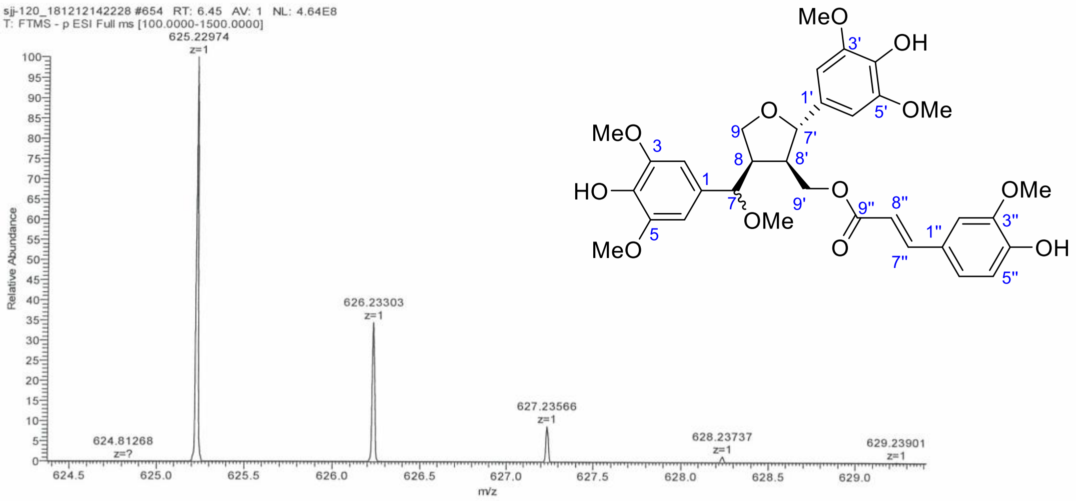

m/z	Theo. Mass	Delta (ppm)	RDB equiv.	Composition	
625.22974	625.22905	1.1	15.5	C33 H37 O12	M-H

Figure S73. TheHR-Mass Spectrum of Compound 9 in MeOH

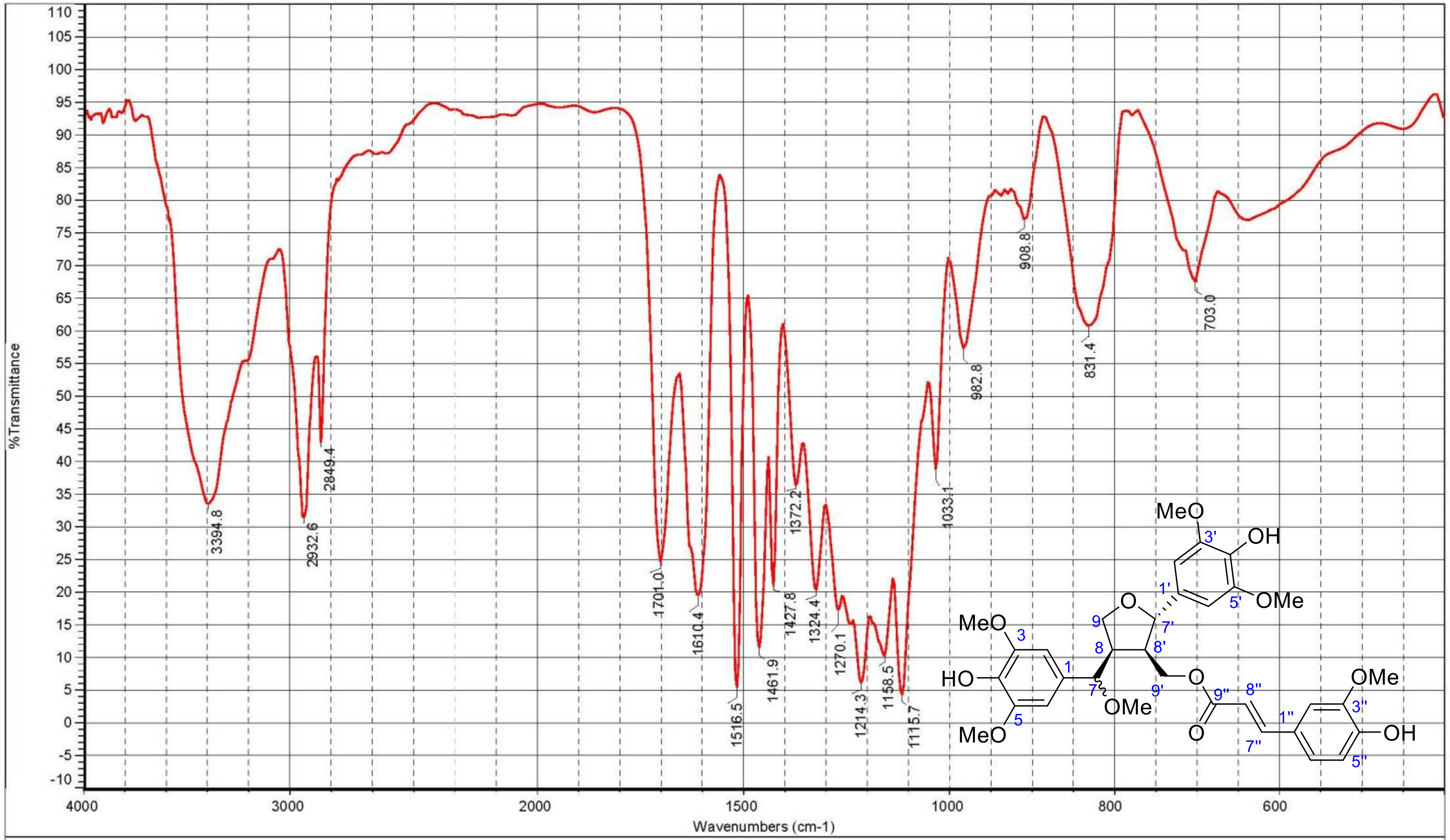

日期: 星期四 1月 16 08:50:17 2014 (GMT+08:00) Sample Name: sjj - 120 (显微镜透射法 FT- IR Microscope Transmission)

扫描次数: 100 傅立叶变换显微镜红外(FT-IR Microscope)：Centaurμs

分辨率: 8.000 美国热电公司(Thermo)傅立叶变换红外光谱仪:Nicolet 5700

Figure S74. The IR Spectrum of Compound 9

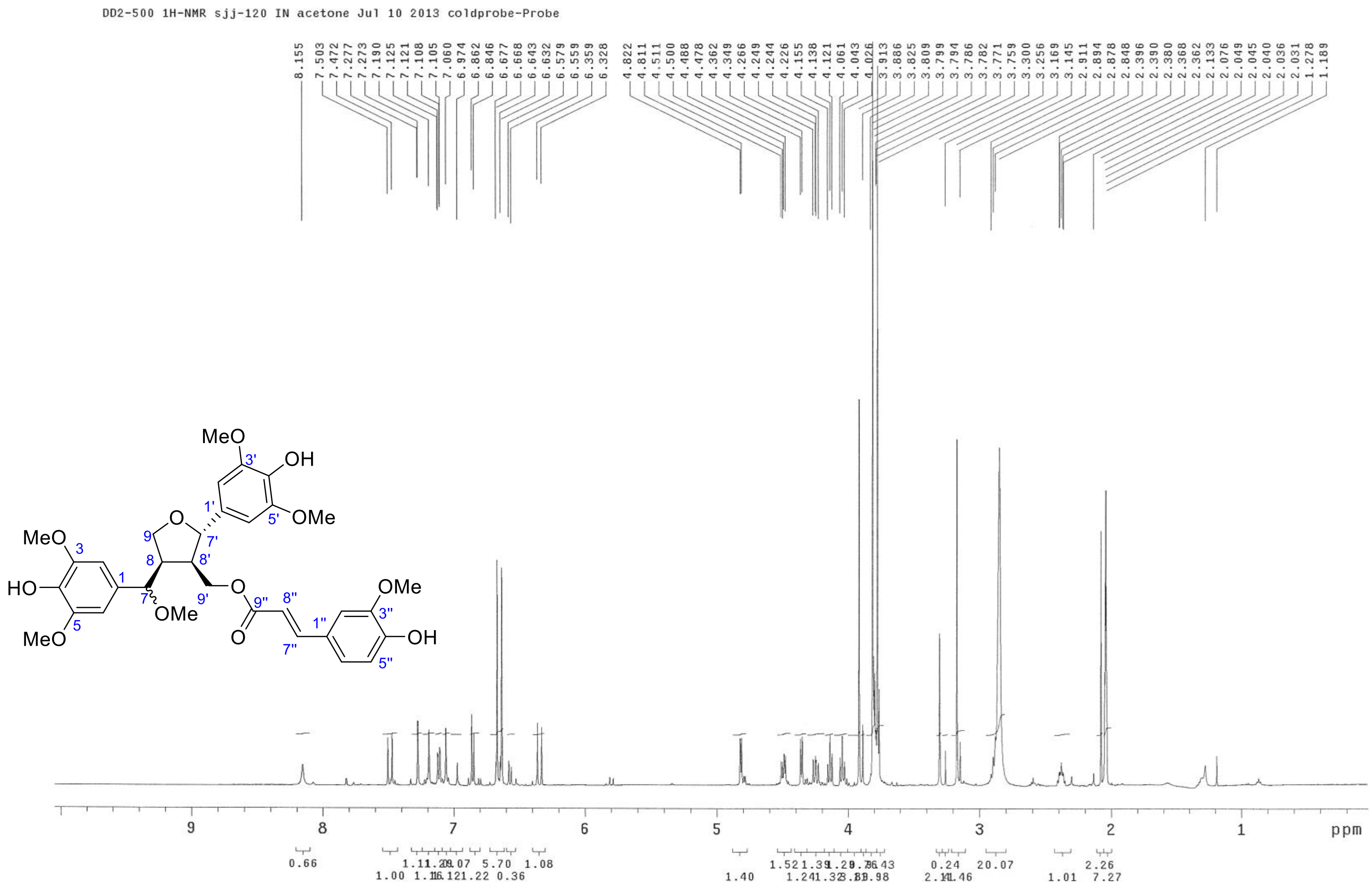

Figure S75.The ^{1}H NMR Spectrum of Compound 9 in Acetone-d_6 (500 MHz)

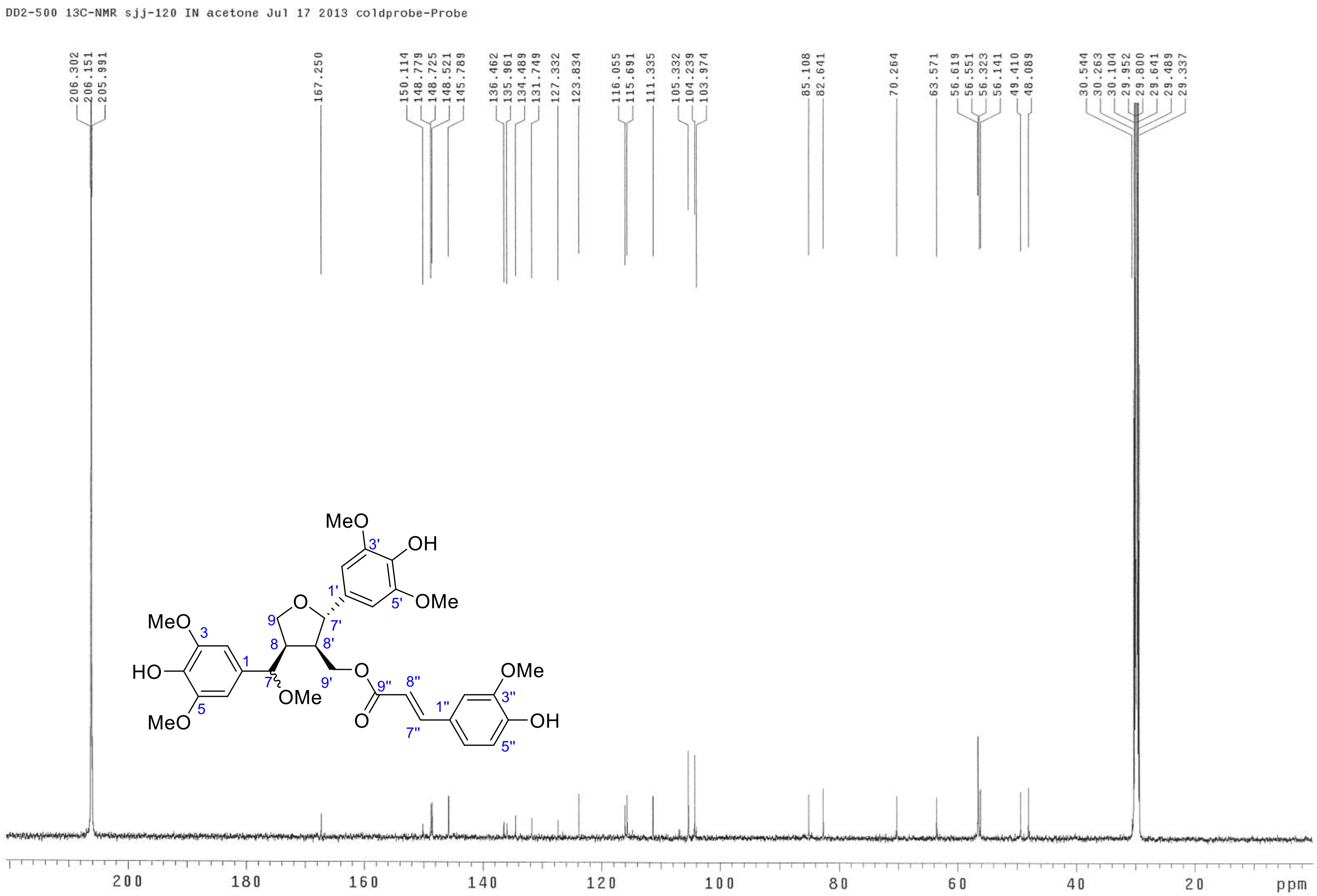

Figure S76.The 13 C NMR Spectrum of Compound 9 in Acetone-d_6 (500 MHz)

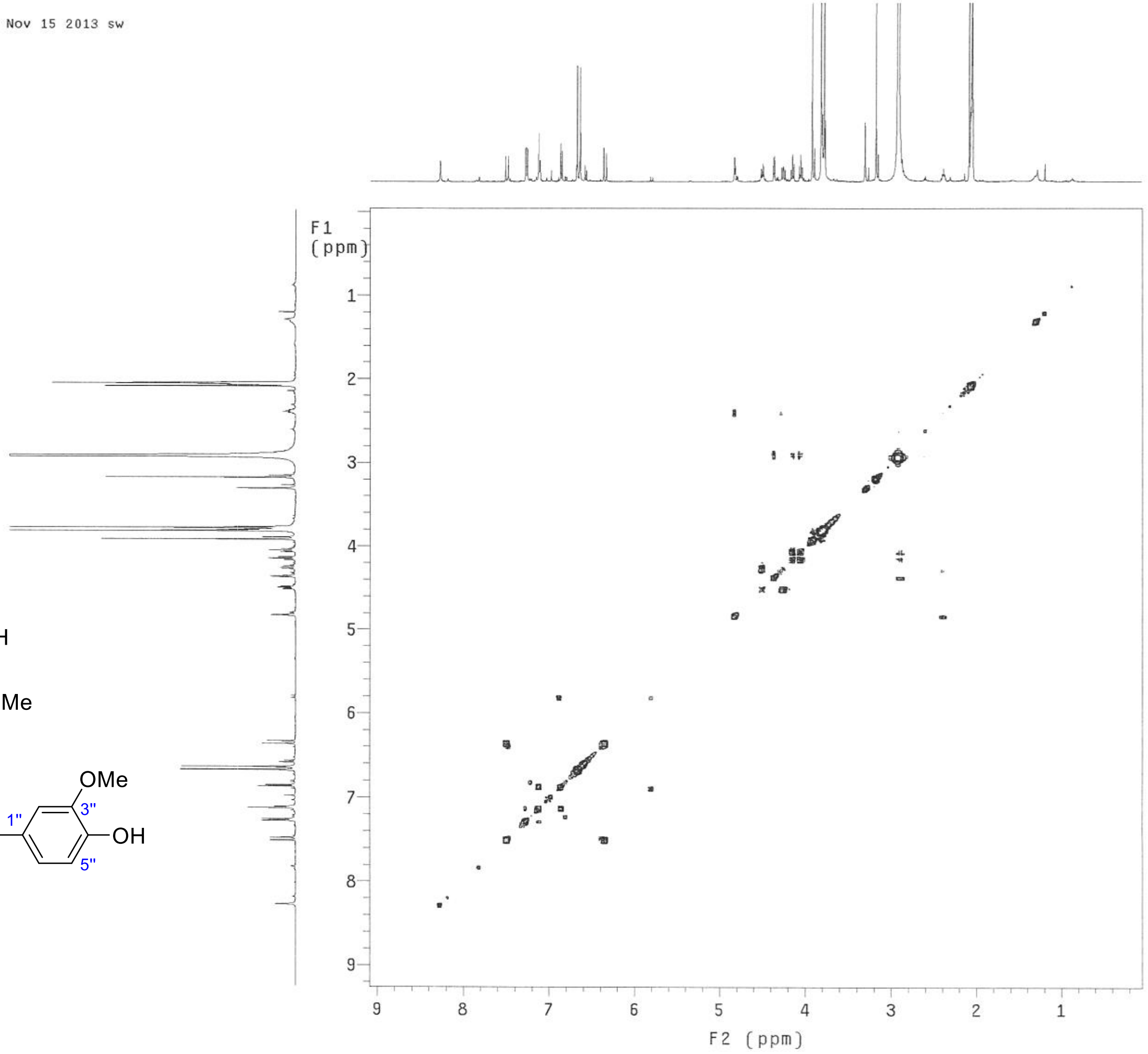

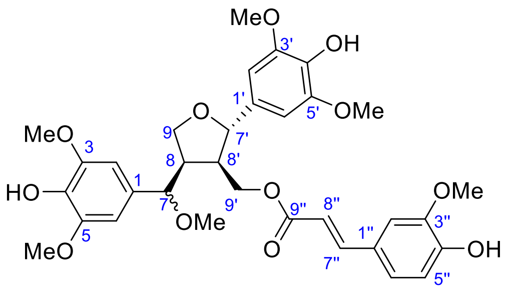

Figure S77. The ^{1}H-^{1}H COSY Spectrum of Compound 9 in Acetone-d_6 (500 MHz)

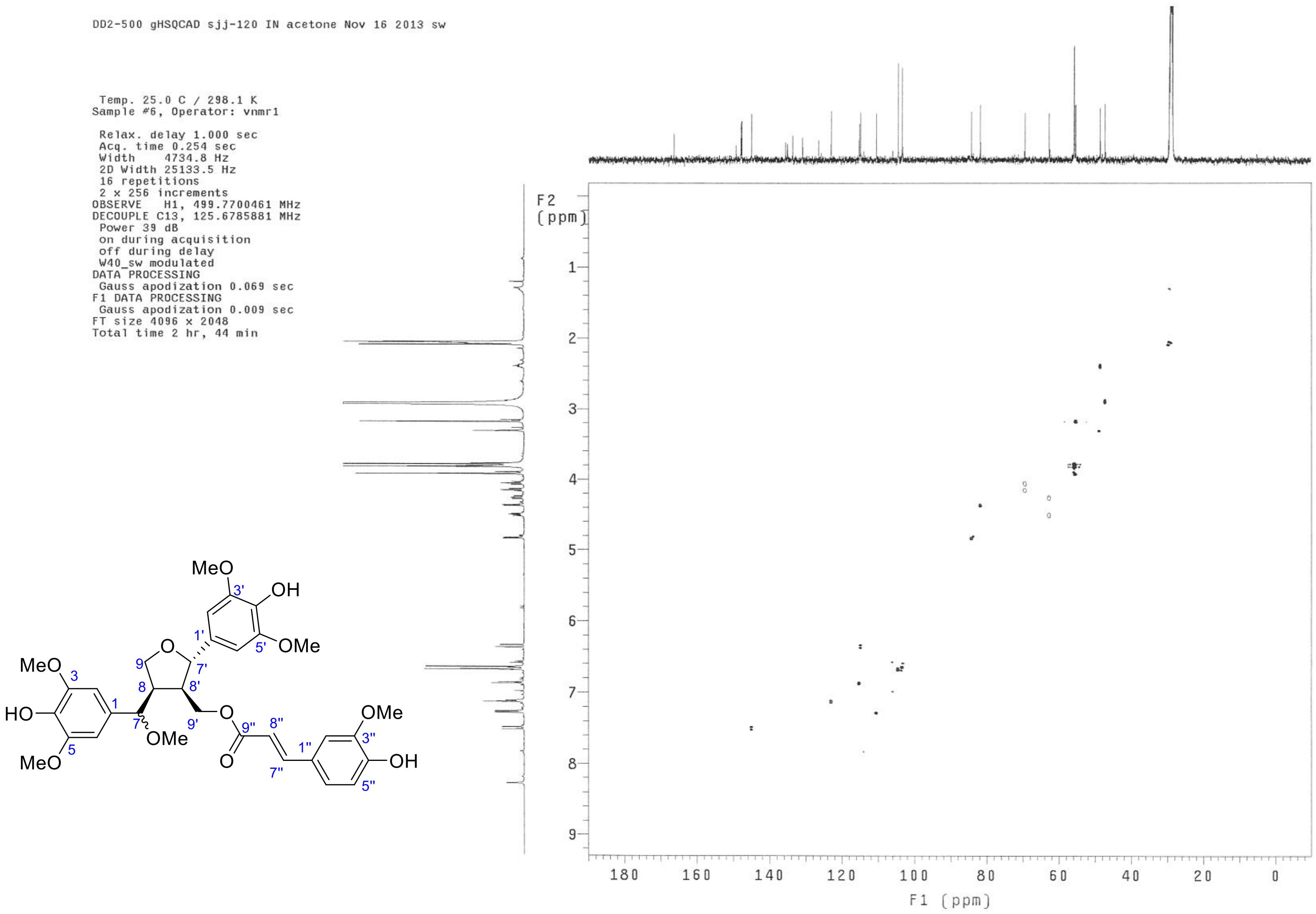

Figure S78. The HSQC Spectrum of Compound 9 in Acetone-d_6 (500 MHz)

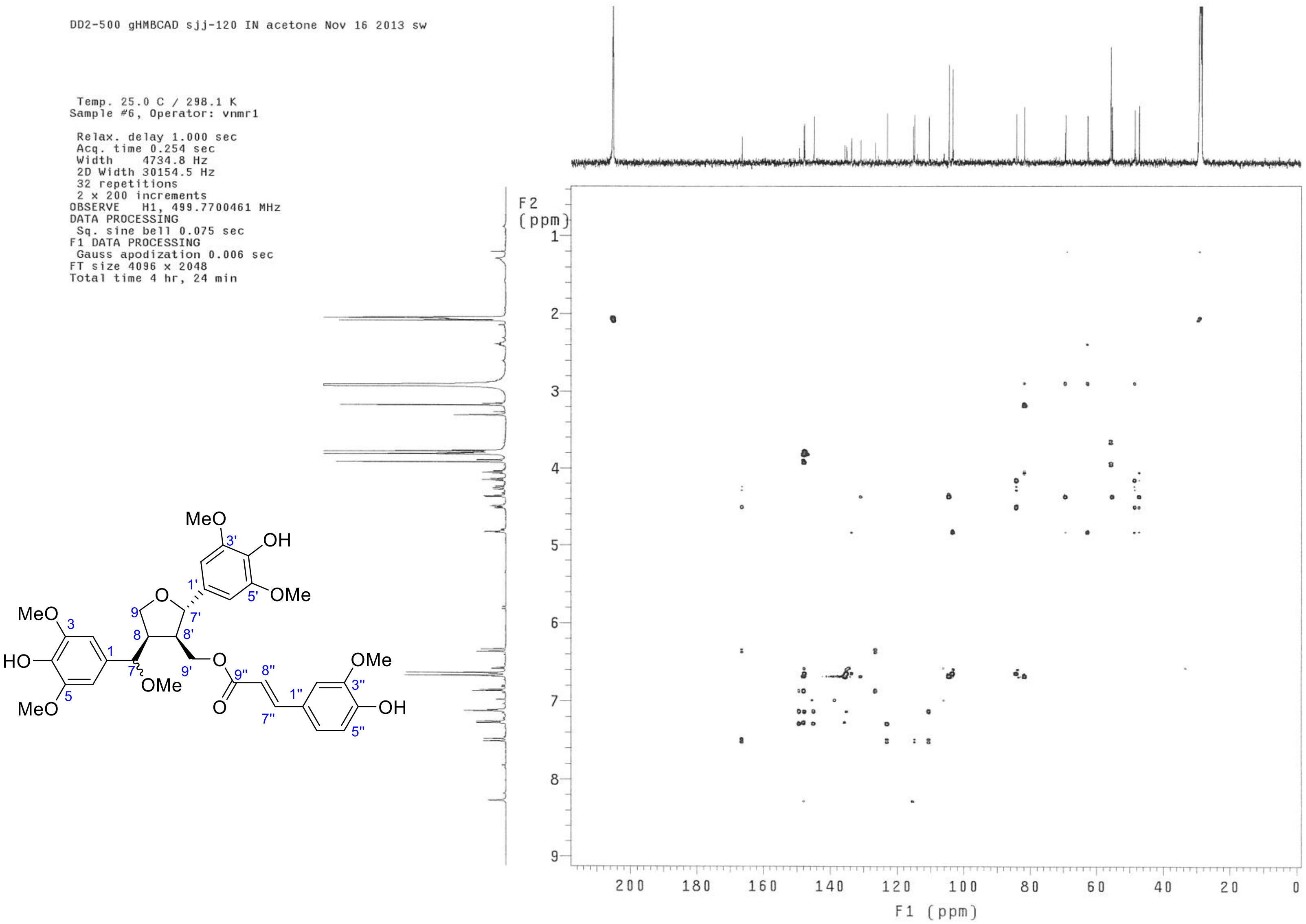

Figure S79. The HMBC Spectrum of Compound 9 in Acetone-d_6 (500 MHz)

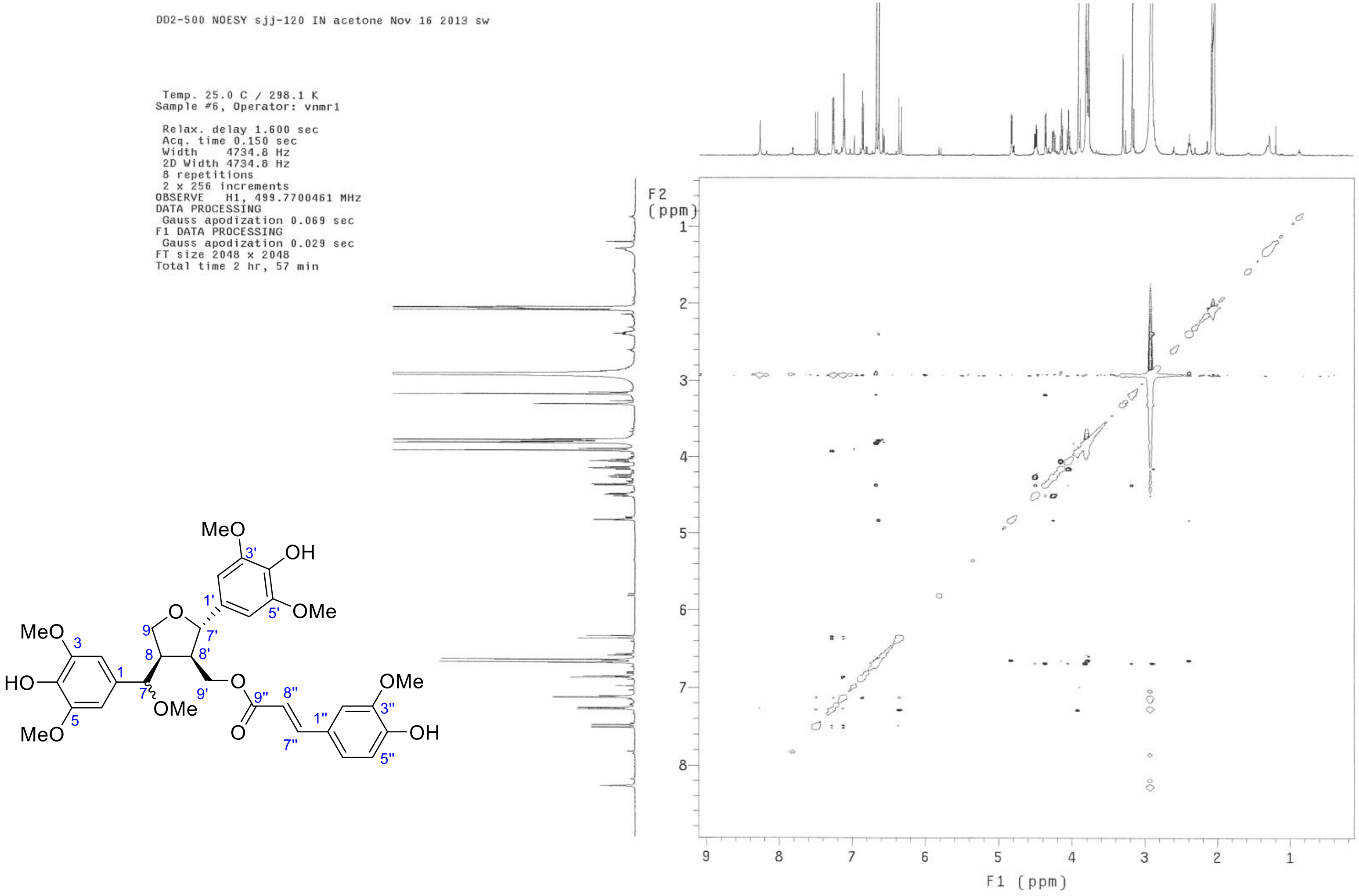

Figure S80. The NOESY Spectrum of Compound 9 in Acetone-d_6 (500 MHz)
